# Four Distinct Dynamic Intracranial Pressure Trajectories and Their Prognostic Implications in Acute Brain Injury: A Multicenter Cohort Study

**DOI:** 10.1002/cns.70735

**Published:** 2026-01-03

**Authors:** Juan Wang, Haibo Li, Manman Xu, Wen‐Juan Li, Longyang Cheng, Shaoya Li, Chun‐Hua Hang, Penglai Zhao

**Affiliations:** ^1^ Department of Neurosurgery Nanjing Drum Tower Hospital, Clinical College of Nanjing University of Chinese Medicine Nanjing China; ^2^ Department of Neurosurgery Nanjing Drum Tower Hospital, The Affiliated Nanjing Drum Tower Hospital of Nanjing University Medical School Nanjing China; ^3^ Neurosurgical Institute Nanjing University Nanjing China; ^4^ Fujian Maternity and Child Health Hospital College of Clinical Medicine for Obstetrics & Gynecology and Pediatrics, Fujian Medical University Fuzhou China; ^5^ Center for Medical Big Data Nanjing Drum Tower Hospital, The Affiliated Nanjing Drum Tower Hospital of Nanjing University Medical School Nanjing China

**Keywords:** acute brain injury, ICP trajectories, latent class growth modeling, mortality prediction, risk stratification

## Abstract

**Background:**

Acute brain injury (ABI) often elevates intracranial pressure (ICP), yet static measurements miss dynamic risk. We evaluated the prognostic value of early ICP trajectories.

**Methods:**

We formed a multicenter ICU cohort from MIMIC‐IV (2008–2022), eICU (2014–2015), and NSICU (2024–2025). Latent class growth modeling identified ICP trajectories over the first 120 h. Associations with in‐hospital mortality were tested with Cox models, and incremental value beyond baseline clinical variables was quantified by AUC, integrated discrimination improvement (IDI), and net reclassification improvement (NRI). Sensitivity analyses were performed across datasets and age groups.

**Results:**

Among 1700 patients with ABI, four trajectories emerged—Severe Progressive, Stabilized Elevated, Mildly Elevated Stable, and Normal. Versus Normal, mortality risk was highest for Severe Progressive (HR 13.54; 95% CI 9.35–19.59), followed by Stabilized Elevated (HR 2.53; 1.83–3.49) and Mildly Elevated Stable (HR 1.48; 1.16–1.91). In patients aged ≥ 55 years, risk with Stabilized Elevated was amplified (HR 2.87; 1.97–4.20). Adding trajectories improved risk stratification (IDI +0.065; NRI +0.201) with modest AUC gains.

**Conclusions:**

Early ICP trajectories define reproducible phenotypes with distinct mortality risk. Incorporating trajectories—particularly the stabilized‐elevated pattern in older adults—adds prognostic value beyond clinical variables and supports prospective validation.

## Graphical abstract

Across a multicenter ICU cohort (*n* = 1700), latent‐class modeling of 0–120 h ICP identified four trajectories. Severe progressive and stabilized‐elevated patterns carried the greatest mortality risk (HR 13.54 and 2.53), with mildly elevated‐stable showing modest risk (HR 1.48). In adults ≥ 55 years, risk with the stabilized‐elevated pattern was amplified (HR 2.87); adding trajectories improved stratification (IDI +0.065; NRI +0.201).

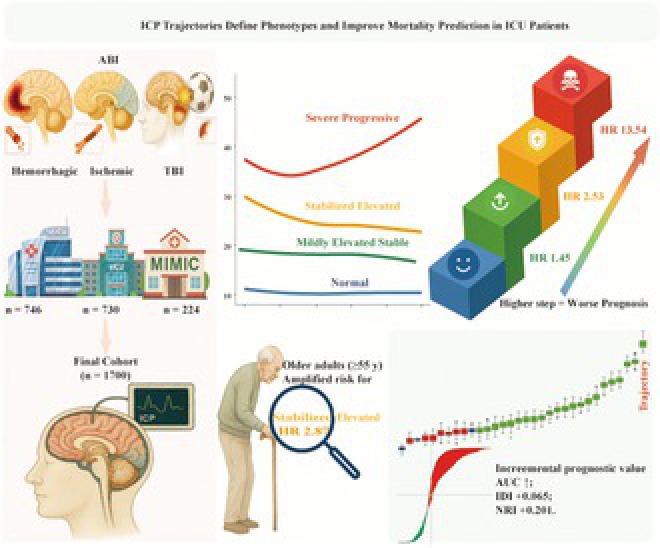

## 1 Introduction

Intracranial pressure (ICP) elevation frequently occurs in patients with acute brain injury (ABI), including traumatic brain injury (TBI), intracerebral hemorrhage (ICH), and ischemic stroke [1, 2]. Prolonged elevated ICP can lead to severe complications such as cerebral herniation and impaired cerebral perfusion, significantly impacting survival and neurological recovery [3]. Inadequate ICP management in neurocritical care may exacerbate secondary brain injury, worsening prognosis and long‐term outcomes [4]. Thus, accurate ICP monitoring is critical for guiding timely interventions aimed at preventing secondary neurological damage and improving patient prognosis.

Traditional ICP monitoring typically relies on fixed thresholds or isolated measurements, providing limited insights into patient‐specific ICP dynamics. Static approaches cannot fully capture ICP variability, limiting their ability to guide precise interventions [5, 6]. In contrast, dynamic trajectory models that track ICP changes over time offer a comprehensive view of cerebral autoregulation and compensatory mechanisms [7, 8]. Such models account for temporal ICP variability, allowing for more individualized clinical decisions. However, the clinical utility of ICP trajectory patterns for predicting in‐hospital mortality and guiding clinical decision‐making remains inadequately explored.

We hypothesized that distinct ICP trajectory patterns are associated with differential risks of in‐hospital mortality in patients with ABI, and that incorporating ICP trajectories into predictive models would enhance clinical risk stratification and support personalized patient management.

## 2 Methods

### 2.1 Database Source

This study utilized data from three well‐established databases: the MIMIC‐IV database (spanning 2008–2022), the eICU Collaborative Research Database (2014–2015), and the NSICU database from Nanjing Drum Tower Hospital. The MIMIC‐IV database is publicly available and contains comprehensive ICU admission records from the Beth Israel Deaconess Medical Center in Boston (2008–2022) [7]. Wang Juan accessed this dataset under certification ID 62674474. The eICU database, compiled between 2014 and 2015, includes multicentre ICU patient data. The NSICU dataset, collected from the Nanjing Drum Tower Hospital, covers a recent period (2023). All data were anonymized to protect patient privacy and to comply with ethical standards, including the Strengthening the Reporting of Observational Studies in Epidemiology (STROBE) guidelines.

### 2.2 Data Collection

#### 2.2.1 Study Population

Inclusion Criteria: This study included adult patients aged 18 years or older who were admitted to the ICU with ABI, including TBI, ICH, and ischaemic stroke.

Exclusion Criteria: Patients younger than 18 years, those with ICU stay of less than 24 h, missing outcome data, absence of ICP measurements or fewer than three ICP measurements during the ICU stay, and multiple ICU admissions (only data from the first admission were included).

#### 2.2.2 Data Collection

We extracted and harmonized data from MIMIC‐IV and eICU‐CRD via SQL/PostgreSQL, and from NSICU via electronic health record (EHR) export. The variables and their units are listed in Table S1. Physiological covariates (vital signs, laboratory values, and urine output) were summarized over the first 24 h of ICU stay to harmonize data across databases and to provide baseline risk adjustments. Comorbidities were derived from the diagnostic codes available for each source. The primary outcome was the in‐hospital mortality rate.

### 2.3 Statistical Analyses

#### 2.3.1 Latent Class Growth Modeling for ICP Trajectory Analysis

Latent class growth modeling (LCGM) was used to identify distinct subgroups of ICP trajectories based on their temporal progression patterns [7, 9]. ICP measurements obtained during the ICU stay were analyzed over three observation windows (72, 120, and 168 h), beginning with the first valid ICP measurement (t = 0). All timestamps were aligned to this origin; accordingly, the 72‐, 120‐, and 168‐h windows reflect the time since monitoring initiation (not ICU admission), enhancing the comparability of physiological time series once monitoring is in place. The measurement intervals were set at 5, 4, and 8 h. All ICP values were standardized in mmHg. Outliers, defined as ICP values < 0 mmHg or > 60 mmHg, were treated as missing data. LCGM used full‐information maximum likelihood to accommodate unbalanced sampling; a minimum of ≥ 3 ICP measurements per patient ensured model identifiability while limiting the exclusion of early deaths/transfers.

Trajectory models with two to six latent classes were evaluated using polynomial functions (linear, quadratic, and cubic) to characterize trajectory shapes. The optimal model was selected according to statistical fit indices, including the Bayesian Information Criterion (BIC), Akaike Information Criterion (AIC), and log‐likelihood (LL), as well as classification accuracy metrics such as the average posterior probability (AvePP) and the odds of correct classification (Occ). Lower BIC and AIC values, along with higher AvePP and Occ values, indicate optimal model fit, classification stability, and clinical interpretability [10, 11, 12].

#### 2.3.2 Feature Selection and Model Development

Five feature selection methods were applied to identify predictors of in‐hospital mortality: stepwise regression, Least Absolute Shrinkage and Selection Operator (LASSO), Boruta, and Best Subset Selection using adjusted *R* ^2^ and Mallows Cp [13, 14]. Stepwise regression selects variables based on the AIC. LASSO regression, optimized through 10‐fold cross‐validation, overfitting by retaining the most predictive variables. Best Subset Selection identified optimal variable subsets based on Mallows' Cp (optimal Cp ≈ *p* + 1) and adjusted *R* ^2^. Boruta, a random‐forest‐based method, selects important features by comparing their importance with that of the permuted shadow variables. Using the variables identified using these feature selection methods, two predictive models were developed: a Baseline Model and the Trajectory Model. The Baseline Model included the clinical and demographic variables selected using the aforementioned methods. The Trajectory Model extends the Baseline Model by incorporating the ICP trajectory patterns derived from the LCGM.

#### 2.3.3 Model Comparison and Evaluation Metrics

The performances of the Baseline and Trajectory Models were evaluated using both merged (MIMIC‐IV, eICU, and NSICU) and individual datasets. The evaluation metrics included the Median Improvement in Risk Score, Integrated Discrimination Improvement (IDI), Net Reclassification Improvement (NRI), and Area Under the Curve (AUC).

Statistical analyses were performed using R and the Free Statistics platform [15, 16]. Statistical significance was defined as *p* < 0.05. For all continuous variables, the distributional shape was assessed using the Shapiro–Wilk test and *Q*–*Q* plots. Accordingly, between‐trajectory comparisons were conducted primarily using the Kruskal–Wallis test followed by Dunn's test with Holm correction; one‐way ANOVA with Tukey's HSD was used only when assumptions appeared approximately satisfied and yielded concordant inferences. Missing data were handled with multiple imputations using chained equations (MICE; *m* = 10), with estimates pooled using Rubin's rules. Kaplan–Meier curves were generated, and differences between groups were assessed using the log‐rank test. To address potential confounding, candidate covariates were selected a priori based on clinical relevance and prior literature, and were further screened using a change‐in‐estimate criterion (retained if any trajectory HR changed by ≥ 10%). Cox proportional hazards models were used to estimate hazard ratios (HRs) for in‐hospital mortality across ICP trajectory classes (trajectory 1 as a reference). The primary analysis used a 120‐h window (4‐h sampling), with 72‐ and 168‐h windows treated as sensitivity analyses. Models were adjusted for the pre‐specified covariates listed in Table 4, and the same adjustment set was applied across analyses. Collinearity was assessed using variance inflation factors (VIF < 5) and proportional hazard assumptions with Schoenfeld residuals. Subgroup analyses were conducted on the merged dataset to identify potential effect modifiers influencing the association between ICP trajectories and in‐hospital mortality. The subgroups were defined based on age (< 55 vs. ≥ 55 years), traumatic brain injury, hypertension (HBP), craniotomy, and embolization.

## 3 Results

This study analyzed ICP trajectories in patients with ABI using data from three multicenter ICU datasets: MIMIC‐IV (2008–2022), eICU (2014–2015), and NSICU (2024–2025). A total of 14,274 patients from MIMIC‐IV, 17,578 from eICU, and 1666 from NSICU were screened. After applying the exclusion criteria, the final cohort consisted of 746 patients from MIMIC‐IV, 730 from eICU, and 224 from NSICU, yielding a total of 1700 patients (Figure S1).

### 3.1 Characterization of Four ICP Trajectories

The four‐class ICP trajectory model consistently provided the best fit across the three evaluated periods (72‐, 120‐, and 168‐h windows), as demonstrated by its lowest AIC and BIC values, achieving an optimal balance between model complexity and fit (Table S2A–C). The classification quality in the 72‐h window was acceptable (entropy 0.68; class‐wise average posterior probabilities 0.86, 0.68, 0.83, and 0.91; Table S2A). In contrast, five‐ or six‐trajectory models increased the complexity without substantial gains, limiting their clinical applicability. Further validation across individual datasets (Table S2D–L) confirmed the consistency and reliability of the four‐group classifications. Given the alignment with clinical practice and literature, a 120‐h monitoring period (measurements every 4 h) was selected for subsequent analyses [8, 17].

**FIGURE 1:**
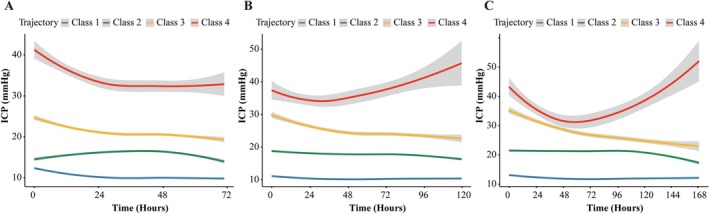
LCGM‐derived ICP trajectories over 72, 120, and 168 h. The curves display the class‐specific mean ICP trajectories with 95% confidence intervals (shaded bands), estimated by LCGM from an integrated dataset. Panels A–C depict the trajectories over 72 h (at 5‐h intervals), 120 h (at 4‐h intervals), and 168 h (at 8‐h intervals), respectively. The four identified trajectories are defined as: Trajectory 1—low and stable (blue); Trajectory 2—mildly elevated and stable (green); Trajectory 3—elevated with a stabilizing plateau (yellow); and Trajectory 4—high and progressively rising with greater variability (red).

After establishing the optimal four‐class ICP trajectory model, Figure 1 illustrates the identified ICP trajectories across the merged datasets (MIMIC‐IV, eICU, and NSICU) at 72, 120, and 168 h, whereas Figure S2 shows the distribution of trajectory classes across datasets and time windows. These trajectories represent distinct ICP dynamics with varying progression patterns and cerebral perfusion pressures (CPP; Figure S4): Trajectory 1 (*Normal ICP*), characterized by stable ICP consistently below 20 mmHg, maintaining adequate cerebral perfusion; Trajectory 2 (*Mildly Elevated ICP*), ICP levels of 15–25 mmHg with stable progression and preserved CPP; Trajectory 3 (*Stabilized Elevated ICP*), persistently elevated ICP around 26 mmHg with stabilized progression but reduced CPP below 50 mmHg; and Trajectory 4 (*Severe Progressive ICP*), marked by sustained and highly variable ICP, peaks exceeding 50 mmHg, and severely compromised cerebral perfusion (CPP ≤ 40 mmHg).

### 3.2 Clinical Characteristics Stratified by Four ICP Trajectories

Table 1 summarizes the baseline characteristics of the integrated cohort (per‐dataset details are provided in Table S3A–C). Patients in Trajectory 1 were older, whereas those in Trajectory 4 were the youngest; the sex distribution was similar across groups. Trajectory 2 exhibited the highest BMI. Physiological markers of acute illness were most pronounced in Trajectory 4 (higher respiratory and heart rates, higher MBP and WBC, and lower temperature), together with slightly higher creatinine and greater Day‐1 urine output. Admission patterns and etiology also differed, with a larger proportion of traumatic cases in Trajectories 2–4. Early supportive therapies showed group differences; vasopressor and mannitol use were more frequent in Trajectory 4, and intubation rates differed across groups. Overall, these baseline profiles indicate a graded increase in severity from Trajectories 1–4, consistent with the outcome gradient and the patterns in Figures S3–S6.

**TABLE 1:** Baseline characteristics by ICP trajectory in the integrated dataset.

Characteristic	Total (*n* = 1700)	Trajectory 1 (*n* = 702)	Trajectory 2 (*n* = 723)	Trajectory 3 (*n* = 205)	Trajectory 4 (*n* = 70)	*p*
Age, years	56.8 ± 17.1	62.8 ± 14.9	52.9 ± 17.0	52.2 ± 18.2	50.3 ± 17.8	< 0.001
Sex, male	933 (54.9)	363 (51.7)	419 (58)	116 (56.6)	35 (50)	0.089
BMI	26.6 (23.4, 30.8)	26.0 (23.0, 30.1)	27.3 (24.0, 31.5)	26.7 (23.6, 30.8)	25.0 (22.2, 29.4)	< 0.001
Respiratory rate, bpm	20.7 ± 9.8	18.9 ± 5.8	21.7 ± 11.2	21.6 ± 12.2	25.3 ± 15.1	< 0.001
Heart rate, bpm	92.6 ± 26.1	84.6 ± 18.4	97.6 ± 28.6	99.3 ± 31.3	100.7 ± 27.6	< 0.001
MBP, mmHg	99.5 ± 35.5	97.1 ± 29.9	99.4 ± 37.2	103.9 ± 43.2	112.6 ± 41.0	0.001
Temperature, °C	36.8 ± 0.9	36.9 ± 0.6	36.7 ± 1.0	36.6 ± 1.3	36.3 ± 1.2	< 0.001
Urine output, mL	1905.0 (1246.5, 2852.5)	1750.0 (1205.0, 2455.0)	2066.9 (1302.5, 3045.0)	2198.4 (1368.8, 3490.7)	2326.2 (1462.4, 4635.6)	< 0.001
ALT, U/L	23.8 (16.0, 40.0)	20.0 (14.0, 31.0)	27.0 (17.0, 47.0)	24.0 (16.0, 42.8)	27.0 (21.0, 56.9)	< 0.001
BUN, mg/dL	17.3 ± 9.5	17.9 ± 9.5	16.8 ± 9.2	16.9 ± 10.6	17.4 ± 8.4	0.168
Creatinine, mg/dL	0.9 (0.7, 1.1)	0.9 (0.7, 1.1)	0.9 (0.7, 1.1)	0.9 (0.7, 1.1)	1.0 (0.8, 1.2)	0.005
PLT, ×10^9^/L	196.4 ± 76.2	197.4 ± 83.3	197.7 ± 70.7	192.5 ± 70.4	185.1 ± 72.9	0.5
RBC, 10^12^/L	3.8 ± 0.8	3.8 ± 0.8	3.8 ± 0.8	3.8 ± 0.8	3.6 ± 0.9	0.236
WBC, 10^9^/L	15.1 ± 6.5	14.1 ± 6.6	15.5 ± 6.3	15.6 ± 6.3	18.0 ± 7.9	< 0.001
Admission type, emergency	1313 (77.2)	610 (86.9)	511 (70.7)	138 (67.3)	54 (77.1)	< 0.001
Admission time						0.019
Day shift	1110 (65.4)	485 (69.3)	457 (63.2)	121 (59)	47 (67.1)	
Night shift	588 (34.6)	215 (30.7)	266 (36.8)	84 (41)	23 (32.9)	
Traumatic	482 (28.4)	100 (14.2)	263 (36.4)	87 (42.4)	32 (45.7)	< 0.001
Hypertension	888 (52.2)	453 (64.5)	326 (45.1)	79 (38.5)	30 (42.9)	< 0.001
Diabetes	224 (13.2)	104 (14.8)	94 (13)	18 (8.8)	8 (11.4)	0.15
Liver disease	77 (4.5)	46 (6.6)	26 (3.6)	5 (2.4)	0 (0)	0.006
Stroke history	157 (9.2)	84 (12)	49 (6.8)	14 (6.8)	10 (14.3)	0.002
Initial GCS	8 ± 4	7 ± 4	8 ± 4	8 ± 5	7 ± 5	0.009
Intubation	1068 (62.8)	488 (69.5)	415 (57.4)	121 (59)	44 (62.9)	< 0.001
Vasopressor	395 (23.2)	124 (17.7)	173 (23.9)	64 (31.2)	34 (48.6)	< 0.001
Dialysis	30 (1.8)	11 (1.6)	12 (1.7)	5 (2.4)	2 (2.9)	0.579
Mannitol	348 (20.5)	144 (20.5)	129 (17.8)	45 (22)	30 (42.9)	< 0.001
Craniotomy	1051 (61.8)	460 (65.5)	442 (61.1)	112 (54.6)	37 (52.9)	0.011
Embolization	437 (25.7)	223 (31.8)	170 (23.5)	35 (17.1)	9 (12.9)	< 0.001

*Note:* Missing data: Variables with < 10% missing data include Admission Time, BUN, Creatinine, Initial GCS, Heart Rate, MBP, PLT, RBC, Respiratory Rate, Temperature, and WBC. Variables with 10%–20% missing data include BMI and Urine Output. ALT is the only variable with 30%–50% missing data. The variables Urine Output, Vasopressor, and Mannitol specifically refer to values recorded on Day 1 of ICU admission.

Abbreviations: ALT, alanine aminotransferase; BMI, body mass index; bpm, breaths/beats per minute; BUN, blood urea nitrogen; GCS, Glasgow Coma Scale (initial assessment upon admission); ICP, intracranial pressure; MBP, mean blood pressure; PLT, platelet; RBC, red blood cell; WBC, white blood cell.

Figures S3–S6 show distinct clinical profiles across ICP trajectories. Trajectory 4 showed a less favorable profile, with a lower GCS score, the highest ICP levels, the poorest CPP, and a higher likelihood of discharge to intensive or long‐term care. In contrast, Trajectory 1 had a more favorable overall profile, with lower ICP, preserved CPP, and higher rates of home discharge. Trajectory 3 exhibited intermediate severity and tended towards more nonhome discharges than Trajectory 1.

### 3.3 ICP Trajectories and In‐Hospital Mortality

**FIGURE 2:**
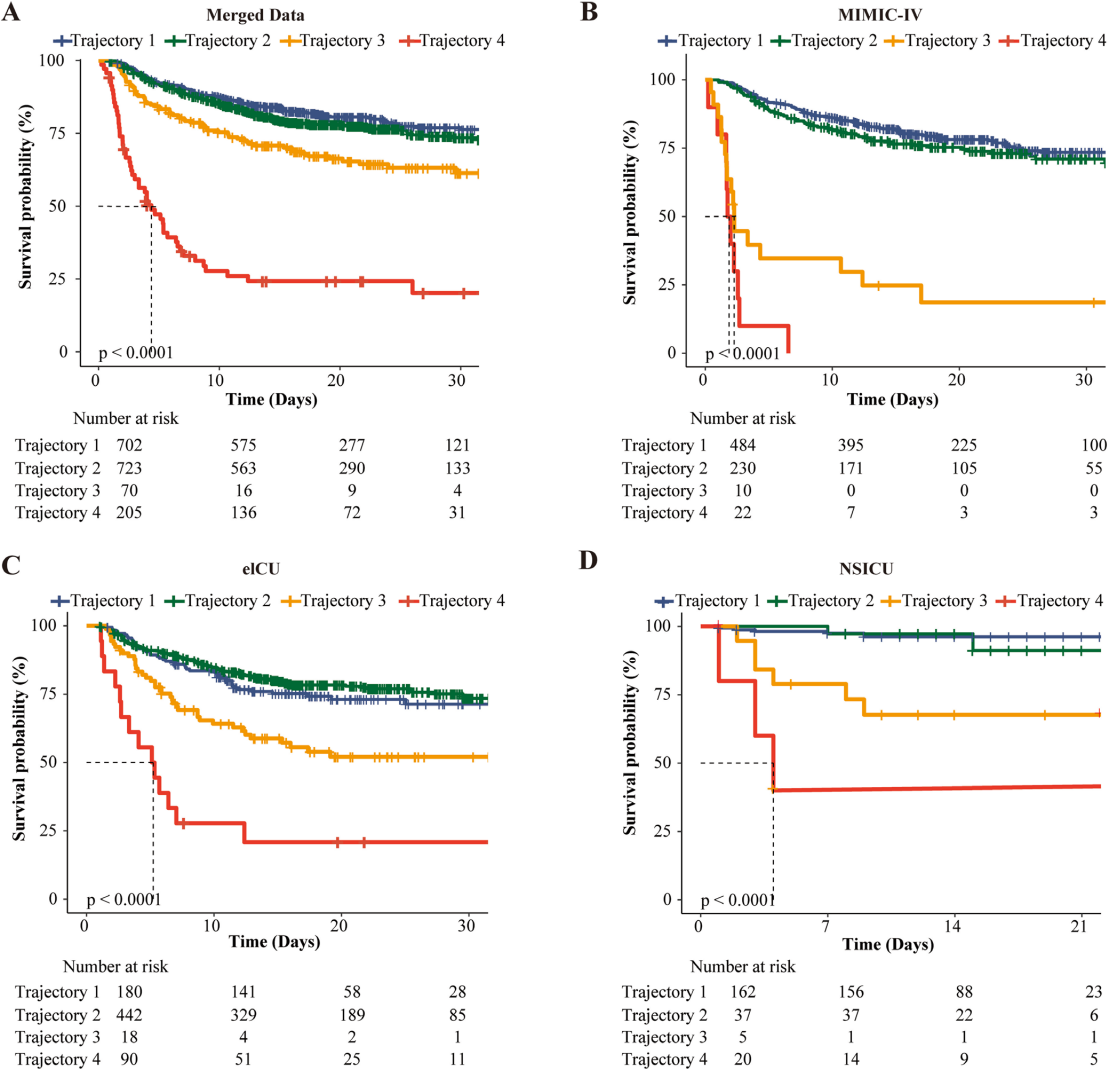
Kaplan–Meier Survival by ICP trajectory class. Kaplan–Meier curves comparing survival probability across the four ICP trajectories in the merged dataset (A) and the individual MIMIC‐IV (B), eICU (C), and NSICU (D) cohorts. Vertical ticks on the curves indicate censored observations. The tables below each plot list the number of patients at risk at specific time intervals. *p*‐values are derived from log‐rank tests comparing the survival distributions. The dashed lines indicate the *median survival time* for specific trajectories.

As shown in Table 2, Cox regression analysis was used to assess the association between ICP trajectory classes (every 4 h over 120 h) and in‐hospital mortality across multiple ICU settings. After adjusting for covariates (Model 2; covariate selection summarized in Table S4), Trajectory 2 (mildly elevated ICP) was associated with increased mortality risk compared with Trajectory 1 (normal ICP), with an HR of 1.48 (95% CI: 1.16–1.91). Trajectory 3 (stabilized elevated ICP) and Trajectory 4 (severe progressive ICP) demonstrated substantially higher mortality risks, with HRs of 2.53 (95% CI: 1.83–3.49) and 13.54 (95% CI: 9.35–19.59), respectively. Consistent with these associations, Figure 2 shows a clear, stepwise separation of Kaplan–Meier survival curves across trajectory classes in the merged and individual datasets (log‐rank *p* < 0.001).

**TABLE 2:** Multivariable analysis of the association between ICP trajectory class and in‐hospital mortality.

ICP trajectories	Merged data				MIMIC‐IV				eICU				NSICU			
	Model 1		Model 2		Model 1		Model 2		Model 1		Model 2		Model 1		Model 2	
	HR (95% CI)	*p*	HR (95% CI)	*p*	HR (95% CI)	*p*	HR (95% CI)	*p*	HR (95% CI)	*p*	HR (95% CI)	*p*	HR (95% CI)	*p*	HR (95% CI)	*p*
Trajectory 1 (reference)	REF	—	—	—	—	—	—	—	—	—	—	—	—	—	—	—
Trajectory 2	1.16 (0.92–1.45)	0.21	1.48 (1.16–1.91)	0.002	1.16 (0.85–1.58)	0.363	1.87 (1.32–2.64)	0.001	0.89 (0.62–1.26)	0.498	0.94 (0.66–1.35)	0.75	1.97 (0.51–7.65)	0.328	6.04 (0.89–40.89)	0.065
Trajectory 3	1.84 (1.38–2.47)	< 0.001	2.53 (1.83–3.49)	< 0.001	8.09 (4.85–13.49)	< 0.001	11.01 (6.25–19.38)	< 0.001	2.03 (1.32–3.12)	0.001	2.29 (1.41–3.74)	0.001	7.41 (2.46–22.31)	< 0.001	4.86 (1.11–21.23)	0.036
Trajectory 4	8.33 (6.03–11.52)	< 0.001	13.54 (9.35–19.59)	< 0.001	34.33 (17.26–68.29)	< 0.001	30.39 (14.12–65.4)	< 0.001	6.29 (3.44–11.49)	< 0.001	8.03 (4.24–15.22)	< 0.001	30.87 (8.53–111.71)	< 0.001	61.51 (7.07–535.14)	< 0.001
Mean ICP	1.05 (1.04–1.06)	< 0.001	1.07 (1.06–1.08)	< 0.001	1.07 (1.06–1.09)	< 0.001	1.08 (1.06–1.09)	< 0.001	1.05 (1.03–1.06)	< 0.001	1.06 (1.04–1.08)	< 0.001	1.08 (1.06–1.11)	< 0.001	1.07 (1.04–1.11)	< 0.001

*Note:* ICP recorded every 4 h for 120 h from the first valid reading. Cox PH models report HR (95% CI) vs. Trajectory 1. Model 1 unadjusted; Model 2 adjusted for age, heart rate, temperature, urine output, creatinine, WBC, admission type, traumatic etiology, hypertension, initial GCS, intubation, vasopressor, craniotomy, embolization.

### 3.4 Identification and Characterization of ICP Trajectories in Older Adult Patients

**FIGURE 3:**
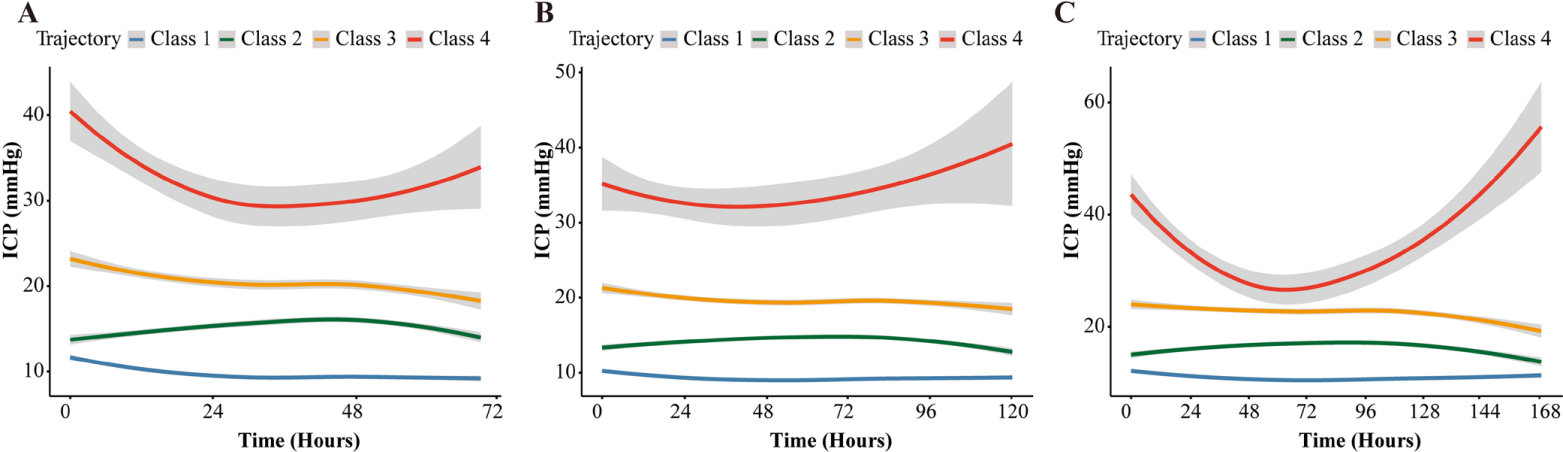
ICP trajectories in elderly patients. LCGM‐derived ICP trajectories for patients aged ≥ 55 years from the integrated dataset, shown over 72 (A), 120 (B), and 168 h (C). The curves represent the class‐specific mean ICP, whereas the shaded areas indicate the 95% CIs.

As shown in Figure S7 (*p* for interaction = 0.001) and Table S5A–C, given the significant age and trajectory interaction, the trajectory model was reapplied to patients aged ≥ 55 years. In contrast, no significant interaction was observed for traumatic etiology across datasets, and the stepwise mortality gradient across trajectories remained directionally consistent within both the traumatic and nontraumatic strata. As illustrated in Figure 3 and Figure S8, the four‐group classification consistently provided an optimal fit across the 72‐, 120‐, and 168‐h monitoring periods. Statistical comparisons (Table S6A–L) confirmed that this model achieved the best balance between model fit, complexity, and interpretability, as indicated by lower BIC values compared with simpler (two‐ and three‐class) and more complex (five‐ and six‐class) alternatives. The classification quality was acceptable, with high class‐wise posterior membership probabilities and entropy indicating adequate separation, thereby supporting reliable categorization without evidence of overfitting.

In the older adult cohort, Trajectory 3 (stabilized elevated ICP) was more prevalent (28.67%, Table S6B) than in the general population (12.06%, Table S2B, at the 120‐h window). Cox regression analyses (Table S7) confirmed that Trajectories 3 and 4 were consistently associated with increased mortality, similar to the findings in the overall cohort. Notably, older adults exhibited a higher hazard ratio (HR) for Trajectory 3 (HR = 2.87) than that of the overall population (HR = 2.53). These results were consistent across the MIMIC‐IV and eICU databases. Due to the limited sample size, the NSICU estimates should be interpreted cautiously.

### 3.5 Enhanced Mortality Prediction by Incorporating Four ICP Trajectories

Following the identification of four distinct ICP trajectories and their association with in‐hospital mortality, we assessed whether incorporating these trajectories into predictive models could enhance mortality risk prediction. Feature selection across the merged dataset identified 13 key variables (Figures S9–S11 and Table S8).

The Trajectory Model outperformed the Baseline Model in terms of AUC across the merged dataset, achieving 0.80 (95% CI: 0.77–0.82) compared with 0.76 (95% CI: 0.74–0.79) for the Baseline Model (*p* < 0.001). In the NSICU cohort, the Trajectory Model showed a higher AUC of 0.95 (95% CI: 0.92–0.99) compared with 0.92 (95% CI: 0.85–0.99) for the Baseline Model, although the difference was not statistically significant (*p* = 0.13). The Trajectory Model also demonstrated significant improvements in IDI and NRI across most cohorts. The merged dataset showed improvements in the IDI of 0.065 (*p* < 0.001), NRI of 0.201 (*p* < 0.001), and median improvement in risk score of 0.03 (*p* < 0.001), indicating better discrimination and reclassification of patient outcomes. The IDI was significant in both the MIMIC‐IV and eICU cohorts, whereas NRI was significant in the MIMIC‐IV and NSICU but not in the eICU (*p* = 0.08). These ROC findings are shown in Figure S12, and the summary statistics are reported in Table 3. Patient‐level reclassification is shown in Figure S13, which displays the distribution of individual risk changes after adding ICP trajectories. Gains were most evident in the merged and MIMIC‐IV cohorts, with smaller/nonsignificant shifts in the eICU and NSICU cohorts. Model interpretability was summarized using SHAP plots (Figure S14).

**TABLE 3:** Performance of mortality prediction models across cohorts.

Model	AUC		IDI		NRI		Median improvement in risk score	
	HR (95% CI)	*p*	Estimate (95% CI)	*p*	Estimate (95% CI)	*p*	Estimate (95% CI)	*p*
**Merged data**								
Baseline	0.76 (0.74–0.79)							
Trajectory	0.80 (0.77–0.82)	< 0.001	0.065 (0.038–0.091)	< 0.001	0.201 (0.139–0.266)	< 0.001	0.03 (0.014–0.049)	< 0.001
**MIMIC‐IV**								
Baseline	0.78 (0.74–0.82)							
Trajectory	0.81 (0.77–0.84)	0.01	0.063 (0.029–0.096)	< 0.001	0.196 (0.099–0.269)	0.001	0.017 (0.002–0.05)	0.02
**eICU**								
Baseline	0.77 (0.73–0.81)							
Trajectory	0.80 (0.76–0.83)	0.01	0.039 (0.013–0.075)	< 0.001	0.144 (−0.011–0.254)	0.08	0.009 (0–0.024)	0.02
**NSICU**								
Baseline	0.92 (0.85–0.99)							
Trajectory	0.95 (0.92–0.99)	0.13	0.042 (−0.028–0.113)	0.24	0.57 (0.13–1.012)	0.01	NA	NA

*Note:* Comparison of a Baseline Model and a Trajectory Model (Baseline + ICP trajectories). The Baseline Model included Age, Heart Rate, Temperature, Urine Output, BUN, WBC, Hypertension, Initial GCS, Intubation, Vasopressor, Dialysis, Mannitol, and Embolization. Model improvement was assessed using AUC, IDI, and NRI. NA (not applicable) for the NSICU cohort due to limited sample size.

## 4 Discussion

We identified four clinically interpretable intracranial pressure (ICP) trajectories across three ICU datasets, aligning time zero with monitoring initiation and evaluating 72‐, 120‐, and 168‐h windows. In the primary 120‐h analysis, a severe‐progressive pattern carried the greatest mortality risk, followed by stabilized‐elevated and mildly elevated patterns, whereas a low‐and‐stable pattern was the most favorable. Among older patients (≥ 55 years), the stabilized‐elevated pattern appeared particularly deleterious, underscoring that temporal dynamics (persistence and progression) convey prognostic information beyond single thresholds or static averages. Adding trajectory membership to a baseline clinical model improved risk stratification in the merged cohort and in most external datasets. In the smallest center, gains in discrimination were less certain, although reclassification improved. Overall, trajectory‐based characterization provides incremental clinical value for outcome prediction in patients with ABI.

ICP monitoring plays a pivotal role in neurocritical care, particularly in patients with ABI, such as TBI, ICH, and ischaemic stroke [3, 5, 18]. Elevated ICP impairs cerebral perfusion, leading to secondary ischaemic injury and, if untreated, potentially fatal herniation [4, 19]. Clinicians rely on ICP monitoring to detect rising pressure early and guide interventions, such as osmotherapy or surgical decompression, to maintain CPP and prevent further brain damage. Despite its critical importance, ICP monitoring remains invasive, typically requiring intraparenchymal probes or external ventricular drains, each carrying inherent risks such as infection, hemorrhage, and device‐related complications [6, 20]. These risks have fuelled an ongoing debate regarding the necessity of routine ICP monitoring, especially in patients with mild to moderate brain injuries or in resource‐limited settings [21, 22]. Moreover, the clinical utility of fixed ICP thresholds, such as 20 and 22 mmHg, has been increasingly questioned because these static thresholds fail to capture individual patient variability [23]. The lack of universally accepted guidelines has exacerbated this debate. While routine monitoring is widely recommended for severe TBI, some guidelines suggest that it may not be necessary for less severe injuries [3, 5, 20]. This inconsistency reflects uncertainty in clinical decision‐making and is further influenced by institutional policies that determine whether ICP monitoring is necessary for specific patient populations [22].

Our study addresses the limitations of fixed ICP thresholds by demonstrating that dynamic ICP monitoring provides superior mortality risk prediction [24, 25]. By capturing temporal ICP fluctuations, our dynamic modeling approach enables a more nuanced and accurate assessment of mortality risk compared with traditional static thresholds. Finally, the consistency of these findings across multiple cohorts (MIMIC‐IV, eICU, and NSICU) strengthened the robustness and applicability of our models. In addition to previous reports [5, 8], we externally validated a parsimonious four‐class trajectory model across three cohorts and time windows, demonstrated age‐specific risk amplification (notably for a stabilized‐elevated pattern), quantified incremental prognostic value using IDI/cNRI and patient‐level risk shifts, and provided clinician‐facing interpretability via SHAP analyses linking predictions to actionable covariates.

Traditional static ICP monitoring has limitations in capturing dynamic fluctuations, complicating ABI management [23, 26]. To address this limitation, we employed LCGM to provide a detailed and individualized analysis of ICP trajectories [7, 27]. Unlike static monitoring, which relies on fixed thresholds, LCGM captures temporal ICP variability, enabling the identification of distinct progression patterns that reflect the evolving nature of brain injury [9, 28]. Originally developed for sepsis, this dynamic modeling approach has been successfully adapted for neurocritical care, enhancing risk stratification and enabling personalized outcome predictions [12, 29, 30]. We analyzed data from 1700 patients with ABI across the MIMIC‐IV, eICU, and NSICU databases, using three observation windows (72, 120, and 168 h) starting from the first valid ICP measurement. This methodology enhances statistical power and temporal resolution, allowing for more precise tracking of ICP fluctuations, which are essential for capturing patient‐specific dynamics. The clinical relevance of the identified ICP trajectories is underscored by their strong correlations with patient outcomes.

Each ICP trajectory represents a distinct dynamic pattern that influences cerebral perfusion and mortality risk. Trajectory 1 (Normal ICP) reflects stable intracranial homeostasis, characterized by minimal ICP fluctuations below 15 mmHg, intact cerebral autoregulation, and favorable clinical outcomes. Trajectory 2 (Mildly Elevated ICP) displays mild but stable elevations (15–25 mmHg), commonly observed after mild‐to‐moderate brain injury, preserves cerebral perfusion, and is usually managed conservatively. Trajectory 3 (Stabilized Elevated ICP) features persistently elevated ICP (~26 mmHg) that stabilizes without normalizing, indicative of partial autoregulatory impairment. These patients, particularly older adults, require intensive monitoring due to a higher mortality risk. Trajectory 4 (Severe Progressive ICP) represents severe and progressively worsening intracranial hypertension, often exceeding 50 mmHg, accompanied by significant variability, severely compromised cerebral perfusion, and the highest mortality risk, necessitating aggressive and individualized interventions.

Trajectory‐based dynamic modeling improves mortality risk stratification, yielding consistent results across merged and individual cohorts (MIMIC‐IV, eICU, and NSICU). Our model outperformed the Baseline Model in terms of AUC, IDI, and NRI, providing a more accurate and personalized approach to patient care beyond the traditional static thresholds (e.g., the 20‐mmHg cutoff). This enhanced predictive accuracy aligns with prior research on dynamic monitoring [8, 17]. This simplified four‐class classification supports bedside interpretability in time‐pressured ICU settings. Unlike studies that focused on cerebrovascular reactivity indices, such as the pressure reactivity index (PRx) [31, 32], our model directly associates temporal ICP patterns with tissue‐level ischaemic burden. By capturing intra‐patient variability, short‐term fluctuations, and long‐term tendencies, the model yields clinically interpretable signals that can inform tailored interventions. Although the absolute AUC gain was modest, decision‐oriented metrics (IDI/NRI) indicated a net correct reclassification around the plausible action thresholds. In practice, a sustained trajectory 3/4 pattern can prompt earlier reassessment or escalation within a CPP‐guided strategy, and trajectories are intended to complement (not replace) standard ICP/CPP targets.

Subgroup analysis revealed a significant interaction between age and ICP trajectories, highlighting the distinct ICP dynamics in older adults (≥ 55 years). Factors such as reduced cerebrovascular compliance, brain atrophy, and age‐related comorbidities contributed to more complex ICP patterns in this cohort [33, 34]. Consistent with this interaction and contributing factors, we applied the four‐class ICP trajectory model to older adults, confirming its applicability in this subgroup. The model effectively captured greater ICP variability in older adults across multiple observation windows (72, 120, and 168 h), underscoring its clinical relevance in neurocritical care. Specifically, older adults with stabilized elevated ICP (trajectory 3) exhibited a significantly higher mortality risk (HR = 2.87) than those with the same trajectory in the overall cohort (HR = 2.53), indicating that elevated ICP carries an increased risk in older patients. Clinically, these findings support age‐aware trajectory management: when an older adult shows a sustained Trajectory 3/4 pattern despite first‐line care, clinicians should shorten reassessment intervals and consider earlier escalation (e.g., optimization of osmotherapy and CSF diversion, where appropriate) with multimodal, CPP‐guided monitoring; goals should be individualized by frailty and comorbidity. We do not propose new fixed ICP cutoffs; rather, trajectories should trigger a timely review of etiology and treatment intensity in older adults. Given the increased variability and heightened mortality risk in this cohort, integrating age‐specific dynamic models into clinical practice is essential for optimizing ICP management and improving patient outcomes [8, 33, 35]. Although ICP pathophysiology varies across TBI, ICH, and ischemic stroke, our prespecified analyses of traumatic versus nontraumatic cases revealed no significant effect modification. A consistent stepwise risk gradient was observed within both strata, supporting the pooled ABI analysis for class discovery.

## 5 Limitations

This study has several limitations. First, as a retrospective analysis, the study was subject to missing data and unmeasured confounding because time zero was monitoring initiation rather than ICU admission. Trajectories describing post‐initiation dynamics and pre‐monitoring variability, with potential lead‐time/immortal‐time bias, cannot be excluded. Because physiological covariates were summarized from the first 24 h, they reflect early severity rather than subsequent clinical evolution, and residual confounding by later physiology may remain. Prospective studies with standardized monitoring initiation and the use of time‐updated covariates (e.g., joint/dynamic models) could mitigate these concerns while avoiding overadjustment for mediators in the ICP–outcome pathway. Second, although ICU discharge GCS scores and in‐hospital mortality provide valuable insights, they may not accurately reflect long‐term neurological outcomes. The lack of Glasgow Outcome Scale (GOS) scores at 3 months is a key limitation [32]. Third, the absence of neuroimaging data, such as CT scans and cerebral angiography, limited the ability to assess the impact of structural brain injuries and vascular conditions on ICP trajectories. Finally, the extended study period introduced variability in ICP monitoring, including catheter use and treatment protocols, which likely evolved over time. Future research should explicitly disentangle the contributions of ICP trajectories versus age‐related comorbidity, ideally through prospective designs (e.g., target‐trial emulation or randomized studies) with granular comorbidity and multimodal monitoring to further enhance the clinical relevance and predictive accuracy of ICP trajectory modeling. Additionally, these trajectory models should be validated for different etiologies (e.g., traumatic vs. nontraumatic) to assess their generalizability and accuracy across diverse clinical contexts.

## 6 Conclusion

In this multicentre cohort, four ICP trajectory classes showed graded associations with in‐hospital mortality. Adding trajectory membership to a baseline clinical model provided incremental prognostic value beyond static summaries. Stabilized‐elevated and severe‐progressive patterns were consistently associated with a higher risk relative to the low‐and‐stable pattern, with signals particularly pronounced in older patients (≥ 55 years). Trajectory information may support more individualized monitoring and treatment prioritization. However, prospective multicentre studies with standardized measurement protocols are needed to validate these classes, enable real‐time implementation, and determine whether trajectory‐guided management improves outcomes.

## Author Contributions

Juan Wang and Hai‐bo Li contributed equally to the study conception and design, data acquisition, statistical analysis, and writing of the manuscript. Man‐Man Xu, Wen‐Juan Li, Long‐Yang Cheng, and Shao‐Ya Li were involved in the analysis and interpretation of the data and critical revision of the manuscript. Peng‐Lai Zhao and Chun‐Hua Hang supervised the study and approved the final manuscript. All the authors have reviewed and approved the submitted version of the manuscript.

## Funding

This work was supported by funding for clinical trials from the Affiliated Drum Tower Hospital, Medical School, Nanjing University (No. LCYJ‐MS‐37, No. LCYJ‐PY‐38), Beijing Medical Award Foundation (YXJL‐2022‐0351‐0421), Beijing Bethune Charitable Foundation (2022‐YJ‐085‐J‐Z‐ZZ‐026), and Nanjing Drum Tower Hospital Clinical Research Fund Project (2024‐LCYJ‐MS‐08).

## Ethics Statement

This retrospective study was approved by the Research Ethics Committee of Drum Tower Hospital (approval no. 2023‐565‐03). All datasets used (MIMIC‐IV, eICU, and NSICU) were de‐identified and anonymized to ensure patient privacy. As this study involved a retrospective analysis of anonymized patient data, the requirement for informed consent was waived by the ethics committee.

## Consent

The authors have nothing to report.

## Conflicts of Interest

The authors declare no conflicts of interest.

## Supporting information


**Data S1:** cns70735‐sup‐0001‐Supinfo.docx.

## Data Availability

The data used in this study can be accessed via the MIMIC‐IV database (https://physionet.org/content/mimiciv/3.1/) and the eICU database (https://physionet.org/content/eicu‐crd/2.0/); full access instructions are available on the respective website. The NSICU dataset generated and analyzed in the current study is available from the corresponding author upon reasonable request.
