# Supplementary material for: Four Distinct Dynamic Intracranial Pressure Trajectories and Their Prognostic Implications in Acute Brain Injury: A Multicenter Cohort Study
Source: CNS Neurosci Ther. 2026 Jan 3;32(1):e70735. doi: 10.1002/cns.70735 (PMC12759305; doi:10.1002/cns.70735)
Supplement: Supplementary file 1 — Data S1: cns70735‐sup‐0001‐Supinfo.docx. [file CNS-32-e70735-s001.docx]

# Supplementary Figure 1. Patient Cohort Selection Flowchart


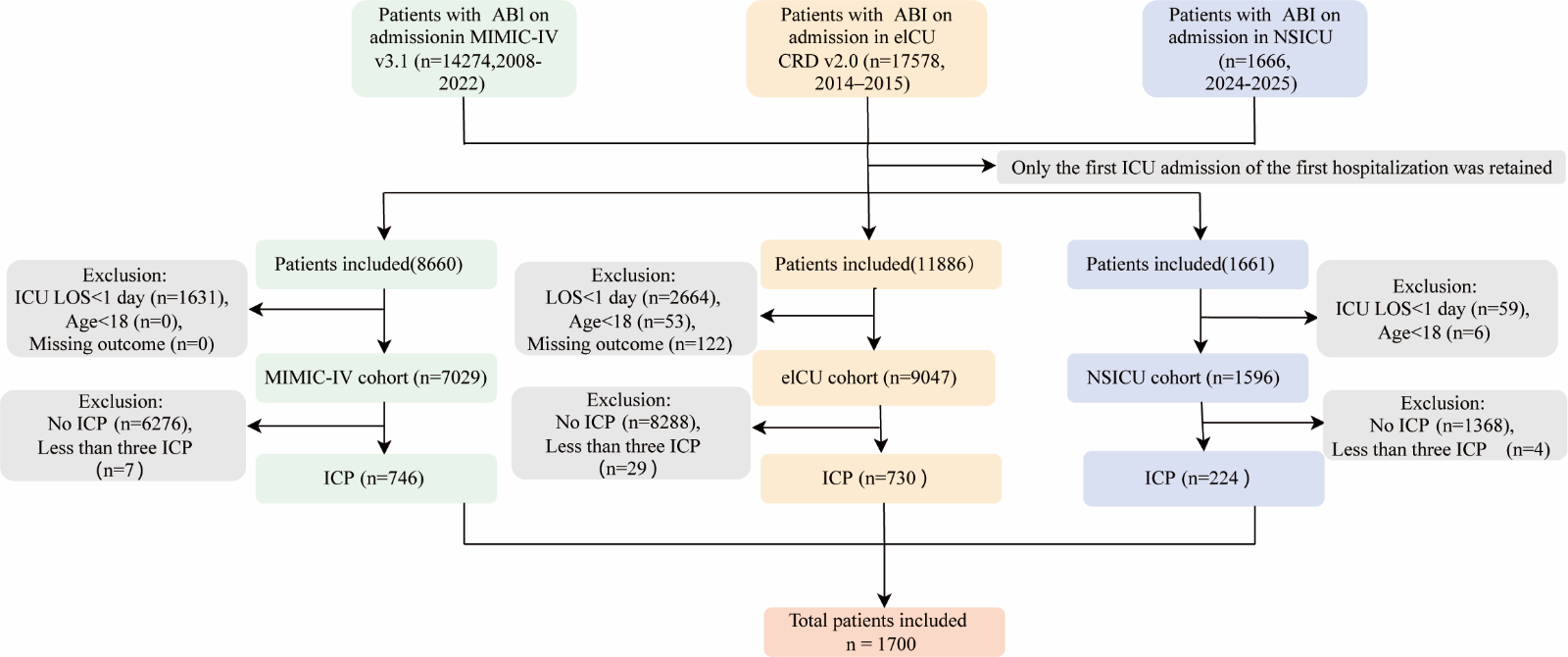


**Legend:** ABI admissions were identified in MIMIC-IV v3.1, eICU-CRD v2.0, and NSICU; analyses used the index ICU stay (first ICU admission during the first hospitalization). Exclusions were ICU length of stay <1 day, age <18 years, missing in-hospital outcome, and no ICP monitoring or fewer than three ICP measurements. Final sample: n=1,700 (MIMIC-IV n=746; eICU n=730; NSICU n=224). **Abbreviations:** ABI, acute brain injury; ICU, intensive care unit; LOS, length of stay; ICP, intracranial pressure; MIMIC-IV, Medical Information Mart for Intensive Care IV; eICU-CRD, eICU Collaborative Research Database; NSICU, Neurosurgical Intensive Care Unit.

# Supplementary Table 1. Clinical Variables Extracted for Analysis

| **Category** | **Variables** |
| --- | --- |
| **Demographic** | Age (years); Sex (0=female, 1=male); BMI (kg/m²) |
| **Admission** | Admission type (0=elective, 1=emergency); Admission time (0=day shift, 1=night shift); Traumatic etiology (0=no, 1=yes); Initial ICP (mmHg); Initial CPP (mmHg); Initial GCS (score) |
| **Physiological Parameters** | Respiratory rate (breaths/min); Heart rate (beats/min); MBP (mmHg); Temperature (°C); Mean ICP (mmHg); Final ICP (mmHg); Mean CPP (mmHg); Final CPP (mmHg); Mean GCS (score); Final GCS (score) |
| **Laboratory Variables** | Urine output (mL; the first ICU day); ALT (U/L); BUN (mg/dL); Creatinine (mg/dL); Platelet count (PLT, ×10⁹/L); RBC (×10¹²/L); WBC (×10⁹/L) |
| **Comorbidities** | Hypertension (0=no, 1=yes); Diabetes (0=no, 1=yes); Liver disease (0=no, 1=yes); Stroke history (0=no, 1=yes) |
| **Interventions** | Intubation (0=no, 1=yes); Vasopressor (0=no, 1=yes; first ICU day); Dialysis (0=no, 1=yes); Mannitol (0=no, 1=yes; first ICU day); Craniotomy (0=no, 1=yes); Embolization (0=no, 1=yes) |
| **Discharge Location** | Acute/ICU care (0=no, 1=yes); Chronic/Long-term care (0=no, 1=yes); Home care/Recovery (0=no, 1=yes) |
| **Length of Stay & Outcome** | Length of hospital stay (days); Length of ICU stay (days); In-hospital mortality (0=no, 1=yes) |

**Notes:** All continuous variables were assessed for distributional shape using the Shapiro–Wilk test together with visual diagnostics (Q–Q plots and histograms). Given the large sample size, formal tests frequently indicated non-normality despite approximately symmetric appearances. Accordingly, between-trajectory comparisons for continuous variables were conducted primarily with Kruskal–Wallis tests (Dunn post-hoc with Holm adjustment); one-way ANOVA with Tukey’s HSD was considered only where distributions and variances were reasonably well-behaved and yielded concordant inferences. For descriptive presentation, most continuous variables are reported as mean ± SD to aid clinical interpretability, whereas clearly right-skewed variables are summarized as median (IQR) (e.g., BMI, urine output, ALT, creatinine). Categorical variables are n (%) and were compared using χ² or Fisher’s exact tests, as appropriate. No transformations were applied for baseline display. Comorbidities were identified from diagnosis codes in each database (ICD-9/10 for MIMIC-IV and eICU-CRD; local EHR codes for NSICU). **Abbreviations:** BMI, body mass index; MBP, mean blood pressure; ICP, intracranial pressure; CPP, cerebral perfusion pressure; GCS, Glasgow Coma Scale; ALT, alanine aminotransferase; BUN, blood urea nitrogen; PLT, platelet count; RBC, red blood cell count; WBC, white blood cell count; bpm, breaths/beats per minute.

# Supplementary Table 2A. Model Fit Statistics for 72-Hour ICP Trajectory Models in the Integrated Dataset

| **G** | **Log-Likelihood** | **AIC** | **BIC** | **SABIC** | **Entropy** | **Class Proportion (%)** | | | | | | **Average Posterior Probability** | | | | | |
| --- | --- | --- | --- | --- | --- | --- | --- | --- | --- | --- | --- | --- | --- | --- | --- | --- | --- |
|  |  |  |  |  |  | **1** | **2** | **3** | **4** | **5** | **6** | **1** | **2** | **3** | **4** | **5** | **6** |
| 2 | -81736.30 | 163502.60 | 163584.18 | 163536.52 | 0.67 | 80.71 | 19.29 |  |  |  |  | 0.93 | 0.87 |  |  |  |  |
| 3 | -81502.69 | 163045.38 | 163154.15 | 163090.61 | 0.71 | 43.41 | 9.94 | 46.65 |  |  |  | 0.88 | 0.90 | 0.87 |  |  |  |
| 4 | -81454.18 | 162958.37 | 163094.32 | 163014.90 | 0.68 | 37.82 | 18.00 | 36.53 | 7.65 |  |  | 0.86 | 0.68 | 0.83 | 0.91 |  |  |
| 5 | -81436.23 | 162932.45 | 163095.61 | 163000.30 | 0.61 | 32.06 | 17.94 | 16.76 | 6.59 | 26.65 |  | 0.81 | 0.64 | 0.61 | 0.89 | 0.76 |  |
| 6 | -81446.24 | 162962.48 | 163152.83 | 163041.64 | 0.60 | 32.76 | 15.94 | 26.06 | 15.18 | 3.76 | 6.29 | 0.80 | 0.60 | 0.65 | 0.75 | 0.86 | 0.66 |

**Notes:** Model fit statistics for the 72-hour ICP LCGM in the integrated dataset. The optimal number of classes (G) was selected based on the model with the lowest Bayesian Information Criterion (BIC) and sample-size adjusted BIC (SABIC), while also ensuring high entropy and meaningful class proportions. Lower values for AIC, BIC, and SABIC indicate a better relative model fit. The average posterior probabilities reflect the certainty of classification for each class.

# Supplementary Table 2B. Model Fit Statistics for 120-Hour ICP Trajectory Models in the Integrated Dataset

| **G** | **Log-Likelihood** | **AIC** | **BIC** | **SABIC** | **Entropy** | **Class Proportion (%)** | | | | | | **Average Posterior Probability** | | | | | |
| --- | --- | --- | --- | --- | --- | --- | --- | --- | --- | --- | --- | --- | --- | --- | --- | --- | --- |
|  |  |  |  |  |  | **1** | **2** | **3** | **4** | **5** | **6** | **1** | **2** | **3** | **4** | **5** | **6** |
| 2 | -148871.30 | 297772.61 | 297854.18 | 297806.53 | 0.79 | 88.00 | 12.00 |  |  |  |  | 0.96 | 0.88 |  |  |  |  |
| 3 | -148616.62 | 297273.25 | 297382.01 | 297318.48 | 0.73 | 43.71 | 7.59 | 48.71 |  |  |  | 0.87 | 0.93 | 0.89 |  |  |  |
| 4 | -148576.08 | 297202.17 | 297338.13 | 297258.70 | 0.68 | 41.29 | 42.53 | 12.06 | 4.12 |  |  | 0.86 | 0.79 | 0.78 | 0.89 |  |  |
| 5 | -148531.61 | 297123.22 | 297286.37 | 297191.06 | 0.69 | 34.06 | 23.76 | 34.94 | 1.29 | 5.94 |  | 0.83 | 0.67 | 0.83 | 0.80 | 0.88 |  |
| 6 | -148530.47 | 297130.95 | 297321.29 | 297210.10 | 0.62 | 18.35 | 21.18 | 27.35 | 25.12 | 1.29 | 6.71 | 0.76 | 0.65 | 0.65 | 0.76 | 0.82 | 0.85 |

# Supplementary Table 2C. Model Fit Statistics for 168-Hour ICP Trajectory Models in the Integrated Dataset

| **G** | **Log-Likelihood** | **AIC** | **BIC** | **SABIC** | **Entropy** | **Class Proportion (%)** | | | | | | **Average Posterior Probability** | | | | | |
| --- | --- | --- | --- | --- | --- | --- | --- | --- | --- | --- | --- | --- | --- | --- | --- | --- | --- |
|  |  |  |  |  |  | **1** | **2** | **3** | **4** | **5** | **6** | **1** | **2** | **3** | **4** | **5** | **6** |
| 2 | -99929.84 | 199889.69 | 199971.26 | 199923.61 | 0.61 | 52.59 | 47.41 |  |  |  |  | 0.86 | 0.92 |  |  |  |  |
| 3 | -99652.72 | 199345.43 | 199454.20 | 199390.66 | 0.71 | 42.18 | 10.29 | 47.53 |  |  |  | 0.88 | 0.89 | 0.87 |  |  |  |
| 4 | -99612.51 | 199275.03 | 199410.99 | 199331.57 | 0.68 | 41.47 | 41.06 | 12.59 | 4.88 |  |  | 0.87 | 0.79 | 0.75 | 0.82 |  |  |
| 5 | -99593.21 | 199246.42 | 199409.58 | 199314.27 | 0.63 | 26.35 | 16.76 | 42.12 | 3.24 | 11.53 |  | 0.74 | 0.64 | 0.80 | 0.82 | 0.78 |  |
| 6 | -99591.91 | 199253.82 | 199444.16 | 199332.97 | 0.57 | 22.82 | 21.06 | 12.82 | 30.88 | 2.06 | 10.35 | 0.71 | 0.59 | 0.58 | 0.67 | 0.81 | 0.77 |

# Supplementary Table 2D. Model Fit Statistics for 72-Hour ICP Trajectory Models in the MIMIC-IV Dataset

| **G** | **Log-Likelihood** | **AIC** | **BIC** | **SABIC** | **Entropy** | **Class Proportion (%)** | | | | | | **Average Posterior Probability** | | | | | |
| --- | --- | --- | --- | --- | --- | --- | --- | --- | --- | --- | --- | --- | --- | --- | --- | --- | --- |
|  |  |  |  |  |  | **1** | **2** | **3** | **4** | **5** | **6** | **1** | **2** | **3** | **4** | **5** | **6** |
| 2 | -33266.21 | 66562.42 | 66631.64 | 66584.01 | 0.74 | 73.46 | 26.54 |  |  |  |  | 0.94 | 0.92 |  |  |  |  |
| 3 | -33147.62 | 66335.23 | 66427.53 | 66364.02 | 0.78 | 63.14 | 4.02 | 32.84 |  |  |  | 0.91 | 0.96 | 0.90 |  |  |  |
| 4 | -33122.13 | 66294.26 | 66409.63 | 66330.25 | 0.68 | 37.94 | 32.04 | 25.87 | 4.16 |  |  | 0.80 | 0.74 | 0.89 | 0.94 |  |  |
| 5 | -33101.89 | 66263.78 | 66402.22 | 66306.96 | 0.73 | 37.40 | 28.42 | 22.25 | 4.16 | 7.77 |  | 0.82 | 0.75 | 0.86 | 0.97 | 0.84 |  |
| 6 | -33099.14 | 66268.28 | 66429.80 | 66318.66 | 0.67 | 37.13 | 21.98 | 18.23 | 13.00 | 3.08 | 6.57 | 0.81 | 0.67 | 0.72 | 0.72 | 0.95 | 0.83 |

# Supplementary Table 2E. Model Fit Statistics for 120-Hour ICP Trajectory Models in the MIMIC-IV Dataset

| **G** | **Log-Likelihood** | **AIC** | **BIC** | **SABIC** | **Entropy** | **Class Proportion (%)** | | | | | | **Average Posterior Probability** | | | | | |
| --- | --- | --- | --- | --- | --- | --- | --- | --- | --- | --- | --- | --- | --- | --- | --- | --- | --- |
|  |  |  |  |  |  | **1** | **2** | **3** | **4** | **5** | **6** | **1** | **2** | **3** | **4** | **5** | **6** |
| 2 | -59799.83 | 119629.66 | 119698.88 | 119651.25 | 0.78 | 82.04 | 17.96 |  |  |  |  | 0.95 | 0.90 |  |  |  |  |
| 3 | -59641.13 | 119322.26 | 119414.56 | 119351.05 | 0.77 | 65.28 | 4.29 | 30.43 |  |  |  | 0.91 | 0.95 | 0.89 |  |  |  |
| 4 | -59583.64 | 119217.27 | 119332.64 | 119253.26 | 0.81 | 64.88 | 30.83 | 2.95 | 1.34 |  |  | 0.90 | 0.89 | 0.95 | 0.94 |  |  |
| 5 | -59566.31 | 119192.62 | 119331.07 | 119235.80 | 0.68 | 37.27 | 36.33 | 22.12 | 1.34 | 2.95 |  | 0.75 | 0.74 | 0.85 | 0.92 | 0.94 |  |
| 6 | -59556.97 | 119183.93 | 119345.45 | 119234.31 | 0.68 | 38.34 | 29.09 | 6.43 | 21.85 | 1.34 | 2.95 | 0.79 | 0.69 | 0.63 | 0.87 | 0.96 | 0.91 |

# Supplementary Table 2F. Model Fit Statistics for 168-Hour ICP Trajectory Models in the MIMIC-IV Dataset

| **G** | **Log-Likelihood** | **AIC** | **BIC** | **SABIC** | **Entropy** | **Class Proportion (%)** | | | | | | **Average Posterior Probability** | | | | | |
| --- | --- | --- | --- | --- | --- | --- | --- | --- | --- | --- | --- | --- | --- | --- | --- | --- | --- |
|  |  |  |  |  |  | **1** | **2** | **3** | **4** | **5** | **6** | **1** | **2** | **3** | **4** | **5** | **6** |
| 2 | -40840.02 | 81710.05 | 81779.27 | 81731.64 | 0.75 | 76.41 | 23.59 |  |  |  |  | 0.94 | 0.93 |  |  |  |  |
| 3 | -40701.72 | 81443.43 | 81535.73 | 81472.22 | 0.78 | 66.09 | 5.50 | 28.42 |  |  |  | 0.91 | 0.94 | 0.89 |  |  |  |
| 4 | -40683.73 | 81417.46 | 81532.83 | 81453.44 | 0.64 | 27.48 | 41.82 | 25.47 | 5.23 |  |  | 0.72 | 0.75 | 0.87 | 0.95 |  |  |
| 5 | -40671.58 | 81403.17 | 81541.61 | 81446.35 | 0.62 | 38.74 | 17.16 | 16.09 | 5.23 | 22.79 |  | 0.75 | 0.72 | 0.61 | 0.94 | 0.83 |  |
| 6 | -40657.04 | 81384.09 | 81545.60 | 81434.47 | 0.65 | 32.04 | 29.22 | 12.47 | 10.72 | 5.36 | 10.19 | 0.75 | 0.71 | 0.63 | 0.71 | 0.94 | 0.85 |

# Supplementary Table 2G. Model Fit Statistics for 72-Hour ICP Trajectory Models in the eICU Dataset

| **G** | **Log-Likelihood** | **AIC** | **BIC** | **SABIC** | **Entropy** | **Class Proportion (%)** | | | | | | **Average Posterior Probability** | | | | | |
| --- | --- | --- | --- | --- | --- | --- | --- | --- | --- | --- | --- | --- | --- | --- | --- | --- | --- |
|  |  |  |  |  |  | **1** | **2** | **3** | **4** | **5** | **6** | **1** | **2** | **3** | **4** | **5** | **6** |
| 2 | -36245.85 | 72521.70 | 72590.60 | 72542.97 | 0.66 | 85.34 | 14.66 |  |  |  |  | 0.92 | 0.88 |  |  |  |  |
| 3 | -36218.20 | 72476.39 | 72568.25 | 72504.75 | 0.59 | 25.07 | 60.82 | 14.11 |  |  |  | 0.77 | 0.81 | 0.87 |  |  |  |
| 4 | -36203.44 | 72456.88 | 72571.71 | 72492.33 | 0.64 | 62.05 | 11.37 | 21.64 | 4.93 |  |  | 0.82 | 0.73 | 0.79 | 0.86 |  |  |
| 5 | -36190.95 | 72441.90 | 72579.69 | 72484.43 | 0.57 | 22.19 | 11.78 | 22.33 | 4.79 | 38.90 |  | 0.76 | 0.72 | 0.79 | 0.87 | 0.66 |  |
| 6 | -36195.95 | 72461.91 | 72622.67 | 72511.53 | 0.48 | 24.38 | 11.51 | 37.95 | 21.92 | 3.70 | 0.55 | 0.70 | 0.64 | 0.58 | 0.64 | 0.87 | 0.40 |

# Supplementary Table 2H. Model Fit Statistics for 120-Hour ICP Trajectory Models in the eICU Dataset

| **G** | **Log-Likelihood** | **AIC** | **BIC** | **SABIC** | **Entropy** | **Class Proportion (%)** | | | | | | **Average Posterior Probability** | | | | | |
| --- | --- | --- | --- | --- | --- | --- | --- | --- | --- | --- | --- | --- | --- | --- | --- | --- | --- |
|  |  |  |  |  |  | **1** | **2** | **3** | **4** | **5** | **6** | **1** | **2** | **3** | **4** | **5** | **6** |
| 2 | -66826.36 | 133682.72 | 133751.61 | 133703.99 | 0.76 | 89.59 | 10.41 |  |  |  |  | 0.95 | 0.91 |  |  |  |  |
| 3 | -66802.90 | 133645.80 | 133737.66 | 133674.15 | 0.59 | 30.41 | 59.59 | 10.00 |  |  |  | 0.74 | 0.82 | 0.91 |  |  |  |
| 4 | -66783.05 | 133616.09 | 133730.92 | 133651.53 | 0.62 | 24.66 | 60.55 | 12.33 | 2.47 |  |  | 0.73 | 0.79 | 0.80 | 0.86 |  |  |
| 5 | -66777.99 | 133615.99 | 133753.78 | 133658.52 | 0.62 | 23.01 | 60.14 | 2.74 | 2.74 | 11.37 |  | 0.72 | 0.77 | 0.72 | 0.90 | 0.81 |  |
| 6 | -66772.59 | 133615.17 | 133775.93 | 133664.79 | 0.64 | 22.05 | 61.10 | 1.51 | 2.19 | 2.33 | 10.82 | 0.70 | 0.76 | 0.81 | 0.71 | 0.80 | 0.78 |

# Supplementary Table 2I. Model Fit Statistics for 168-Hour ICP Trajectory Models in the eICU Dataset

| **G** | **Log-Likelihood** | **AIC** | **BIC** | **SABIC** | **Entropy** | **Class Proportion (%)** | | | | | | **Average Posterior Probability** | | | | | |
| --- | --- | --- | --- | --- | --- | --- | --- | --- | --- | --- | --- | --- | --- | --- | --- | --- | --- |
|  |  |  |  |  |  | **1** | **2** | **3** | **4** | **5** | **6** | **1** | **2** | **3** | **4** | **5** | **6** |
| 2 | -44598.52 | 89227.04 | 89295.94 | 89248.31 | 0.62 | 82.74 | 17.26 |  |  |  |  | 0.91 | 0.85 |  |  |  |  |
| 3 | -44576.07 | 89192.15 | 89284.01 | 89220.50 | 0.54 | 66.30 | 5.89 | 27.81 |  |  |  | 0.80 | 0.81 | 0.75 |  |  |  |
| 4 | -44563.26 | 89176.52 | 89291.34 | 89211.96 | 0.56 | 11.78 | 53.56 | 29.73 | 4.93 |  |  | 0.72 | 0.72 | 0.78 | 0.80 |  |  |
| 5 | -44555.04 | 89170.09 | 89307.88 | 89212.62 | 0.54 | 54.25 | 13.70 | 25.21 | 6.16 | 0.68 |  | 0.72 | 0.60 | 0.70 | 0.77 | 0.83 |  |
| 6 | -44560.24 | 89190.48 | 89351.23 | 89240.10 | 0.52 | 13.42 | 57.26 | 3.56 | 23.01 | 2.47 | 0.27 | 0.70 | 0.68 | 0.60 | 0.64 | 0.87 | 0.43 |

# Supplementary Table 2J. Model Fit Statistics for 72-Hour ICP Trajectory Models in the NSICU Dataset

| **G** | **Log-Likelihood** | **AIC** | **BIC** | **SABIC** | **Entropy** | **Class Proportion (%)** | | | | | | **Average Posterior Probability** | | | | | |
| --- | --- | --- | --- | --- | --- | --- | --- | --- | --- | --- | --- | --- | --- | --- | --- | --- | --- |
|  |  |  |  |  |  | **1** | **2** | **3** | **4** | **5** | **6** | **1** | **2** | **3** | **4** | **5** | **6** |
| 2 | -10835.77 | 21701.54 | 21752.71 | 21705.17 | 0.84 | 81.25 | 18.75 |  |  |  |  | 0.96 | 0.98 |  |  |  |  |
| 3 | -10826.57 | 21693.14 | 21761.37 | 21697.99 | 0.84 | 79.46 | 11.16 | 9.38 |  |  |  | 0.96 | 0.94 | 0.79 |  |  |  |
| 4 | -10818.84 | 21687.68 | 21772.97 | 21693.74 | 0.87 | 81.25 | 10.27 | 5.36 | 3.13 |  |  | 0.96 | 0.84 | 0.85 | 0.93 |  |  |
| 5 | -10810.84 | 21681.68 | 21784.03 | 21688.95 | 0.77 | 48.66 | 24.55 | 8.93 | 14.73 | 3.13 |  | 0.89 | 0.79 | 0.80 | 0.92 | 0.94 |  |
| 6 | -10812.58 | 21695.16 | 21814.56 | 21703.64 | 0.80 | 1.79 | 70.98 | 5.36 | 10.27 | 7.14 | 4.46 | 0.84 | 0.92 | 0.68 | 0.72 | 0.88 | 0.78 |

# Supplementary Table 2K. Model Fit Statistics for 120-Hour ICP Trajectory Models in the NSICU Dataset

| **G** | **Log-Likelihood** | **AIC** | **BIC** | **SABIC** | **Entropy** | **Class Proportion (%)** | | | | | | **Average Posterior Probability** | | | | | |
| --- | --- | --- | --- | --- | --- | --- | --- | --- | --- | --- | --- | --- | --- | --- | --- | --- | --- |
|  |  |  |  |  |  | **1** | **2** | **3** | **4** | **5** | **6** | **1** | **2** | **3** | **4** | **5** | **6** |
| 2 | -19376.56 | 38783.12 | 38834.30 | 38786.76 | 0.86 | 80.80 | 19.20 |  |  |  |  | 0.96 | 0.98 |  |  |  |  |
| 3 | -19360.99 | 38761.98 | 38830.21 | 38766.83 | 0.74 | 71.88 | 11.16 | 16.96 |  |  |  | 0.92 | 0.94 | 0.80 |  |  |  |
| 4 | -19355.84 | 38761.69 | 38846.98 | 38767.75 | 0.77 | 72.32 | 16.52 | 8.93 | 2.23 |  |  | 0.92 | 0.78 | 0.87 | 0.82 |  |  |
| 5 | -19346.84 | 38753.68 | 38856.02 | 38760.95 | 0.75 | 43.75 | 37.50 | 4.02 | 12.50 | 2.23 |  | 0.83 | 0.80 | 0.82 | 0.89 | 0.92 |  |
| 6 | -19341.88 | 38753.76 | 38873.17 | 38762.25 | 0.76 | 52.68 | 26.34 | 3.57 | 4.46 | 10.71 | 2.23 | 0.86 | 0.76 | 0.82 | 0.82 | 0.93 | 0.87 |

# Supplementary Table 2L. Model Fit Statistics for 168-Hour ICP Trajectory Models in the NSICU Dataset

| **G** | **Log-Likelihood** | **AIC** | **BIC** | **SABIC** | **Entropy** | **Class Proportion (%)** | | | | | | **Average Posterior Probability** | | | | | |
| --- | --- | --- | --- | --- | --- | --- | --- | --- | --- | --- | --- | --- | --- | --- | --- | --- | --- |
|  |  |  |  |  |  | **1** | **2** | **3** | **4** | **5** | **6** | **1** | **2** | **3** | **4** | **5** | **6** |
| 2 | -12610.75 | 25251.51 | 25302.68 | 25255.14 | 0.80 | 75.89 | 24.11 |  |  |  |  | 0.95 | 0.95 |  |  |  |  |
| 3 | -12590.65 | 25221.30 | 25289.53 | 25226.14 | 0.78 | 68.75 | 6.70 | 24.55 |  |  |  | 0.93 | 0.92 | 0.86 |  |  |  |
| 4 | -12578.01 | 25206.01 | 25291.31 | 25212.08 | 0.67 | 31.70 | 40.63 | 21.43 | 6.25 |  |  | 0.78 | 0.77 | 0.85 | 0.93 |  |  |
| 5 | -12572.40 | 25204.80 | 25307.15 | 25212.08 | 0.68 | 33.04 | 29.91 | 7.14 | 6.70 | 23.21 |  | 0.79 | 0.73 | 0.74 | 0.93 | 0.85 |  |
| 6 | -12573.41 | 25216.81 | 25336.22 | 25225.30 | 0.64 | 29.02 | 30.80 | 7.14 | 4.46 | 6.70 | 21.88 | 0.76 | 0.65 | 0.74 | 0.58 | 0.94 | 0.82 |

# Supplementary Figure 2. Distribution of ICP Trajectory Classes Across Cohorts and Time Windows


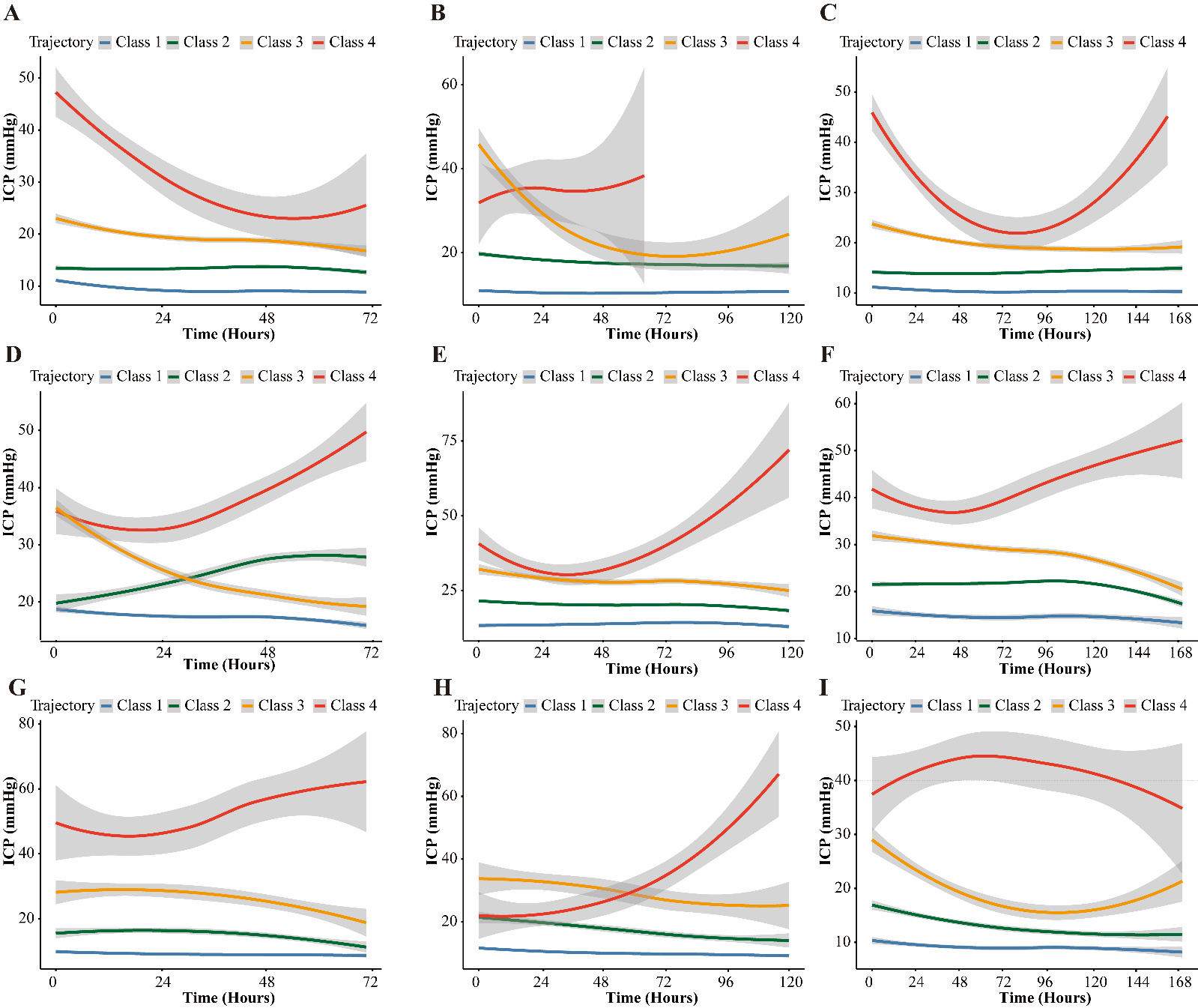


**Legend:** Panels A–C show MIMIC-IV; D–F, eICU; G–I, NSICU. Within each dataset, panels correspond to 72 h (5-h intervals), 120 h (4-h), and 168 h (8-h). Curves show class-specific mean ICP with 95% CIs (shaded bands). Trajectory definitions and fixed color mapping: Trajectory 1—low and stable (blue); Trajectory 2—mildly elevated, stable (green); Trajectory 3—elevated with a stabilizing plateau (yellow); Trajectory 4—high and progressively rising with greater variability (red).

# Supplementary Table 3A. Baseline Characteristics by ICP Trajectory in the MIMIC-IV Dataset

| **Characteristic** | **Total  (n = 746)** | **Trajectory 1  (n = 484)** | **Trajectory 2 (n = 230)** | **Trajectory 3  (n = 10)** | **Trajectory 4 (n = 22)** | ***P*** |
| --- | --- | --- | --- | --- | --- | --- |
| **Age, years** | 59.1 ± 17.4 | 64.1 ± 15.1 | 49.8 ± 17.4 | 47.7 ± 18.8 | 55.1 ± 19.2 | < 0.001 |
| **Sex, Male** | 391 (52.4) | 232 (47.9) | 141 (61.3) | 13 (59.1) | 5 (50) | 0.005 |
| **BMI** | 27.9 ± 6.4 | 27.7 ± 6.7 | 28.2 ± 5.8 | 26.9 ± 5.2 | 26.7 ± 6.2 | 0.755 |
| **Respiratory Rate, bpm** | 19.2 ± 3.3 | 18.9 ± 3.1 | 19.5 ± 3.6 | 21.5 ± 3.0 | 22.2 ± 5.2 | < 0.001 |
| **Heart Rate, bpm** | 81.0 ± 14.0 | 79.8 ± 12.7 | 82.4 ± 15.3 | 90.7 ± 20.5 | 87.3 ± 10.9 | < 0.001 |
| **MBP, mmHg** | 83.5 ± 8.3 | 83.2 ± 8.4 | 83.8 ± 8.2 | 86.4 ± 6.5 | 87.2 ± 7.7 | 0.123 |
| **Temperature, ℃** | 37.1 ± 0.5 | 37.1 ± 0.4 | 37.2 ± 0.6 | 37.1 ± 0.8 | 36.8 ± 1.2 | 0.27 |
| **Urine Output, ml** | 2370.0 ± 1433.2 | 2131.8 ± 1260.6 | 2608.5 ± 1343.1 | 3902.3 ± 2286.5 | 4995.8 ± 2929.6 | < 0.001 |
| **ALT, U/L** | 25.0 (17.0, 41.0) | 22.0 (16.0, 30.0) | 34.0 (19.0, 72.0) | 54.0 (23.0, 109.0) | 35.0 (20.0, 68.5) | < 0.001 |
| **BUN, mg/dL** | 18.3 ± 9.9 | 18.6 ± 9.6 | 17.5 ± 10.3 | 20.7 ± 11.2 | 16.2 ± 6.2 | 0.325 |
| **Creatinine, mg/dL** | 0.9 (0.7, 1.1) | 0.9 (0.7, 1.1) | 1.0 (0.8, 1.2) | 1.1 (0.9, 1.8) | 0.8 (0.8, 1.0) | 0.001 |
| **PLT, ×10^9/L** | 201.8 ± 81.5 | 208.4 ± 84.0 | 190.4 ± 73.8 | 182.2 ± 88.3 | 186.8 ± 91.9 | 0.027 |
| **RBC, 10¹²/L** | 3.7 ± 0.9 | 3.8 ± 0.9 | 3.6 ± 1.0 | 3.3 ± 1.2 | 3.9 ± 0.8 | 0.018 |
| **WBC, 10⁹/L** | 15.8 ± 7.0 | 15.1 ± 7.0 | 16.6 ± 6.1 | 22.1 ± 10.2 | 17.4 ± 8.8 | < 0.001 |
| **Admission Type，Emergency** | 679 (91.0) | 439 (90.7) | 210 (91.3) | 20 (90.9) | 10 (100) | 0.967 |
| **Admission Time** |  |  |  |  |  | 0.059 |
| **Day Shift** | 545 (73.1) | 345 (71.3) | 176 (76.5) | 14 (63.6) | 10 (100) |  |
| **Night Shift** | 201 (26.9) | 139 (28.7) | 54 (23.5) | 8 (36.4) | 0 (0) |  |
| **Traumatic** | 123 (16.5) | 35 (7.2) | 78 (33.9) | 8 (36.4) | 2 (20) | < 0.001 |
| **Hypertension** | 451 (60.5) | 331 (68.4) | 106 (46.1) | 10 (45.5) | 4 (40) | < 0.001 |
| **Diabetes** | 119 (16.0) | 82 (16.9) | 33 (14.3) | 2 (9.1) | 2 (20) | 0.674 |
| **Liver Disease** | 28 (3.8) | 20 (4.1) | 7 (3) | 0 (0) | 1 (10) | 0.428 |
| **Stroke History** | 28 (3.8) | 19 (3.9) | 7 (3) | 1 (4.5) | 1 (10) | 0.376 |
| **Initial GCS** | 8 ± 4 | 8 ±4 | 8 ± 4 | 8 ± 6 | 12 ± 5 | 0.017 |
| **Intubation** | 598 (80.2) | 379 (78.3) | 190 (82.6) | 20 (90.9) | 9 (90) | 0.308 |
| **Vasopressor** | 196 (26.3) | 95 (19.6) | 81 (35.2) | 14 (63.6) | 6 (60) | < 0.001 |
| **Dialysis** | 20 (2.7) | 11 (2.3) | 8 (3.5) | 1 (4.5) | 0 (0) | 0.508 |
| **Mannitol** | 143 (19.2) | 59 (12.2) | 68 (29.6) | 10 (45.5) | 6 (60) | < 0.001 |
| **Craniotomy** | 500 (67.0) | 313 (64.7) | 165 (71.7) | 15 (68.2) | 7 (70) | 0.286 |
| **Embolization** | 272 (36.5) | 200 (41.3) | 65 (28.3) | 5 (22.7) | 2 (20) | 0.004 |

**Notes:** Missing data: Variables with <10% missing data include BUN, Creatinine, Initial GCS, Heart Rate, MBP, PLT, Respiratory Rate, Temperature, WBC, and Urine Output. BMI is the only variable with 20–30% missing data. ALT is the only variable with more than 50% missing data. The variables Urine Output, Vasopressor, and Mannitol specifically refer to values recorded on Day 1 of ICU admission. Abbreviations: ICP, intracranial pressure; BMI, body mass index; bpm, breaths/beats per minute; MBP, mean blood pressure; ALT, alanine aminotransferase; BUN, blood urea nitrogen; PLT, platelet; RBC, red blood cell; WBC, white blood cell; GCS, Glasgow Coma Scale (initial assessment upon admission).

# Supplementary Table 3B. Baseline Characteristics by ICP Trajectory in the eICU Dataset

| **Characteristic** | **Total  (n = 730)** | **Trajectory 1  (n = 180)** | **Trajectory 2 (n = 442)** | **Trajectory 3  (n = 90)** | **Trajectory 4**  **(n = 18)** | ***P*** |
| --- | --- | --- | --- | --- | --- | --- |
| **Age, years** | 54.3 ± 17.2 | 59.0 ± 14.7 | 53.0 ± 17.7 | 51.7 ± 17.6 | 50.4 ± 18.3 | < 0.001 |
| **Sex, Male** | 416 (57.0) | 103 (57.2) | 246 (55.7) | 59 (65.6) | 8 (44.4) | 0.243 |
| **BMI** | 27.2 (23.9, 31.6) | 26.6 (23.2, 31.8) | 27.4 (24.0, 31.3) | 27.4 (24.4, 31.4) | 27.4 (22.3, 34.0) | 0.94 |
| **Respiratory Rate, bpm** | 23.3 ± 13.8 | 21.6 ± 12.7 | 23.7 ± 13.7 | 22.4 ± 15.5 | 33.8 ± 13.6 | 0.003 |
| **Heart Rate, bpm** | 106.7 ± 31.3 | 105.6 ± 31.7 | 107.5 ± 30.8 | 105.8 ± 32.3 | 105.1 ± 34.3 | 0.886 |
| **MBP, mmHg** | 103.1 ± 44.2 | 104.2 ± 42.3 | 101.4 ± 44.1 | 106.4 ± 46.8 | 118.4 ± 49.9 | 0.33 |
| **Temperature, ℃** | 36.4 ± 1.2 | 36.4 ± 1.0 | 36.5 ± 1.2 | 36.3 ± 1.6 | 36.1 ± 1.1 | 0.324 |
| **Urine Output, ml** | 1990.4 (1186.2, 3263.8) | 1983.9 (1212.3, 3029.7) | 1983.7 (1168.5, 3122.7) | 2351.3 (1529.2, 3864.9) | 1581.6 (813.5, 3045.4) | 0.237 |
| **ALT, U/L** | 29.0 (18.0, 48.0) | 29.0 (19.2, 49.2) | 30.0 (18.0, 48.0) | 24.0 (17.5, 37.5) | 37.0 (26.0, 127.0) | 0.147 |
| **BUN, mg/dL** | 17.1 ± 9.5 | 17.4 ± 9.4 | 17.0 ± 9.2 | 17.3 ± 11.6 | 16.3 ± 7.4 | 0.93 |
| **Creatinine, mg/dL** | 0.9 (0.7, 1.2) | 0.9 (0.7, 1.1) | 0.9 (0.7, 1.2) | 0.9 (0.8, 1.2) | 1.0 (0.8, 1.2) | 0.247 |
| **PLT, ×10^9/L** | 200.0 ± 72.7 | 203.4 ± 78.1 | 198.8 ± 71.6 | 200.3 ± 70.6 | 193.6 ± 56.6 | 0.89 |
| **RBC, 10¹²/L** | 3.9 ± 0.8 | 4.0 ± 0.8 | 3.9 ± 0.7 | 3.8 ± 0.7 | 3.8 ± 0.7 | 0.241 |
| **WBC, 10⁹/L** | 15.5 ± 6.4 | 15.3 ± 6.3 | 15.5 ± 6.6 | 15.7 ± 5.7 | 17.1 ± 5.8 | 0.708 |
| **Admission Type，Emergency** | 412 (56.4) | 99 (55) | 251 (56.8) | 50 (55.6) | 12 (66.7) | 0.809 |
| **Admission Time** |  |  |  |  |  | 0.565 |
| **Day Shift** | 417 (57.1) | 107 (59.4) | 244 (55.2) | 56 (62.2) | 10 (55.6) |  |
| **Night Shift** | 313 (42.9) | 73 (40.6) | 198 (44.8) | 34 (37.8) | 8 (44.4) |  |
| **Traumatic** | 296 (40.5) | 66 (36.7) | 181 (41) | 41 (45.6) | 8 (44.4) | 0.531 |
| **Hypertension** | 300 (41.1) | 84 (46.7) | 174 (39.4) | 34 (37.8) | 8 (44.4) | 0.341 |
| **Diabetes** | 79 (10.8) | 18 (10) | 47 (10.6) | 12 (13.3) | 2 (11.1) | 0.822 |
| **Liver Disease** | 6 (0.8) | 0 (0) | 6 (1.4) | 0 (0) | 0 (0) | 0.35 |
| **Stroke History** | 55 (7.5) | 21 (11.7) | 28 (6.3) | 6 (6.7) | 0 (0) | 0.089 |
| **Initial GCS** | 8.4 ± 4.4 | 8.3 ± 4.3 | 8.4 ± 4.4 | 8.5 ± 4.7 | 7.5 ± 4.1 | 0.877 |
| **Intubation** | 342 (46.8) | 73 (40.6) | 214 (48.4) | 49 (54.4) | 6 (33.3) | 0.082 |
| **Vasopressor** | 183 (25.1) | 43 (23.9) | 104 (23.5) | 30 (33.3) | 6 (33.3) | 0.195 |
| **Dialysis** | 8 (1.1) | 2 (1.1) | 4 (0.9) | 2 (2.2) | 0 (0) | 0.536 |
| **Mannitol** | 93 (12.7) | 22 (12.2) | 50 (11.3) | 15 (16.7) | 6 (33.3) | 0.029 |
| **Craniotomy** | 379 (51.9) | 102 (56.7) | 233 (52.7) | 37 (41.1) | 7 (38.9) | 0.067 |
| **Embolization** | 141 (19.3) | 35 (19.4) | 89 (20.1) | 15 (16.7) | 2 (11.1) | 0.797 |

**Notes:** Missing data: Variables with <10% missing data include BMI, BUN, Creatinine, Heart Rate, MBP, PLT, RBC, Respiratory Rate, Temperature, and WBC. Initial GCS is the only variable with 10–20% missing data. Variables with 30–50% missing data include ALT and Urine Output.

# Supplementary Table 3C. Baseline Characteristics by ICP Trajectory in the NSICU Dataset

| **Characteristic** | **Total**  **(n = 224)** | **Trajectory 1**  **(n = 162)** | **Trajectory 2**  **(n = 37)** | **Trajectory 3**  **(n = 20)** | **Trajectory 4**  **(n = 5)** | ***P*** |
| --- | --- | --- | --- | --- | --- | --- |
| **Age, years** | 57.3 ± 14.6 | 58.8 ± 13.8 | 54.1 ± 15.2 | 53.5 ± 17.5 | 47.6 ± 17.4 | 0.072 |
| **Sex, Male** | 126 (56.2) | 95 (58.6) | 22 (59.5) | 8 (40) | 1 (20) | 0.156 |
| **BMI** | 24.6 ± 6.0 | 25.0 ± 6.5 | 24.6 ± 4.7 | 22.4 ± 3.6 | 21.9 ± 4.4 | 0.272 |
| **Respiratory Rate, bpm** | 17.4 ± 6.2 | 17.0 ± 2.9 | 17.3 ± 3.0 | 21.1 ± 18.7 | 16.6 ± 1.3 | 0.053 |
| **Heart Rate, bpm** | 84.8 ± 12.6 | 83.9 ± 12.2 | 88.8 ± 12.8 | 84.6 ± 15.8 | 85.8 ± 5.8 | 0.212 |
| **MBP, mmHg** | 141.3 ± 18.7 | 141.8 ± 18.0 | 144.3 ± 20.7 | 132.3 ± 20.5 | 137.8 ± 9.3 | 0.117 |
| **Temperature, ℃** | 36.7 ± 0.5 | 36.7 ± 0.5 | 36.6 ± 0.6 | 36.8 ± 0.5 | 36.3 ± 0.4 | 0.181 |
| **Urine Output, ml** | 1990.4  (1186.2, 3263.8) | 1983.9 (1212.3, 3029.7) | 1983.7 (1168.5, 3122.7) | 2351.3 (1529.2, 3864.9) | 1581.6 (813.5, 3045.4) | 0.237 |
| **ALT, U/L** | 29.0  (18.0, 48.0) | 29.0  (19.2, 49.2) | 30.0  (18.0, 48.0) | 24.0  (17.5, 37.5) | 37.0  (26.0, 127.0) | 0.147 |
| **BUN, mg/dL** | 17.1 ± 9.5 | 17.4 ± 9.4 | 17.0 ± 9.2 | 17.3 ± 11.6 | 16.3 ± 7.4 | 0.93 |
| **Creatinine, mg/dL** | 0.9 (0.7, 1.2) | 0.9 (0.7, 1.1) | 0.9 (0.7, 1.2) | 0.9 (0.8, 1.2) | 1.0 (0.8, 1.2) | 0.247 |
| **PLT, ×10^9/L** | 167.4 ± 61.3 | 166.2 ± 61.7 | 170.9 ± 52.5 | 175.7 ± 71.5 | 145.8 ± 79.8 | 0.763 |
| **RBC, 10¹²/L** | 3.7 ± 0.6 | 3.7 ± 0.6 | 3.9 ± 0.6 | 3.4 ± 1.0 | 3.4 ± 0.4 | 0.023 |
| **WBC, 10⁹/L** | 11.4 ± 4.0 | 11.0 ± 3.7 | 11.8 ± 2.9 | 13.8 ± 5.5 | 11.8 ± 7.7 | 0.021 |
| **Admission Type，Emergency** | 222 (99.1) | 160 (98.8) | 37 (100) | 20 (100) | 5 (100) | 1 |
| **Admission Time** |  |  |  |  |  | 0.848 |
| **Day Shift** | 148 (66.7) | 109 (68.1) | 23 (62.2) | 13 (65) | 3 (60) |  |
| **Night Shift** | 74 (33.3) | 51 (31.9) | 14 (37.8) | 7 (35) | 2 (40) |  |
| **Traumatic** | 63 (28.1) | 40 (24.7) | 12 (32.4) | 10 (50) | 1 (20) | 0.101 |
| **Hypertension** | 137 (61.2) | 98 (60.5) | 26 (70.3) | 10 (50) | 3 (60) | 0.479 |
| **Diabetes** | 26 (11.6) | 19 (11.7) | 4 (10.8) | 2 (10) | 1 (20) | 0.8 |
| **Liver Disease** | 43 (19.2) | 35 (21.6) | 5 (13.5) | 3 (15) | 0 (0) | 0.597 |
| **Stroke History** | 74 (33.0) | 58 (35.8) | 8 (21.6) | 5 (25) | 3 (60) | 0.162 |
| **Initial GCS** | 3.0 (3.0, 3.0) | 3.0 (3.0, 3.0) | 3.0 (3.0, 3.0) | 3.0 (3.0, 3.0) | 3.0 (3.0, 3.0) | 0.482 |
| **Intubation** | 128 (57.1) | 92 (56.8) | 22 (59.5) | 11 (55) | 3 (60) | 0.986 |
| **Vasopressor** | 16 (7.1) | 9 (5.6) | 2 (5.4) | 5 (25) | 0 (0) | 0.038 |
| **Dialysis** | 2 (0.9) | 0 (0) | 0 (0) | 0 (0) | 2 (40) | < 0.001 |
| **Mannitol** | 112 (50.0) | 83 (51.2) | 19 (51.4) | 7 (35) | 3 (60) | 0.551 |
| **Craniotomy** | 172 (76.8) | 128 (79) | 26 (70.3) | 14 (70) | 4 (80) | 0.517 |
| **Embolization** | 24 (10.7) | 19 (11.7) | 4 (10.8) | 0 (0) | 1 (20) | 0.315 |

**Notes:** Missing data: BUN is the only variable with 10–20% missing data, while all other covariates with missing data have less than 10% missing.

# Supplementary Figure 3. GCS by ICP Trajectory Across Cohorts


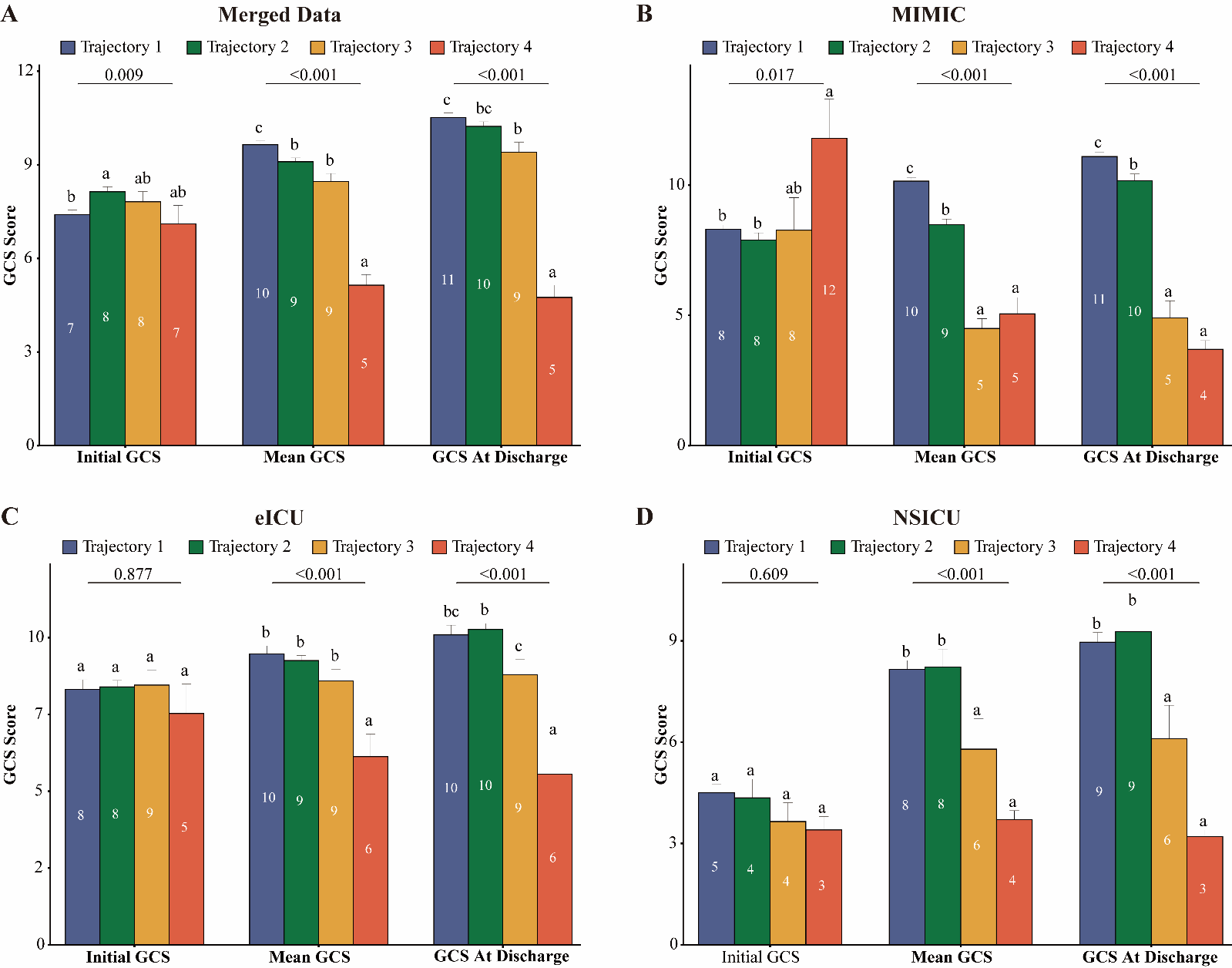


**Legend:** Comparison of GCS scores across the four ICP trajectories at admission (Initial GCS), during ICU stay (Mean GCS), and at discharge. The analysis is presented for the merged dataset **(A)** and for the individual MIMIC-IV **(B)**, eICU **(C)**, and NSICU **(D)** cohorts. Bars represent mean GCS. P-values indicate the significance of the overall comparison across the four trajectories at each time point. Letters (a, b, c) above the bars denote results from post-hoc pairwise comparisons; trajectories sharing a letter are not significantly different from each other.

# Supplementary Figure **4.** CPP by ICP Trajectory Across Cohorts


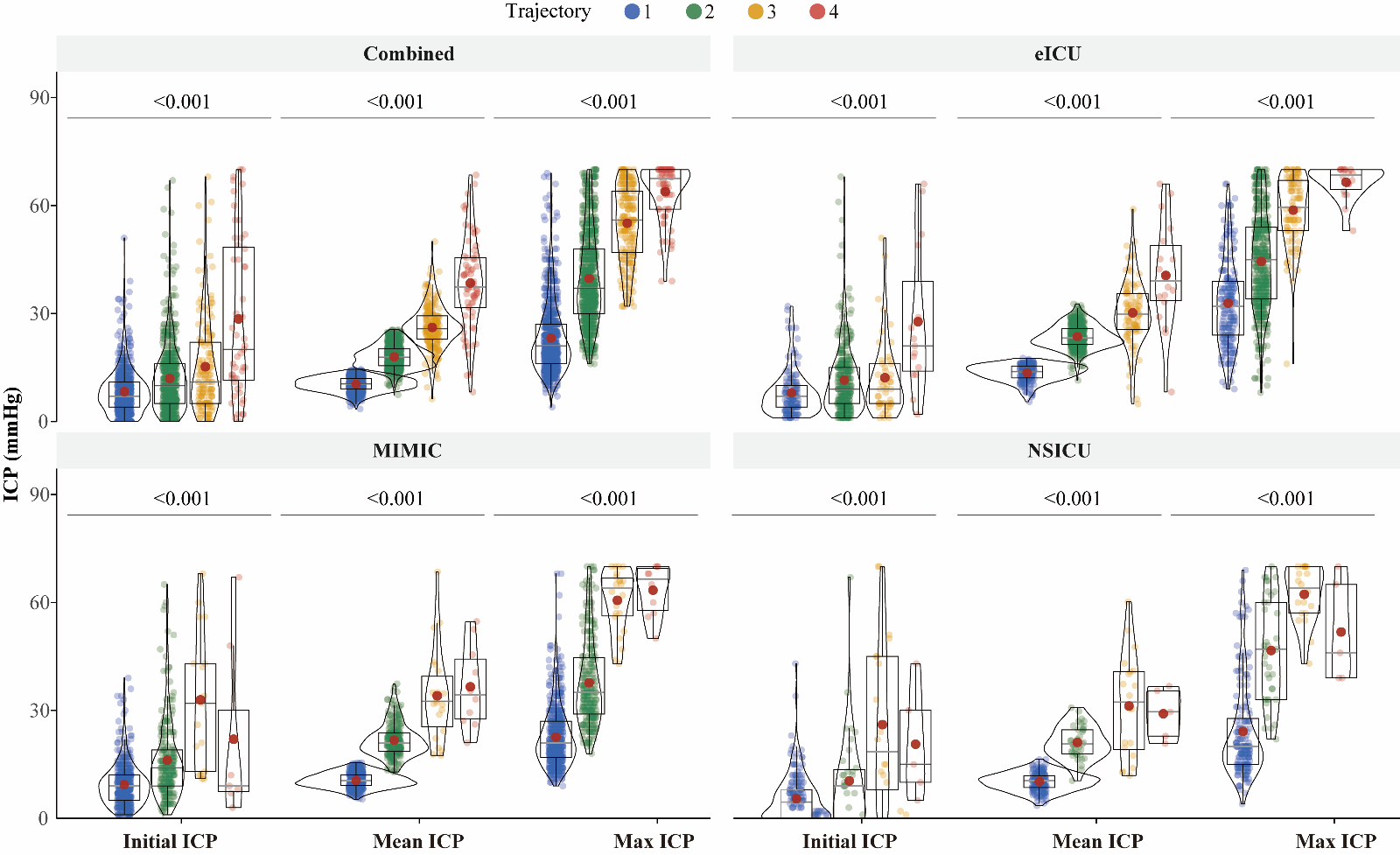


**Legend:** Distributions of initial, mean, and maximum ICP are compared across the four ICP trajectories. The analysis is shown for the combined dataset and the individual MIMIC, eICU, and NSICU cohorts. Violin plots depict the data distribution, with embedded boxplots indicating the median and IQR. Red dots represent the mean. P-values shown above each group indicate the significance of the overall comparison across the four trajectories.

# Supplementary Figure 5. ICP Levels by Trajectory Across Cohorts


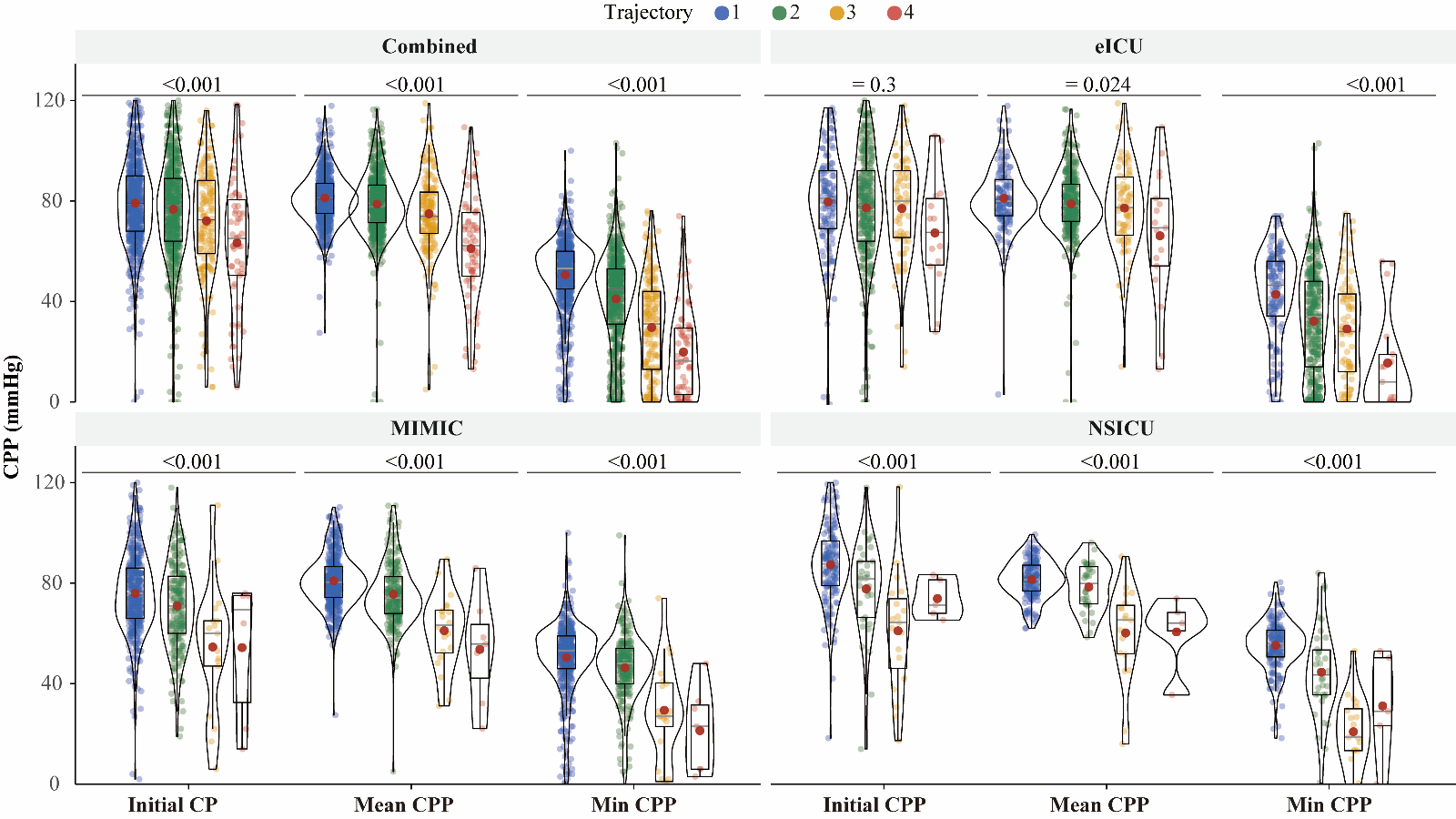


**Legend:** Distributions of initial, mean, and minimum CPP are compared across the four ICP trajectories. The analysis is shown for the combined dataset and the individual MIMIC, eICU, and NSICU cohorts.

# Supplementary Figure 6. Discharge Destination by ICP Trajectory Across Cohorts


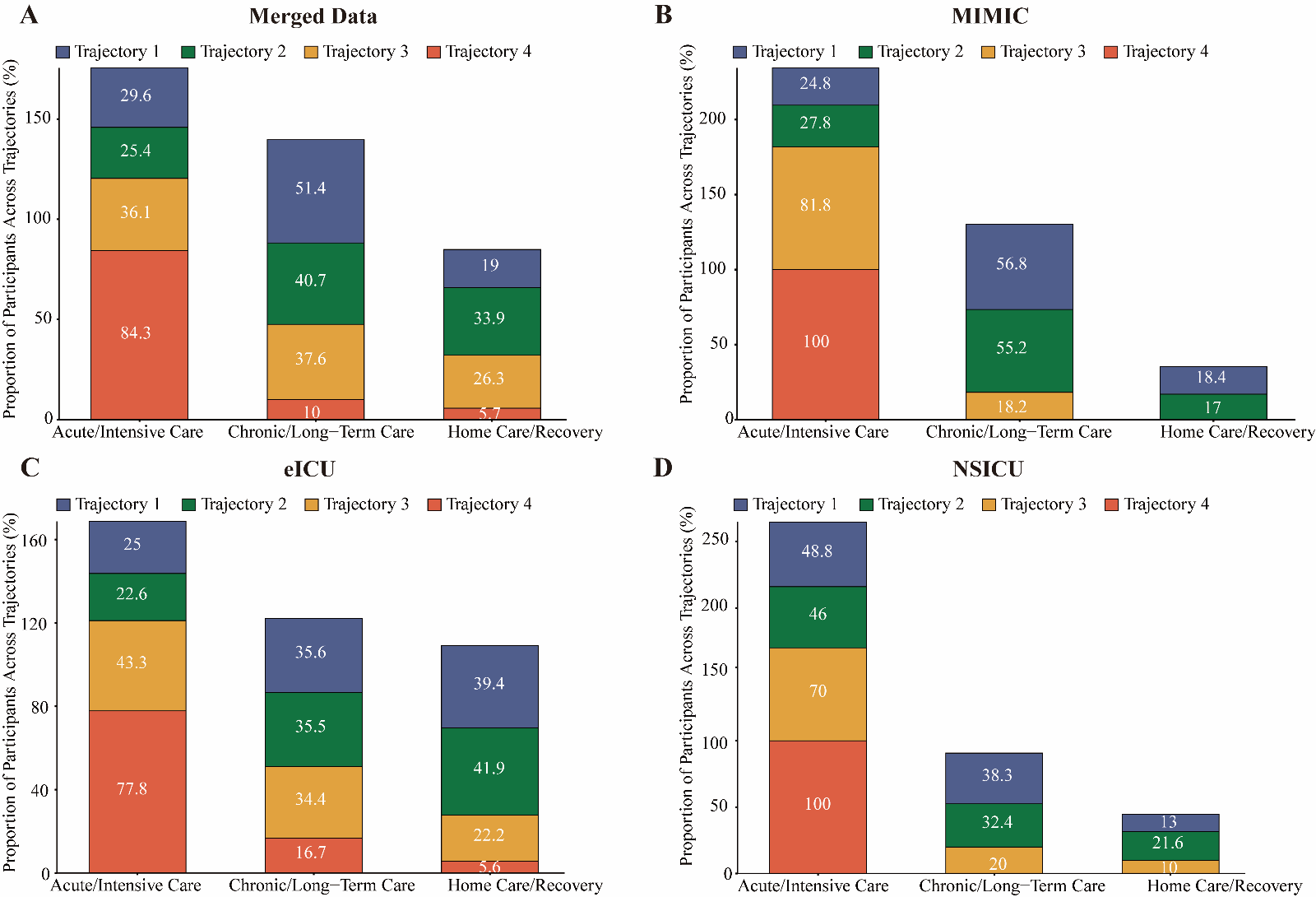


**Legend:** Distribution of discharge destinations according to the four ICP trajectories. The analysis is shown for the merged dataset **(A)** and for the individual MIMIC **(B)**, eICU **(C)**, and NSICU **(D)** cohorts. The numeric labels specify the within-trajectory percentage for each destination; for any given trajectory (color), the percentages across the three destination categories sum to 100%.

# Supplementary Table 4. Selection of Covariates for Multivariable Regression Models

| **Crude Model** | | | **Full Model** | | | **VIF** | **Collinearity** | **Select** | **Select VIF** |
| --- | --- | --- | --- | --- | --- | --- | --- | --- | --- |
| **Term1** | **Coeff1** | **Change1 (%)** | **Term2** | **Coeff2** | **Change2 (%)** |  |  |  |  |
| Crude for Trajectory = 2 | 0.14 | Ref. | Full for Trajectory = 2 | 0.4 | Ref. | 1.677 | 0 | Ref. | Ref. |
| Crude for Trajectory = 3 | 0.61 | Ref. | Full for Trajectory = 3 | 0.99 | Ref. | 1.677 | 0 | Ref. | Ref. |
| Crude for Trajectory = 4 | 2.12 | Ref. | Full for Trajectory = 4 | 2.64 | Ref. | 1.677 | 0 | Ref. | Ref. |
| Age for Trajectory = 2 | 0.41 | 181.2 | Age for Trajectory = 2 | 0.22 | -45 | 1.632 | 0 | Yes | Yes |
| Age for Trajectory = 3 | 0.92 | 49.8 | Age for Trajectory = 3 | 0.75 | -24.1 | 1.632 | 0 | Yes | Yes |
| Age for Trajectory = 4 | 2.44 | 15 | Age for Trajectory = 4 | 2.47 | -6.5 | 1.632 | 0 | Yes | Yes |
| Sex for Trajectory = 2 | 0.15 | 0.3 | Sex for Trajectory = 2 | 0.4 | -1.1 | 1.191 | 0 | No | No |
| Sex for Trajectory = 3 | 0.61 | 0.1 | Sex for Trajectory = 3 | 0.98 | -1 | 1.191 | 0 | No | No |
| Sex for Trajectory = 4 | 2.12 | 0 | Sex for Trajectory = 4 | 2.63 | -0.5 | 1.191 | 0 | No | No |
| BMI for Trajectory = 2 | 0.14 | -4.2 | BMI for Trajectory = 2 | 0.41 | 1.6 | 1.093 | 0 | No | No |
| BMI for Trajectory = 3 | 0.61 | 0 | BMI for Trajectory = 3 | 0.99 | -0.1 | 1.093 | 0 | No | No |
| BMI for Trajectory = 4 | 2.12 | 0 | BMI for Trajectory = 4 | 2.64 | 0 | 1.093 | 0 | No | No |
| Respiratory Rate for Trajectory = 2 | 0.14 | -4.2 | Respiratory Rate for Trajectory = 2 | 0.4 | -0.4 | 1.217 | 0 | No | No |
| Respiratory Rate for Trajectory = 3 | 0.61 | -0.8 | Respiratory Rate for Trajectory = 3 | 0.99 | -0.1 | 1.217 | 0 | No | No |
| Respiratory Rate for Trajectory = 4 | 2.1 | -0.8 | Respiratory Rate for Trajectory = 4 | 2.63 | -0.3 | 1.217 | 0 | No | No |
| Heart Rate for Trajectory = 2 | 0.13 | -12.8 | Heart Rate for Trajectory = 2 | 0.43 | 6.2 | 1.503 | 0 | Yes | Yes |
| Heart Rate for Trajectory = 3 | 0.59 | -3.7 | Heart Rate for Trajectory = 3 | 1 | 0.9 | 1.503 | 0 | No | No |
| Heart Rate for Trajectory = 4 | 2.1 | -1 | Heart Rate for Trajectory = 4 | 2.69 | 1.7 | 1.503 | 0 | No | No |
| MBP for Trajectory = 2 | 0.15 | 5.8 | MBP for Trajectory = 2 | 0.4 | -1.5 | 1.255 | 0 | No | No |
| MBP for Trajectory = 3 | 0.65 | 5.6 | MBP for Trajectory = 3 | 0.97 | -1.9 | 1.255 | 0 | No | No |
| MBP for Trajectory = 4 | 2.27 | 7.2 | MBP for Trajectory = 4 | 2.64 | -0.2 | 1.255 | 0 | No | No |
| Temperature for Trajectory = 2 | 0.12 | -19.3 | Temperature for Trajectory = 2 | 0.42 | 4.5 | 1.158 | 0 | Yes | Yes |
| Temperature for Trajectory = 3 | 0.56 | -8 | Temperature for Trajectory = 3 | 1.03 | 3.9 | 1.158 | 0 | No | No |
| Temperature for Trajectory = 4 | 2.08 | -2.1 | Temperature for Trajectory = 4 | 2.69 | 1.7 | 1.158 | 0 | No | No |
| Urine Output for Trajectory = 2 | 0.06 | -59.7 | Urine Output for Trajectory = 2 | 0.4 | 0.8 | 1.521 | 0 | Yes | Yes |
| Urine Output for Trajectory = 3 | 0.54 | -10.9 | Urine Output for Trajectory = 3 | 0.99 | -0.7 | 1.521 | 0 | Yes | Yes |
| Urine Output for Trajectory = 4 | 2.31 | 9 | Urine Output for Trajectory = 4 | 2.62 | -0.7 | 1.521 | 0 | No | No |
| ALT for Trajectory = 2 | 0.14 | -1.9 | ALT for Trajectory = 2 | 0.4 | -1.4 | 1.092 | 0 | No | No |
| ALT for Trajectory = 3 | 0.61 | -0.3 | ALT for Trajectory = 3 | 0.99 | -0.4 | 1.092 | 0 | No | No |
| ALT for Trajectory = 4 | 2.12 | -0.1 | ALT for Trajectory = 4 | 2.64 | -0.2 | 1.092 | 0 | No | No |
| BUN for Trajectory = 2 | 0.13 | -6.8 | BUN for Trajectory = 2 | 0.41 | 3.1 | 1.445 | 0 | No | No |
| BUN for Trajectory = 3 | 0.64 | 4.3 | BUN for Trajectory = 3 | 1 | 1 | 1.445 | 0 | No | No |
| BUN for Trajectory = 4 | 2.2 | 3.9 | BUN for Trajectory = 4 | 2.65 | 0.4 | 1.445 | 0 | No | No |
| Creatinine for Trajectory = 2 | 0.06 | -56 | Creatinine for Trajectory = 2 | 0.4 | 0.1 | 1.789 | 0 | Yes | Yes |
| Creatinine for Trajectory = 3 | 0.54 | -12.2 | Creatinine for Trajectory = 3 | 0.99 | 0.1 | 1.789 | 0 | Yes | Yes |
| Creatinine for Trajectory = 4 | 2.25 | 6.2 | Creatinine for Trajectory = 4 | 2.62 | -0.8 | 1.789 | 0 | No | No |
| PLT for Trajectory = 2 | 0.15 | 0.8 | PLT for Trajectory = 2 | 0.4 | 0.5 | 1.297 | 0 | No | No |
| PLT for Trajectory = 3 | 0.61 | -0.5 | PLT for Trajectory = 3 | 1 | 0.3 | 1.297 | 0 | No | No |
| PLT for Trajectory = 4 | 2.11 | -0.4 | PLT for Trajectory = 4 | 2.64 | 0.1 | 1.297 | 0 | No | No |
| RBC for Trajectory = 2 | 0.16 | 8.4 | RBC for Trajectory = 2 | 0.4 | 0 | 1.303 | 0 | No | No |
| RBC for Trajectory = 3 | 0.61 | -0.1 | RBC for Trajectory = 3 | 0.99 | 0 | 1.303 | 0 | No | No |
| RBC for Trajectory = 4 | 2.1 | -0.9 | RBC for Trajectory = 4 | 2.64 | 0 | 1.303 | 0 | No | No |
| WBC for Trajectory = 2 | 0.11 | -27 | WBC for Trajectory = 2 | 0.39 | -2.4 | 1.295 | 0 | Yes | Yes |
| WBC for Trajectory = 3 | 0.55 | -10.1 | WBC for Trajectory = 3 | 0.98 | -1.7 | 1.295 | 0 | Yes | Yes |
| WBC for Trajectory = 4 | 2.07 | -2.4 | WBC for Trajectory = 4 | 2.62 | -0.9 | 1.295 | 0 | No | No |
| Admission Type for Trajectory = 2 | 0.12 | -18.8 | Admission Type for Trajectory = 2 | 0.41 | 1.1 | 1.198 | 0 | Yes | Yes |
| Admission Type for Trajectory = 3 | 2.1 | -0.8 | Admission Type for Trajectory = 3 | 1 | 0.5 | 1.198 | 0 | No | No |
| Admission Type for Trajectory = 4 | 0.58 | -4.6 | Admission Type for Trajectory = 4 | 2.64 | 0.1 | 1.198 | 0 | No | No |
| Admission Time for Trajectory = 2 | 0.15 | 4.6 | Admission Time for Trajectory = 2 | 0.4 | -0.4 | 1.092 | 0 | No | No |
| Admission Time for Trajectory = 3 | 2.13 | 0.4 | Admission Time for Trajectory = 3 | 0.99 | -0.4 | 1.092 | 0 | No | No |
| Admission Time for Trajectory = 4 | 0.62 | 1.6 | Admission Time for Trajectory = 4 | 2.64 | 0 | 1.092 | 0 | No | No |
| Traumatic for Trajectory = 2 | 0.22 | 50.1 | Traumatic for Trajectory = 2 | 0.35 | -13.3 | 1.628 | 0 | Yes | Yes |
| Traumatic for Trajectory = 3 | 2.24 | 5.8 | Traumatic for Trajectory = 3 | 0.94 | -5.4 | 1.628 | 0 | Yes | Yes |
| Traumatic for Trajectory = 4 | 0.71 | 17 | Traumatic for Trajectory = 4 | 2.58 | -2.5 | 1.628 | 0 | No | No |
| Hypertension for Trajectory = 2 | 0.24 | 69 | Hypertension for Trajectory = 2 | 0.37 | -7.1 | 1.424 | 0 | Yes | Yes |
| Hypertension for Trajectory = 3 | 2.2 | 3.9 | Hypertension for Trajectory = 3 | 0.94 | -5.4 | 1.424 | 0 | Yes | Yes |
| Hypertension for Trajectory = 4 | 0.75 | 23.2 | Hypertension for Trajectory = 4 | 2.57 | -2.7 | 1.424 | 0 | No | No |
| Diabetes for Trajectory = 2 | 0.15 | 5.2 | Diabetes for Trajectory = 2 | 0.4 | -0.4 | 1.211 | 0 | No | No |
| Diabetes for Trajectory = 3 | 2.11 | -0.4 | Diabetes for Trajectory = 3 | 0.99 | 0.1 | 1.211 | 0 | No | No |
| Diabetes for Trajectory = 4 | 0.63 | 3.8 | Diabetes for Trajectory = 4 | 2.61 | -1.4 | 1.211 | 0 | No | No |
| Liver Disease for Trajectory = 2 | 0.15 | 6.5 | Liver Disease for Trajectory = 2 | 0.4 | -1.2 | 1.155 | 0 | No | No |
| Liver Disease for Trajectory = 3 | 2.14 | 0.9 | Liver Disease for Trajectory = 3 | 0.97 | -2.1 | 1.155 | 0 | No | No |
| Liver Disease for Trajectory = 4 | 0.62 | 2.1 | Liver Disease for Trajectory = 4 | 2.61 | -1.2 | 1.155 | 0 | No | No |
| Stroke History for Trajectory = 2 | 0.14 | -3.7 | Stroke History for Trajectory = 2 | 0.39 | -2.9 | 1.251 | 0 | No | No |
| Stroke History for Trajectory = 3 | 2.14 | 0.9 | Stroke History for Trajectory = 3 | 0.98 | -1.4 | 1.251 | 0 | No | No |
| Stroke History for Trajectory = 4 | 0.61 | -0.9 | Stroke History for Trajectory = 4 | 2.63 | -0.4 | 1.251 | 0 | No | No |
| Initial GCS for Trajectory = 2 | 0.16 | 10.8 | Initial GCS for Trajectory = 2 | 0.38 | -6.2 | 1.33 | 0 | No | No |
| Initial GCS for Trajectory = 3 | 2.1 | -1 | Initial GCS for Trajectory = 3 | 0.94 | -5.3 | 1.33 | 0 | No | No |
| Initial GCS for Trajectory = 4 | 0.62 | 2 | Initial GCS for Trajectory = 4 | 2.59 | -1.8 | 1.33 | 0 | No | No |
| Intubation for Trajectory = 2 | 0.2 | 36.6 | Intubation for Trajectory = 2 | 0.36 | -9.2 | 1.215 | 0 | Yes | Yes |
| Intubation for Trajectory = 3 | 2.23 | 4.9 | Intubation for Trajectory = 3 | 0.95 | -4.1 | 1.215 | 0 | No | No |
| Intubation for Trajectory = 4 | 0.66 | 7.6 | Intubation for Trajectory = 4 | 2.56 | -3.3 | 1.215 | 0 | No | No |
| Vasopressor for Trajectory = 2 | 0.11 | -23.8 | Vasopressor for Trajectory = 2 | 0.4 | 0.3 | 1.22 | 0 | Yes | Yes |
| Vasopressor for Trajectory = 3 | 2.07 | -2.4 | Vasopressor for Trajectory = 3 | 1.01 | 1.5 | 1.22 | 0 | Yes | Yes |
| Vasopressor for Trajectory = 4 | 0.54 | -12.4 | Vasopressor for Trajectory = 4 | 2.69 | 1.7 | 1.22 | 0 | No | No |
| Dialysis for Trajectory = 2 | 0.14 | -4.2 | Dialysis for Trajectory = 2 | 0.4 | 0 | 1.306 | 0 | No | No |
| Dialysis for Trajectory = 3 | 2.08 | -2 | Dialysis for Trajectory = 3 | 1 | 0.5 | 1.306 | 0 | No | No |
| Dialysis for Trajectory = 4 | 0.62 | 0.7 | Dialysis for Trajectory = 4 | 2.61 | -1.2 | 1.306 | 0 | No | No |
| Mannitol for Trajectory = 2 | 0.15 | 2.8 | Mannitol for Trajectory = 2 | 0.4 | 0.3 | 1.211 | 0 | No | No |
| Mannitol for Trajectory = 3 | 2.08 | -2 | Mannitol for Trajectory = 3 | 1.01 | 1.9 | 1.211 | 0 | No | No |
| Mannitol for Trajectory = 4 | 0.61 | -0.6 | Mannitol for Trajectory = 4 | 2.71 | 2.5 | 1.211 | 0 | No | No |
| Craniotomy for Trajectory = 2 | 0.14 | -1.8 | Craniotomy for Trajectory = 2 | 0.4 | -0.2 | 1.1 | 0 | No | No |
| Craniotomy for Trajectory = 3 | 2.11 | -0.5 | Craniotomy for Trajectory = 3 | 0.99 | 0 | 1.1 | 0 | No | No |
| Craniotomy for Trajectory = 4 | 0.6 | -1.1 | Craniotomy for Trajectory = 4 | 2.64 | 0 | 1.1 | 0 | No | No |
| Embolization for Trajectory = 2 | 0.11 | -25.3 | Embolization for Trajectory = 2 | 0.42 | 5.6 | 1.205 | 0 | Yes | Yes |
| Embolization for Trajectory = 3 | 2.06 | -3 | Embolization for Trajectory = 3 | 1.03 | 4.1 | 1.205 | 0 | Yes | Yes |
| Embolization for Trajectory = 4 | 0.55 | -10.2 | Embolization for Trajectory = 4 | 2.67 | 0.9 | 1.205 | 0 | No | No |

**Notes:** Covariates for the final adjusted model were selected using a change-in-estimate approach: a variable was retained if adding it changed the hazard ratio for the GCS-trajectory exposure by ≥10%. Multicollinearity was screened with the generalized variance inflation factor (GVIF); variables with GVIF^(1/(2*Df)) > 2 were flagged for potential collinearity. Selected covariates are shaded: Age, Heart Rate, Temperature, Urine Output, Creatinine, WBC, Admission Type, Traumatic, Hypertension, Intubation, Vasopressor, and Embolization.

# Supplementary Figure 7. Forest Plot of Subgroup HRs for In-Hospital Mortality by ICP Trajectory


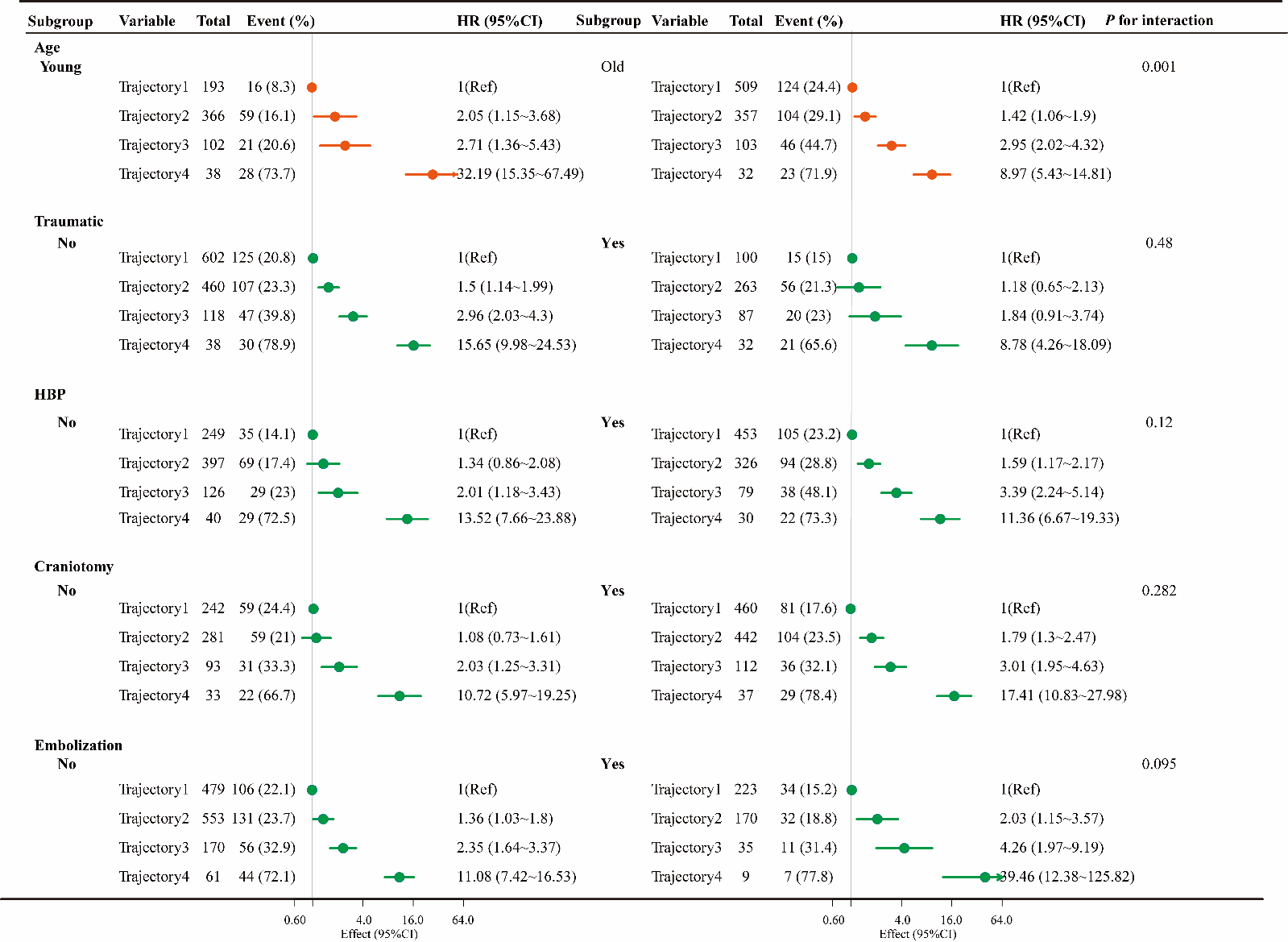


**Legend:** Subgroup analysis of the association between ICP trajectory and in-hospital mortality from the merged dataset, stratified by age, traumatic etiology, HBP, craniotomy, and embolization. Trajectory 1 serves as the reference group. P for interaction tests for effect modification across subgroups

# Supplementary Table **5A**. Subgroup Analysis of the Association Between ICP Trajectory and In-Hospital Mortality in the MIMIC-IV Dataset

| **Subgroup** | **Variable** | **Total (n)** | **Event Rate (%)** | **Follow-up (Time)** | **Crude HR**  **(95% CI)** | **Crude**  ***P*** | **Adjusted HR**  **(95% CI)** | **Adjusted**  ***P*** | **Interaction *P*** |
| --- | --- | --- | --- | --- | --- | --- | --- | --- | --- |
| **Age** | |  |  |  |  |  |  |  | 0.011 |
| **Young** | Trajectory 1 | 125 | 8 (6.4) | 2997.001 | 1(Ref) |  | 1(Ref) |  |  |
|  | Trajectory 2 | 128 | 24 (18.8) | 3153.266 | 2.92 (1.31~6.52) | 0.009 | 7.2 (2.88~18.02) | <0.001 |  |
|  | Trajectory 3 | 13 | 9 (69.2) | 151.1917 | 23.38 (8.97~60.96) | <0.001 | 250.73 (54.24~1159.05) | <0.001 |  |
|  | Trajectory 4 | 5 | 5 (100) | 9.966667 | 156.95 (42.52~579.29) | <0.001 | 236.8 (45.1~1243.32) | <0.001 |  |
|  |  |  |  |  |  |  |  |  |  |
| **Old** | Trajectory 1 | 359 | 108 (30.1) | 7728.304 | 1(Ref) |  | 1(Ref) |  |  |
|  | Trajectory 2 | 102 | 36 (35.3) | 1733.072 | 1.41 (0.97~2.06) | 0.075 | 1.71 (1.12~2.61) | 0.012 |  |
|  | Trajectory 3 | 9 | 8 (88.9) | 44.95694 | 9.95 (4.81~20.59) | <0.001 | 7.74 (3.39~17.66) | <0.001 |  |
|  | Trajectory 4 | 5 | 5 (100) | 12.36944 | 23.13 (9.16~58.4) | <0.001 | 23.15 (8.44~63.51) | <0.001 |  |
| **Traumatic** | |  |  |  |  |  |  |  | 0.303 |
| **No** | Trajectory 1 | 449 | 108 (24.1) | 9773.076 | 1(Ref) |  | 1(Ref) |  |  |
|  | Trajectory 2 | 152 | 47 (30.9) | 2864.108 | 1.46 (1.03~2.06) | 0.032 | 1.97 (1.36~2.85) | <0.001 |  |
|  | Trajectory 3 | 14 | 12 (85.7) | 116.7438 | 9.54 (5.23~17.38) | <0.001 | 9.3 (4.83~17.93) | <0.001 |  |
|  | Trajectory 4 | 8 | 8 (100) | 18.93125 | 32.24 (15.11~68.78) | <0.001 | 30.86 (13.38~71.17) | <0.001 |  |
|  |  |  |  |  |  |  |  |  |  |
| **Yes** | Trajectory 1 | 35 | 8 (22.9) | 952.2299 | 1(Ref) |  | 1(Ref) |  |  |
|  | Trajectory 2 | 78 | 13 (16.7) | 2022.23 | 0.69 (0.29~1.67) | 0.41 | 1.51 (0.55~4.11) | 0.42 |  |
|  | Trajectory 3 | 8 | 5 (62.5) | 79.40486 | 5.95 (1.93~18.37) | 0.002 | 50.33 (8.7~290.96) | <0.001 |  |
|  | Trajectory 4 | 2 | 2 (100) | 3.404861 | 44.18 (7.13~273.66) | <0.001 | 17.08 (1.09~268.12) | 0.043 |  |
| **HBP** | |  |  |  |  |  |  |  | 0.169 |
| **No** | Trajectory 1 | 153 | 21 (13.7) | 3582.923 | 1(Ref) |  | 1(Ref) |  |  |
|  | Trajectory 2 | 124 | 21 (16.9) | 2837.229 | 1.33 (0.72~2.45) | 0.364 | 2.66 (1.34~5.29) | 0.005 |  |
|  | Trajectory 3 | 12 | 8 (66.7) | 113.9833 | 10.37 (4.54~23.68) | <0.001 | 25.25 (7.91~80.61) | <0.001 |  |
|  | Trajectory 4 | 6 | 6 (100) | 10.46111 | 64.37 (22.29~185.9) | <0.001 | 55.33 (16.04~190.8) | <0.001 |  |
|  |  |  |  |  |  |  |  |  |  |
| **Yes** | Trajectory 1 | 331 | 95 (28.7) | 7142.383 | 1(Ref) |  | 1(Ref) |  |  |
|  | Trajectory 2 | 106 | 39 (36.8) | 2049.108 | 1.42 (0.98~2.07) | 0.064 | 1.81 (1.18~2.76) | 0.006 |  |
|  | Trajectory 3 | 10 | 9 (90) | 82.16528 | 9.41 (4.73~18.7) | <0.001 | 14.13 (6.33~31.54) | <0.001 |  |
|  | Trajectory 4 | 4 | 4 (100) | 11.875 | 21.54 (7.72~60.12) | <0.001 | 22.86 (6.88~75.99) | <0.001 |  |
| **Craniotomy** | |  |  |  |  |  |  |  | 0.158 |
| **No** | Trajectory 1 | 171 | 47 (27.5) | 3631.628 | 1(Ref) |  | 1(Ref) |  |  |
|  | Trajectory 2 | 65 | 18 (27.7) | 1282.309 | 1.08 (0.63~1.87) | 0.77 | 1.21 (0.64~2.29) | 0.564 |  |
|  | Trajectory 3 | 7 | 4 (57.1) | 88.31111 | 4.66 (1.67~13.03) | 0.003 | 14.18 (4.16~48.28) | <0.001 |  |
|  | Trajectory 4 | 3 | 3 (100) | 9.344444 | 14.97 (4.52~49.55) | <0.001 | 12.47 (3.44~45.21) | <0.001 |  |
|  |  |  |  |  |  |  |  |  |  |
| **Yes** | Trajectory 1 | 313 | 69 (22) | 7093.677 | 1(Ref) |  | 1(Ref) |  |  |
|  | Trajectory 2 | 165 | 42 (25.5) | 3604.028 | 1.23 (0.83~1.8) | 0.298 | 2.53 (1.62~3.93) | <0.001 |  |
|  | Trajectory 3 | 15 | 13 (86.7) | 107.8375 | 10.62 (5.84~19.32) | <0.001 | 12.74 (6.5~24.95) | <0.001 |  |
|  | Trajectory 4 | 7 | 7 (100) | 12.99167 | 72.27 (28.65~182.3) | <0.001 | 71.56 (23.46~218.32) | <0.001 |  |
| **Embolization** | |  |  |  |  |  |  |  | 0.756 |
| **No** | Trajectory 1 | 284 | 83 (29.2) | 5837.352 | 1(Ref) |  | 1(Ref) |  |  |
|  | Trajectory 2 | 165 | 45 (27.3) | 3649.771 | 0.94 (0.65~1.35) | 0.737 | 1.69 (1.12~2.54) | 0.013 |  |
|  | Trajectory 3 | 17 | 13 (76.5) | 126.334 | 6.7 (3.72~12.06) | <0.001 | 9.96 (5.19~19.1) | <0.001 |  |
|  | Trajectory 4 | 8 | 8 (100) | 17.39444 | 23.34 (10.9~49.97) | <0.001 | 21.85 (9.09~52.55) | <0.001 |  |
|  |  |  |  |  |  |  |  |  |  |
| **Yes** | Trajectory 1 | 200 | 33 (16.5) | 4887.953 | 1(Ref) |  | 1(Ref) |  |  |
|  | Trajectory 2 | 65 | 15 (23.1) | 1236.567 | 1.77 (0.96~3.28) | 0.068 | 3.08 (1.67~5.65) | <0.001 |  |
|  | Trajectory 3 | 5 | 4 (80) | 69.81458 | 10.87 (3.81~30.99) | <0.001 | 35.68 (13.43~94.79) | <0.001 |  |
|  | Trajectory 4 | 2 | 2 (100) | 4.941667 | 98.99 (17.63~555.9) | <0.001 | 213.11 (34.84~1303.73) | <0.001 |  |

**Notes:** Subgroup analysis of the association between ICP trajectory and in-hospital mortality in the MIMIC-IV dataset.

# Supplementary Table **5B**. Subgroup Analysis of the Association Between ICP Trajectory and In-Hospital Mortality in the eICU Dataset

| **Subgroup** | **Variable** | | **Total (n)** | **Event Rate (%)** | **Follow-up (Time)** | **Crude HR**  **(95% CI)** | **Crude**  ***P*** | **Adjusted HR**  **(95% CI)** | **Adjusted**  ***P*** | **Interaction *P*** |
| --- | --- | --- | --- | --- | --- | --- | --- | --- | --- | --- |
| **Age** | |  |  |  |  |  |  |  |  | 0.422 |
| **Young** | Trajectory 1 | | 65 | 9 (13.8) | 1490.042 | 1(Ref) |  | 1(Ref) |  |  |
|  | Trajectory 2 | | 230 | 41 (17.8) | 5327.875 | 1.3 (0.63~2.67) | 0.477 | 1.48 (0.71~3.1) | 0.297 |  |
|  | Trajectory 3 | | 44 | 14 (31.8) | 862.125 | 2.57 (1.11~5.94) | 0.027 | 3.14 (1.31~7.54) | 0.01 |  |
|  | Trajectory 4 | | 9 | 6 (66.7) | 69.375 | 9.22 (3.26~26.08) | <0.001 | 9.23 (3.09~27.6) | <0.001 |  |
|  |  | |  |  |  |  |  |  |  |  |
| **Old** | Trajectory 1 | | 115 | 36 (31.3) | 1975.375 | 1(Ref) |  | 1(Ref) |  |  |
|  | Trajectory 2 | | 212 | 59 (27.8) | 4029.542 | 0.86 (0.57~1.3) | 0.48 | 0.76 (0.5~1.17) | 0.213 |  |
|  | Trajectory 3 | | 46 | 25 (54.3) | 576.625 | 2.24 (1.34~3.75) | 0.002 | 2.13 (1.23~3.69) | 0.007 |  |
|  | Trajectory 4 | | 9 | 8 (88.9) | 72.45833 | 6.08 (2.81~13.14) | <0.001 | 7.6 (3.3~17.46) | <0.001 |  |
| **Traumatic** | |  |  |  |  |  |  |  |  | 0.799 |
| **No** | Trajectory 1 | | 114 | 30 (26.3) | 2174.042 | 1(Ref) |  | 1(Ref) |  |  |
|  | Trajectory 2 | | 261 | 58 (22.2) | 5638.417 | 0.83 (0.53~1.29) | 0.406 | 0.92 (0.59~1.45) | 0.718 |  |
|  | Trajectory 3 | | 49 | 23 (46.9) | 714.875 | 2.16 (1.25~3.71) | 0.006 | 2.5 (1.4~4.45) | 0.002 |  |
|  | Trajectory 4 | | 10 | 9 (90) | 62.79167 | 7.43 (3.5~15.76) | <0.001 | 9.89 (4.51~21.73) | <0.001 |  |
|  |  | |  |  |  |  |  |  |  |  |
| **Yes** | Trajectory 1 | | 66 | 15 (22.7) | 1291.375 | 1(Ref) |  | 1(Ref) |  |  |
|  | Trajectory 2 | | 181 | 42 (23.2) | 3719 | 1.01 (0.56~1.82) | 0.98 | 1.51 (0.8~2.85) | 0.203 |  |
|  | Trajectory 3 | | 41 | 16 (39) | 723.875 | 1.93 (0.95~3.9) | 0.068 | 3.71 (1.73~7.93) | 0.001 |  |
|  | Trajectory 4 | | 8 | 5 (62.5) | 79.04167 | 5.28 (1.91~14.58) | 0.001 | 14.08 (4.45~44.53) | <0.001 |  |
| **HBP** | |  |  |  |  |  |  |  |  | 0.763 |
| **No** | Trajectory 1 | | 96 | 20 (20.8) | 1983.167 | 1(Ref) |  | 1(Ref) |  |  |
|  | Trajectory 2 | | 268 | 52 (19.4) | 5815.208 | 0.94 (0.56~1.58) | 0.827 | 1.02 (0.6~1.74) | 0.931 |  |
|  | Trajectory 3 | | 56 | 18 (32.1) | 1044.083 | 1.77 (0.94~3.35) | 0.079 | 2.38 (1.22~4.65) | 0.011 |  |
|  | Trajectory 4 | | 10 | 7 (70) | 79.5 | 6.26 (2.63~14.89) | <0.001 | 10.2 (4.09~25.47) | <0.001 |  |
|  |  | |  |  |  |  |  |  |  |  |
| **Yes** | Trajectory 1 | | 84 | 25 (29.8) | 1482.25 | 1(Ref) |  | 1(Ref) |  |  |
|  | Trajectory 2 | | 174 | 48 (27.6) | 3542.208 | 0.88 (0.54~1.42) | 0.591 | 0.84 (0.51~1.38) | 0.499 |  |
|  | Trajectory 3 | | 34 | 21 (61.8) | 394.6667 | 2.71 (1.51~4.86) | 0.001 | 2.64 (1.42~4.88) | 0.002 |  |
|  | Trajectory 4 | | 8 | 7 (87.5) | 62.33333 | 6.35 (2.73~14.73) | <0.001 | 8.33 (3.37~20.64) | <0.001 |  |
| **Craniotomy** | |  |  |  |  |  |  |  |  | 0.968 |
| **No** | Trajectory 1 | | 78 | 17 (21.8) | 1511.375 | 1(Ref) |  | 1(Ref) |  |  |
|  | Trajectory 2 | | 209 | 44 (21.1) | 4164.958 | 0.93 (0.53~1.63) | 0.81 | 0.96 (0.54~1.72) | 0.902 |  |
|  | Trajectory 3 | | 53 | 23 (43.4) | 824.5 | 2.24 (1.2~4.19) | 0.012 | 2.31 (1.21~4.41) | 0.011 |  |
|  | Trajectory 4 | | 11 | 8 (72.7) | 105.625 | 5.72 (2.46~13.28) | <0.001 | 7.55 (2.96~19.27) | <0.001 |  |
|  |  | |  |  |  |  |  |  |  |  |
| **Yes** | Trajectory 1 | | 102 | 28 (27.5) | 1954.042 | 1(Ref) |  | 1(Ref) |  |  |
|  | Trajectory 2 | | 233 | 56 (24) | 5192.458 | 0.87 (0.55~1.36) | 0.534 | 0.96 (0.61~1.52) | 0.865 |  |
|  | Trajectory 3 | | 37 | 16 (43.2) | 614.25 | 1.89 (1.02~3.5) | 0.042 | 2.33 (1.22~4.45) | 0.01 |  |
|  | Trajectory 4 | | 7 | 6 (85.7) | 36.20833 | 8.84 (3.56~21.93) | <0.001 | 11.37 (4.35~29.69) | <0.001 |  |
| **Embolization** | |  |  |  |  |  |  |  |  | 0.409 |
| **No** | Trajectory 1 | | 145 | 40 (27.6) | 2644.542 | 1(Ref) |  | 1(Ref) |  |  |
|  | Trajectory 2 | | 353 | 84 (23.8) | 7106.833 | 0.83 (0.57~1.22) | 0.346 | 0.9 (0.62~1.32) | 0.599 |  |
|  | Trajectory 3 | | 75 | 33 (44) | 1209.833 | 1.8 (1.14~2.86) | 0.012 | 2.3 (1.43~3.7) | 0.001 |  |
|  | Trajectory 4 | | 16 | 12 (75) | 135.5833 | 4.86 (2.54~9.29) | <0.001 | 7.2 (3.65~14.18) | <0.001 |  |
|  |  | |  |  |  |  |  |  |  |  |
| **Yes** | Trajectory 1 | | 35 | 5 (14.3) | 820.875 | 1(Ref) |  | 1(Ref) |  |  |
|  | Trajectory 2 | | 89 | 16 (18) | 2250.583 | 1.27 (0.47~3.48) | 0.638 | 1.56 (0.5~4.94) | 0.446 |  |
|  | Trajectory 3 | | 15 | 6 (40) | 228.9167 | 4 (1.21~13.21) | 0.023 | 6.03 (1.23~29.53) | 0.027 |  |
|  | Trajectory 4 | | 2 | 2 (100) | 6.25 | 52.96 (8.99~311.95) | <0.001 | 49.03 (4.5~534.1) | 0.001 |  |

**Note:** Subgroup analysis of the association between ICP trajectory and in-hospital mortality in the eICU dataset.

.

# Supplementary Table **5C**. Subgroup Analysis of the Association Between ICP Trajectory and In-Hospital Mortality in the NSICU Dataset

| **Subgroup** | **Variable** | | **Total (n)** | **Event Rate (%)** | **Follow-up (Time)** | **Crude HR**  **(95% CI)** | **Crude**  ***P*** | **Adjusted HR**  **(95% CI)** | **Adjusted**  ***P*** | **Interaction *P*** |
| --- | --- | --- | --- | --- | --- | --- | --- | --- | --- | --- |
| **Age** | |  |  |  |  |  |  |  |  | 0.566 |
| **Young** | Trajectory 1 | | 51 | 2 (3.9) | 718 | 1(Ref) |  | 1(Ref) |  |  |
|  | Trajectory 2 | | 17 | 1 (5.9) | 255 | 1.52 (0.14~16.77) | 0.733 | 0 (0~Inf) | 0.987 |  |
|  | Trajectory 3 | | 8 | 2 (25) | 123 | 5.57 (0.77~40.24) | 0.088 | NA | <0.001 |  |
|  | Trajectory 4 | | 4 | 3 (75) | 34 | 15.47 (2.35~101.91) | 0.004 | 1318.2 (82.45~21075.8) | <0.001 |  |
|  |  | |  |  |  |  |  |  |  |  |
| **Old** | Trajectory 1 | | 111 | 5 (4.5) | 1697 | 1(Ref) |  | 1(Ref) |  |  |
|  | Trajectory 2 | | 20 | 2 (10) | 308 | 2.84 (0.52~15.51) | 0.228 | 10.35 (2.11~50.83) | 0.004 |  |
|  | Trajectory 3 | | 12 | 4 (33.3) | 149 | 13.24 (3.3~53.09) | <0.001 | 9.12 (2.64~31.51) | <0.001 |  |
|  | Trajectory 4 | | 1 | 1 (100) | 4 | 99.38 (9~1097.43) | <0.001 | 3.9 (0.4~37.78) | 0.241 |  |
| **Traumatic** | |  |  |  |  |  |  |  |  | 0.408 |
| **No** | Trajectory 1 | | 122 | 6 (4.9) | 1865 | 1(Ref) |  | 1(Ref) |  |  |
|  | Trajectory 2 | | 25 | 2 (8) | 379 | 1.95 (0.38~10.06) | 0.424 | 11.4 (0.81~161.11) | 0.072 |  |
|  | Trajectory 3 | | 10 | 2 (20) | 144 | 5.28 (1.02~27.49) | 0.048 | 3.95 (0.14~109.47) | 0.417 |  |
|  | Trajectory 4 | | 4 | 4 (100) | 34 | 40.94 (10.3~162.66) | <0.001 | 2356.56 (44.76~124068.23) | <0.001 |  |
|  |  | |  |  |  |  |  |  |  |  |
| **Yes** | Trajectory 1 | | 40 | 1 (2.5) | 550 | 1(Ref) |  | 1(Ref) |  |  |
|  | Trajectory 2 | | 12 | 1 (8.3) | 184 | 2.82 (0.17~45.89) | 0.466 | 0 (0~Inf) | 0.973 |  |
|  | Trajectory 3 | | 10 | 4 (40) | 128 | 17.65 (1.95~160.04) | 0.011 | NA | 0.996 |  |
|  | Trajectory 4 | | 1 | 0 (0) | 4 | 0 (0~Inf) | 0.999 | NA | 0.999 |  |
| **HBP** | |  |  |  |  |  |  |  |  | 0.014 |
| **No** | Trajectory 1 | | 64 | 4 (6.2) | 932 | 1(Ref) |  | 1(Ref) |  |  |
|  | Trajectory 2 | | 11 | 1 (9.1) | 139 | 1.64 (0.18~14.85) | 0.658 | 0 (0~0) | <0.001 |  |
|  | Trajectory 3 | | 10 | 3 (30) | 112 | 5.9 (1.27~27.33) | 0.023 | 0 (0~0) | <0.001 |  |
|  | Trajectory 4 | | 2 | 1 (50) | 7 | 19.37 (1.91~196.38) | 0.012 | 0 (0~0) | <0.001 |  |
|  |  | |  |  |  |  |  |  |  |  |
| **Yes** | Trajectory 1 | | 98 | 3 (3.1) | 1483 | 1(Ref) |  | 1(Ref) |  |  |
|  | Trajectory 2 | | 26 | 2 (7.7) | 424 | 2.42 (0.4~14.47) | 0.334 | 182.67 (1.48~ Inf) | 0.034 |  |
|  | Trajectory 3 | | 10 | 3 (30) | 160 | 8.93 (1.77~45.01) | 0.008 | 0.49 (0.01~17.88) | 0.7 |  |
|  | Trajectory 4 | | 3 | 3 (100) | 31 | 36.22 (6.62~198.04) | <0.001 | 2841.31 (9.43~ Inf) | 0.006 |  |
| **Craniotomy** | |  |  |  |  |  |  |  |  | 0.655 |
| **No** | Trajectory 1 | | 34 | 3 (8.8) | 502 | 1(Ref) |  | 1(Ref) |  |  |
|  | Trajectory 2 | | 11 | 1 (9.1) | 188 | 0.93 (0.1~8.93) | 0.948 | NA | <0.001 |  |
|  | Trajectory 3 | | 6 | 2 (33.3) | 98 | 4.17 (0.69~24.98) | 0.118 | NA | <0.001 |  |
|  | Trajectory 4 | | 1 | 1 (100) | 1 | NA (NA~NA) | NA | NA | 0.999 |  |
|  |  | |  |  |  |  |  |  |  |  |
| **Yes** | Trajectory 1 | | 128 | 4 (3.1) | 1913 | 1(Ref) |  | 1(Ref) |  |  |
|  | Trajectory 2 | | 26 | 2 (7.7) | 375 | 2.77 (0.5~15.21) | 0.242 | 7.28 (0.34~155.58) | 0.204 |  |
|  | Trajectory 3 | | 14 | 4 (28.6) | 174 | 9.94 (2.43~40.69) | 0.001 | 2.36 (0.07~84.92) | 0.639 |  |
|  | Trajectory 4 | | 4 | 3 (75) | 37 | 29.91 (5.95~150.29) | <0.001 | 4.56 (0.14~149.06) | 0.394 |  |
| **Embolization** | |  |  |  |  |  |  |  |  | 0.253 |
| **No** | Trajectory 1 | | 143 | 7 (4.9) | 2102 | 1(Ref) |  | 1(Ref) |  |  |
|  | Trajectory 2 | | 33 | 3 (9.1) | 496 | 1.95 (0.5~7.56) | 0.337 | 5.3 (1.47~19.16) | 0.011 |  |
|  | Trajectory 3 | | 20 | 6 (30) | 272 | 6.71 (2.23~20.21) | 0.001 | 4.74 (1.75~12.82) | 0.002 |  |
|  | Trajectory 4 | | 4 | 3 (75) | 12 | 51.63 (11.85~224.96) | <0.001 | 24.2 (6.05~96.74) | <0.001 |  |
|  |  | |  |  |  |  |  |  |  |  |
| **Yes** | Trajectory 1 | | 19 | 0 (0) | 313 | 1(Ref) |  | 1(Ref) |  |  |
|  | Trajectory 2 | | 4 | 0 (0) | 67 | 1 (1~1) | NaN | 1 (1~1) | NaN |  |
|  | Trajectory 3 | | 0 | 0 (NaN) | 0 | 1 (1~1) | NaN | 1 (1~1) | NaN |  |
|  | Trajectory 4 | | 1 | 1 (100) | 26 | NA | 0.999 | NA | 0.999 |  |

**Note:** Subgroup analysis of the association between ICP trajectory and in-hospital mortality in the NSICU database. **NA** indicates insufficient data for calculation, and **NaN** indicates invalid values due to undefined operations (e.g., zero events).

# Supplementary Figure 8. ICP Trajectories in Elderly Patients Across Cohorts


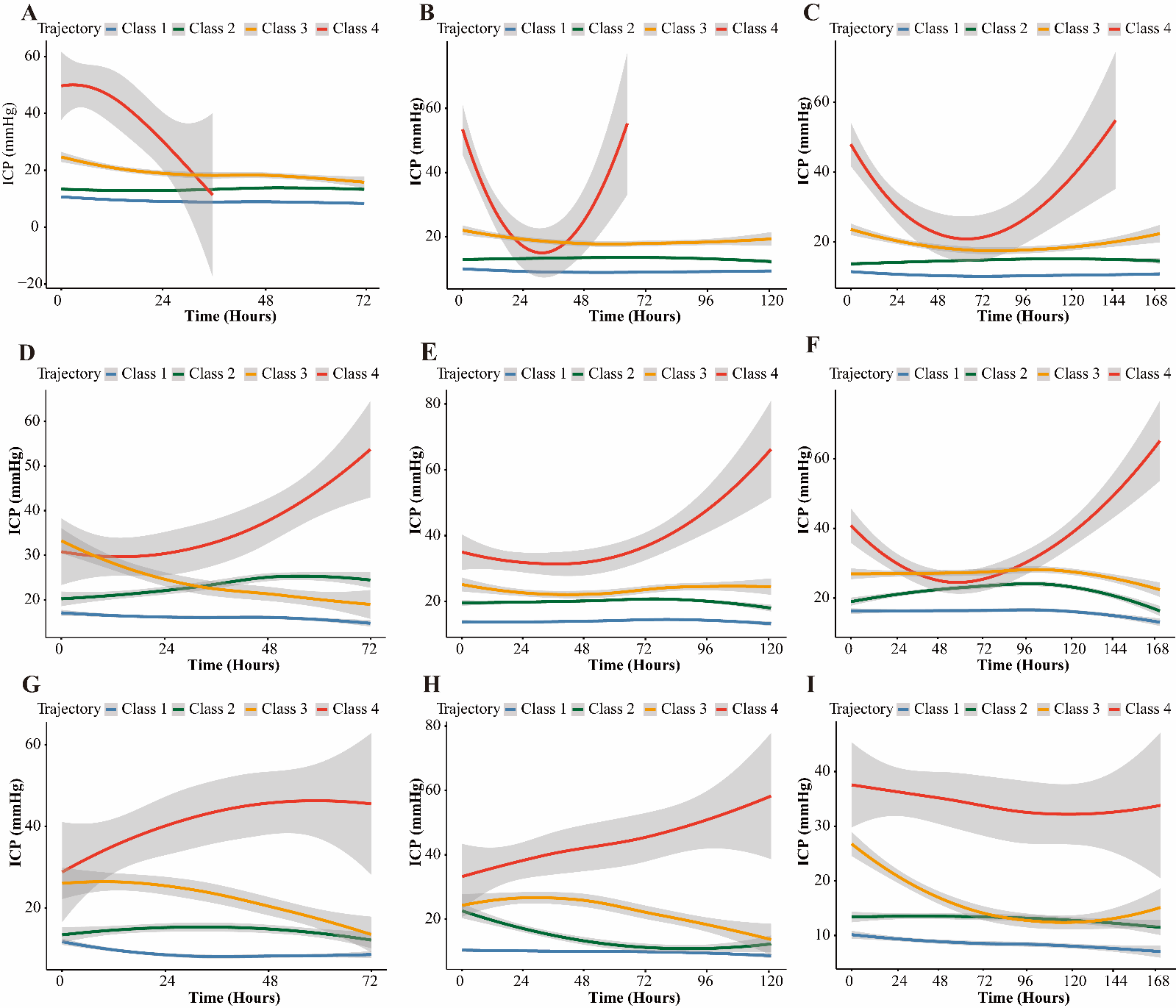


**Legend:** LCGM-derived ICP trajectories for patients aged ≥55 years, stratified by dataset: MIMIC-IV (Panels A–C), eICU (Panels D–F), and NSICU (Panels G–I). Within each dataset, trajectories are shown over 72, 120, and 168 hours, respectively. The curves represent the class-specific mean ICP, and the shaded areas indicate the 95% CIs

# Supplementary Table 6A. Model Fit Statistics for 72-Hour ICP Trajectories in Elderly Patients in the Integrated Dataset

| **G** | **Log-Likelihood** | **AIC** | **BIC** | **SABIC** | **Entropy** | **Class Proportion (%)** | | | | | | **Average Posterior Probability** | | | | | |
| --- | --- | --- | --- | --- | --- | --- | --- | --- | --- | --- | --- | --- | --- | --- | --- | --- | --- |
|  |  |  |  |  |  | **1** | **2** | **3** | **4** | **5** | **6** | **1** | **2** | **3** | **4** | **5** | **6** |
| 2 | -47088.05 | 94206.10 | 94279.73 | 94232.09 | 0.80 | 88.51 | 11.49 |  |  |  |  | 0.96 | 0.90 |  |  |  |  |
| 3 | -46951.89 | 93943.78 | 94041.96 | 93978.44 | 0.71 | 48.85 | 7.89 | 43.26 |  |  |  | 0.87 | 0.90 | 0.88 |  |  |  |
| 4 | -46903.49 | 93856.98 | 93979.70 | 93900.30 | 0.73 | 43.66 | 23.98 | 27.07 | 5.29 |  |  | 0.88 | 0.75 | 0.86 | 0.93 |  |  |
| 5 | -46893.85 | 93847.69 | 93994.96 | 93899.67 | 0.68 | 42.66 | 21.28 | 21.88 | 3.80 | 10.39 |  | 0.86 | 0.71 | 0.73 | 0.89 | 0.75 |  |
| 6 | -46904.77 | 93879.54 | 94051.35 | 93940.19 | 0.68 | 42.66 | 20.58 | 11.09 | 3.60 | 3.50 | 18.58 | 0.86 | 0.68 | 0.60 | 0.60 | 0.91 | 0.77 |

**Note:** Model fit statistics for the 72-hour ICP LCGM for patients aged ≥55 years.

# Supplementary Table 6B. Model Fit Statistics for 120-Hour ICP Trajectories in Elderly Patients in the Integrated Dataset

| **G** | **Log-Likelihood** | **AIC** | **BIC** | **SABIC** | **Entropy** | **Class Proportion (%)** | | | | | | **Average Posterior Probability** | | | | | |
| --- | --- | --- | --- | --- | --- | --- | --- | --- | --- | --- | --- | --- | --- | --- | --- | --- | --- |
|  |  |  |  |  |  | **1** | **2** | **3** | **4** | **5** | **6** | **1** | **2** | **3** | **4** | **5** | **6** |
| 2 | -86601.57 | 173233.15 | 173306.78 | 173259.14 | 0.83 | 90.01 | 9.99 |  |  |  |  | 0.97 | 0.91 |  |  |  |  |
| 3 | -86440.43 | 172920.86 | 173019.03 | 172955.51 | 0.74 | 53.95 | 41.66 | 4.40 |  |  |  | 0.88 | 0.89 | 0.95 |  |  |  |
| 4 | -86395.78 | 172841.57 | 172964.29 | 172884.89 | 0.70 | 41.46 | 25.67 | 28.67 | 4.20 |  |  | 0.85 | 0.73 | 0.88 | 0.94 |  |  |
| 5 | -86374.77 | 172809.54 | 172956.80 | 172861.52 | 0.73 | 41.36 | 29.87 | 24.18 | 0.70 | 3.90 |  | 0.84 | 0.85 | 0.73 | 0.90 | 0.84 |  |
| 6 | -86363.75 | 172797.50 | 172969.30 | 172858.14 | 0.68 | 10.89 | 33.67 | 21.88 | 28.87 | 0.70 | 4.00 | 0.76 | 0.69 | 0.73 | 0.86 | 0.91 | 0.83 |

# Supplementary Table 6C. Model Fit Statistics for 168-Hour ICP Trajectories in Elderly Patients in the Integrated Dataset

| **G** | **Log-Likelihood** | **AIC** | **BIC** | **SABIC** | **Entropy** | **Class Proportion (%)** | | | | | | **Average Posterior Probability** | | | | | |
| --- | --- | --- | --- | --- | --- | --- | --- | --- | --- | --- | --- | --- | --- | --- | --- | --- | --- |
|  |  |  |  |  |  | **1** | **2** | **3** | **4** | **5** | **6** | **1** | **2** | **3** | **4** | **5** | **6** |
| 2 | -58771.53 | 117573.06 | 117646.69 | 117599.05 | 0.64 | 62.54 | 37.46 |  |  |  |  | 0.89 | 0.92 |  |  |  |  |
| 3 | -58603.03 | 117246.06 | 117344.23 | 117280.71 | 0.74 | 52.95 | 5.79 | 41.26 |  |  |  | 0.88 | 0.90 | 0.90 |  |  |  |
| 4 | -58568.69 | 117187.37 | 117310.09 | 117230.69 | 0.68 | 42.06 | 20.68 | 32.37 | 4.90 |  |  | 0.83 | 0.71 | 0.85 | 0.93 |  |  |
| 5 | -58571.78 | 117203.56 | 117350.82 | 117255.54 | 0.63 | 42.46 | 13.89 | 21.58 | 3.40 | 18.68 |  | 0.82 | 0.62 | 0.66 | 0.92 | 0.78 |  |
| 6 | -58561.36 | 117192.71 | 117364.52 | 117253.36 | 0.67 | 41.76 | 13.59 | 25.47 | 0.80 | 3.70 | 14.69 | 0.82 | 0.63 | 0.71 | 0.72 | 0.91 | 0.77 |

# Supplementary Table 6D. Model Fit Statistics for 72-Hour ICP Trajectories in Elderly Patients in the MIMIC-IV Dataset

| **G** | **Log-Likelihood** | **AIC** | **BIC** | **SABIC** | **Entropy** | **Class Proportion (%)** | | | | | | **Average Posterior Probability** | | | | | |
| --- | --- | --- | --- | --- | --- | --- | --- | --- | --- | --- | --- | --- | --- | --- | --- | --- | --- |
|  |  |  |  |  |  | **1** | **2** | **3** | **4** | **5** | **6** | **1** | **2** | **3** | **4** | **5** | **6** |
| 2 | -20549.06 | 41128.11 | 41190.56 | 41142.95 | 0.83 | 84.42 | 15.58 |  |  |  |  | 0.97 | 0.92 |  |  |  |  |
| 3 | -20486.24 | 41012.48 | 41095.75 | 41032.27 | 0.74 | 66.32 | 6.11 | 27.58 |  |  |  | 0.89 | 0.90 | 0.88 |  |  |  |
| 4 | -20447.08 | 40944.17 | 41048.25 | 40968.91 | 0.72 | 50.11 | 32.63 | 15.16 | 2.11 |  |  | 0.84 | 0.80 | 0.91 | 0.99 |  |  |
| 5 | -20415.12 | 40890.23 | 41015.13 | 40919.91 | 0.80 | 48.84 | 33.47 | 13.26 | 0.84 | 3.58 |  | 0.88 | 0.80 | 0.93 | 1.00 | 0.99 |  |
| 6 | -20428.10 | 40926.20 | 41071.92 | 40960.83 | 0.64 | 34.11 | 30.74 | 16.00 | 8.21 | 2.11 | 8.84 | 0.76 | 0.63 | 0.72 | 0.79 | 0.99 | 0.91 |

# Supplementary Table 6E. Model Fit Statistics for 120-Hour ICP Trajectories in Elderly Patients in the MIMIC-IV Dataset

| **G** | **Log-Likelihood** | **AIC** | **BIC** | **SABIC** | **Entropy** | **Class Proportion (%)** | | | | | | **Average Posterior Probability** | | | | | |
| --- | --- | --- | --- | --- | --- | --- | --- | --- | --- | --- | --- | --- | --- | --- | --- | --- | --- |
|  |  |  |  |  |  | **1** | **2** | **3** | **4** | **5** | **6** | **1** | **2** | **3** | **4** | **5** | **6** |
| 2 | -37238.58 | 74507.15 | 74569.60 | 74522.00 | 0.86 | 86.32 | 13.68 |  |  |  |  | 0.98 | 0.91 |  |  |  |  |
| 3 | -37164.16 | 74368.32 | 74451.59 | 74388.11 | 0.79 | 76.00 | 2.95 | 21.05 |  |  |  | 0.92 | 0.96 | 0.87 |  |  |  |
| 4 | -37144.00 | 74338.00 | 74442.08 | 74362.74 | 0.65 | 54.74 | 31.37 | 11.37 | 2.53 |  |  | 0.79 | 0.76 | 0.85 | 0.98 |  |  |
| 5 | -37130.45 | 74320.90 | 74445.80 | 74350.59 | 0.71 | 60.21 | 25.05 | 3.16 | 2.74 | 8.84 |  | 0.81 | 0.78 | 0.81 | 0.95 | 0.89 |  |
| 6 | -37130.75 | 74331.51 | 74477.22 | 74366.14 | 0.72 | 8.84 | 56.84 | 18.74 | 2.95 | 2.74 | 9.89 | 0.74 | 0.79 | 0.79 | 0.85 | 0.96 | 0.88 |

# Supplementary Table 6F. Model Fit Statistics for 168-Hour ICP Trajectories in Elderly Patients in the MIMIC-IV Dataset

| **G** | **Log-Likelihood** | **AIC** | **BIC** | **SABIC** | **Entropy** | **Class Proportion (%)** | | | | | | **Average Posterior Probability** | | | | | |
| --- | --- | --- | --- | --- | --- | --- | --- | --- | --- | --- | --- | --- | --- | --- | --- | --- | --- |
|  |  |  |  |  |  | **1** | **2** | **3** | **4** | **5** | **6** | **1** | **2** | **3** | **4** | **5** | **6** |
| 2 | -25576.33 | 51182.66 | 51245.11 | 51197.50 | 0.84 | 83.79 | 16.21 |  |  |  |  | 0.97 | 0.93 |  |  |  |  |
| 3 | -25505.69 | 51051.38 | 51134.65 | 51071.17 | 0.80 | 76.84 | 3.58 | 19.58 |  |  |  | 0.93 | 0.94 | 0.88 |  |  |  |
| 4 | -25485.60 | 51021.19 | 51125.27 | 51045.93 | 0.66 | 49.89 | 30.95 | 15.58 | 3.58 |  |  | 0.80 | 0.75 | 0.87 | 0.93 |  |  |
| 5 | -25481.32 | 51022.64 | 51147.54 | 51052.33 | 0.67 | 56.42 | 18.95 | 4.42 | 3.37 | 16.84 |  | 0.81 | 0.68 | 0.64 | 0.96 | 0.84 |  |
| 6 | -25476.83 | 51023.66 | 51169.37 | 51058.29 | 0.71 | 59.79 | 13.68 | 1.47 | 6.74 | 3.79 | 14.53 | 0.83 | 0.70 | 0.87 | 0.58 | 0.93 | 0.86 |

# Supplementary Table 6G. Model Fit Statistics for 72-Hour ICP Trajectories in Elderly Patients in the eICU Dataset

| **G** | **Log-Likelihood** | **AIC** | **BIC** | **SABIC** | **Entropy** | **Class Proportion (%)** | | | | | | **Average Posterior Probability** | | | | | |
| --- | --- | --- | --- | --- | --- | --- | --- | --- | --- | --- | --- | --- | --- | --- | --- | --- | --- |
|  |  |  |  |  |  | **1** | **2** | **3** | **4** | **5** | **6** | **1** | **2** | **3** | **4** | **5** | **6** |
| 2 | -18725.06 | 37480.12 | 37539.30 | 37491.71 | 0.82 | 90.84 | 9.16 |  |  |  |  | 0.97 | 0.91 |  |  |  |  |
| 3 | -18699.76 | 37439.51 | 37518.42 | 37454.97 | 0.72 | 48.69 | 8.64 | 42.67 |  |  |  | 0.84 | 0.96 | 0.87 |  |  |  |
| 4 | -18700.45 | 37450.91 | 37549.54 | 37470.22 | 0.68 | 66.75 | 18.06 | 11.78 | 3.40 |  |  | 0.86 | 0.77 | 0.79 | 0.86 |  |  |
| 5 | -18691.74 | 37443.49 | 37561.85 | 37466.67 | 0.59 | 37.17 | 31.68 | 16.23 | 2.36 | 12.57 |  | 0.78 | 0.65 | 0.74 | 0.94 | 0.80 |  |
| 6 | -18697.92 | 37465.84 | 37603.93 | 37492.88 | 0.47 | 39.01 | 0.00 | 26.70 | 15.18 | 4.19 | 14.92 | 0.71 |  | 0.47 | 0.64 | 0.90 | 0.65 |

# Supplementary Table 6H. Model Fit Statistics for 120-Hour ICP Trajectories in Elderly Patients in the eICU Dataset

| **G** | **Log-Likelihood** | **AIC** | **BIC** | **SABIC** | **Entropy** | **Class Proportion (%)** | | | | | | **Average Posterior Probability** | | | | | |
| --- | --- | --- | --- | --- | --- | --- | --- | --- | --- | --- | --- | --- | --- | --- | --- | --- | --- |
|  |  |  |  |  |  | **1** | **2** | **3** | **4** | **5** | **6** | **1** | **2** | **3** | **4** | **5** | **6** |
| 2 | -35124.31 | 70278.63 | 70337.81 | 70290.22 | 0.85 | 92.67 | 7.33 |  |  |  |  | 0.97 | 0.87 |  |  |  |  |
| 3 | -35099.61 | 70239.21 | 70318.12 | 70254.67 | 0.65 | 44.76 | 50.00 | 5.24 |  |  |  | 0.81 | 0.85 | 0.92 |  |  |  |
| 4 | -35093.67 | 70237.33 | 70335.97 | 70256.65 | 0.57 | 44.76 | 39.53 | 11.52 | 4.19 |  |  | 0.78 | 0.70 | 0.76 | 0.93 |  |  |
| 5 | -35087.75 | 70235.50 | 70353.86 | 70258.68 | 0.58 | 46.86 | 35.08 | 8.38 | 2.88 | 6.81 |  | 0.75 | 0.68 | 0.83 | 0.92 | 0.79 |  |
| 6 | -35080.32 | 70230.64 | 70368.73 | 70257.68 | 0.62 | 50.26 | 14.40 | 23.30 | 1.83 | 2.88 | 7.33 | 0.78 | 0.59 | 0.70 | 0.92 | 0.92 | 0.78 |

# Supplementary Table 6I. Model Fit Statistics for 168-Hour ICP Trajectories in Elderly Patients in the eICU Dataset

| **G** | **Log-Likelihood** | **AIC** | **BIC** | **SABIC** | **Entropy** | **Class Proportion (%)** | | | | | | **Average Posterior Probability** | | | | | |
| --- | --- | --- | --- | --- | --- | --- | --- | --- | --- | --- | --- | --- | --- | --- | --- | --- | --- |
|  |  |  |  |  |  | **1** | **2** | **3** | **4** | **5** | **6** | **1** | **2** | **3** | **4** | **5** | **6** |
| 2 | -23644.76 | 47319.53 | 47378.71 | 47331.12 | 0.88 | 92.15 | 7.85 |  |  |  |  | 0.98 | 0.84 |  |  |  |  |
| 3 | -23628.13 | 47296.25 | 47375.16 | 47311.70 | 0.59 | 41.62 | 51.05 | 7.33 |  |  |  | 0.76 | 0.82 | 0.83 |  |  |  |
| 4 | -23624.08 | 47298.16 | 47396.79 | 47317.47 | 0.52 | 35.86 | 29.84 | 28.27 | 6.02 |  |  | 0.73 | 0.63 | 0.74 | 0.81 |  |  |
| 5 | -23627.26 | 47314.52 | 47432.88 | 47337.69 | 0.52 | 43.98 | 8.64 | 19.63 | 3.40 | 24.35 |  | 0.76 | 0.56 | 0.53 | 0.78 | 0.73 |  |
| 6 | -23622.30 | 47314.61 | 47452.70 | 47341.65 | 0.57 | 43.46 | 9.16 | 21.20 | 20.94 | 4.19 | 1.05 | 0.76 | 0.61 | 0.56 | 0.71 | 0.81 | 0.82 |

# Supplementary Table 6J. Model Fit Statistics for 72-Hour ICP Trajectories in Elderly Patients in the NSICU Dataset

| **G** | **Log-Likelihood** | **AIC** | **BIC** | **SABIC** | **Entropy** | **Class Proportion (%)** | | | | | | **Average Posterior Probability** | | | | | |
| --- | --- | --- | --- | --- | --- | --- | --- | --- | --- | --- | --- | --- | --- | --- | --- | --- | --- |
|  |  |  |  |  |  | **1** | **2** | **3** | **4** | **5** | **6** | **1** | **2** | **3** | **4** | **5** | **6** |
| 2 | -6976.20 | 13982.40 | 14026.95 | 13979.48 | 0.81 | 78.47 | 21.53 |  |  |  |  | 0.96 | 0.95 |  |  |  |  |
| 3 | -6966.18 | 13972.36 | 14031.76 | 13968.47 | 0.82 | 77.08 | 3.47 | 19.44 |  |  |  | 0.94 | 0.89 | 0.89 |  |  |  |
| 4 | -6959.55 | 13969.10 | 14043.34 | 13964.23 | 0.74 | 50.69 | 25.69 | 20.14 | 3.47 |  |  | 0.89 | 0.81 | 0.89 | 0.91 |  |  |
| 5 | -6955.53 | 13971.07 | 14060.16 | 13965.24 | 0.74 | 50.00 | 25.00 | 16.67 | 3.47 | 4.86 |  | 0.89 | 0.79 | 0.81 | 0.83 | 0.76 |  |
| 6 | -6952.65 | 13975.31 | 14079.25 | 13968.50 | 0.78 | 11.81 | 49.31 | 25.00 | 6.94 | 2.08 | 4.86 | 0.78 | 0.90 | 0.79 | 0.84 | 0.97 | 0.89 |

# Supplementary Table 6K. Model Fit Statistics for 120-Hour ICP Trajectories in Elderly Patients in the NSICU Dataset

| **G** | **Log-Likelihood** | **AIC** | **BIC** | **SABIC** | **Entropy** | **Class Proportion (%)** | | | | | | **Average Posterior Probability** | | | | | |
| --- | --- | --- | --- | --- | --- | --- | --- | --- | --- | --- | --- | --- | --- | --- | --- | --- | --- |
|  |  |  |  |  |  | **1** | **2** | **3** | **4** | **5** | **6** | **1** | **2** | **3** | **4** | **5** | **6** |
| 2 | -12379.04 | 24788.09 | 24832.63 | 24785.17 | 0.84 | 77.78 | 22.22 |  |  |  |  | 0.97 | 0.96 |  |  |  |  |
| 3 | -12357.42 | 24754.83 | 24814.23 | 24750.94 | 0.85 | 74.31 | 10.42 | 15.28 |  |  |  | 0.95 | 0.98 | 0.86 |  |  |  |
| 4 | -12355.18 | 24760.35 | 24834.60 | 24755.49 | 0.83 | 71.53 | 12.50 | 11.81 | 4.17 |  |  | 0.96 | 0.79 | 0.82 | 0.92 |  |  |
| 5 | -12348.37 | 24756.75 | 24845.84 | 24750.91 | 0.80 | 17.36 | 52.78 | 14.58 | 2.78 | 12.50 |  | 0.82 | 0.89 | 0.80 | 0.95 | 0.96 |  |
| 6 | -12345.93 | 24761.86 | 24865.81 | 24755.06 | 0.80 | 15.28 | 53.47 | 4.17 | 11.81 | 2.78 | 12.50 | 0.83 | 0.88 | 0.68 | 0.80 | 0.93 | 0.95 |

# Supplementary Table 6L. Model Fit Statistics for 168-Hour ICP Trajectories in Elderly Patients in the NSICU Dataset

| **G** | **Log-Likelihood** | **AIC** | **BIC** | **SABIC** | **Entropy** | **Class Proportion (%)** | | | | | | **Average Posterior Probability** | | | | | |
| --- | --- | --- | --- | --- | --- | --- | --- | --- | --- | --- | --- | --- | --- | --- | --- | --- | --- |
|  |  |  |  |  |  | **1** | **2** | **3** | **4** | **5** | **6** | **1** | **2** | **3** | **4** | **5** | **6** |
| 2 | -8202.19 | 16434.38 | 16478.92 | 16431.46 | 0.80 | 72.92 | 27.08 |  |  |  |  | 0.96 | 0.95 |  |  |  |  |
| 3 | -8183.48 | 16406.97 | 16466.36 | 16403.08 | 0.86 | 70.83 | 7.64 | 21.53 |  |  |  | 0.96 | 0.94 | 0.91 |  |  |  |
| 4 | -8171.08 | 16392.16 | 16466.40 | 16387.30 | 0.87 | 66.67 | 16.67 | 7.64 | 9.03 |  |  | 0.95 | 0.86 | 0.87 | 0.96 |  |  |
| 5 | -8170.47 | 16400.93 | 16490.03 | 16395.10 | 0.79 | 50.69 | 21.53 | 2.78 | 5.56 | 19.44 |  | 0.89 | 0.79 | 0.86 | 0.96 | 0.88 |  |
| 6 | -8161.15 | 16392.30 | 16496.25 | 16385.50 | 0.78 | 48.61 | 20.14 | 13.89 | 2.78 | 7.64 | 6.94 | 0.87 | 0.78 | 0.84 | 0.82 | 0.95 | 0.90 |

# Supplementary Table 7. Association of ICP Trajectories with In-Hospital Mortality in Elderly Patients Across Cohorts

|  | **Merged data** | | | | **MIMIC-IV** | | | | **eICU** | | | | **NSICU** | | | |
| --- | --- | --- | --- | --- | --- | --- | --- | --- | --- | --- | --- | --- | --- | --- | --- | --- |
| **ICP Trajectories** | **Model 1** | | **Model 2** | | **Model 1** | | **Model 2** | | **Model 1** | | **Model 2** | | **Model 1** | | **Model 2** | |
|  | **HR（95%CI）** | ***P*** | **HR（95%CI）** | ***P*** | **HR（95%CI）** | ***P*** | **HR（95%CI）** | ***P*** | **HR（95%CI）** | ***P*** | **HR（95%CI）** | ***P*** | **HR（95%CI）** | ***P*** | **HR（95%CI）** | ***P*** |
| **Trajectory 1** | Ref | Ref | Ref | Ref | Ref | Ref | Ref | Ref | Ref | Ref | Ref | Ref | Ref | Ref | Ref | Ref |
| **Trajectory 2** | 1.28 (0.99~1.67) | 0.061 | 1.41 (1.05~1.89) | 0.022 | 1.41 (0.97~2.06) | 0.077 | 1.71 (1.13~2.61) | 0.013 | 0.86 (0.57~1.3) | 0.482 | 0.79 (0.51~1.23) | 0.296 | 2.84 (0.52~15.51) | 0.228 | 10.35 (2.11~50.83) | 0.004 |
| **Trajectory 3** | 2.37 (1.69~3.33) | <0.001 | 2.87 (1.97~4.2) | <0.001 | 9.95 (4.81~20.59) | <0.001 | 7.72 (3.38~17.62) | <0.001 | 2.24 (1.34~3.75) | 0.002 | 2.04 (1.12~3.73) | 0.022 | 13.24 (3.3~53.09) | <0.001 | 9.12 (2.64~31.51) | <0.001 |
| **Trajectory 4** | 7.1 (4.53~11.12) | <0.001 | 9.05 (5.46~15) | <0.001 | 23.13 (9.16~58.4) | <0.001 | 23.17 (8.43~63.64) | <0.001 | 6.08 (2.81~13.14) | <0.001 | 7.15 (3.08~16.63) | <0.001 | 99.38 (9~1097.43) | <0.001 | 3.9 (0.4~37.78) | 0.241 |

**Notes:** Association between ICP trajectory and in-hospital mortality in patients aged ≥55 years, analyzed using Cox regression. Model 1 is unadjusted. Model 2 is adjusted for Age, Heart Rate, Temperature, Urine Output, Creatinine, WBC, Admission Type, Traumatic etiology, HBP, Initial GCS, Intubation, Vasopressor, Craniotomy, and Embolization. Trajectory 1 is the reference group.

# Supplementary Table 8. Predictors of In-Hospital Mortality Identified by Stepwise Regression Analysis

| **Variable** | **Estimate** | **HR** | **SE** | **Z** | ***P*** | **AIC** |
| --- | --- | --- | --- | --- | --- | --- |
| **Age** | 0.023 | 1.023 | 0.004 | 6.459 | <0.001 | 5714.393 |
| **BMI** | -4.35E-04 | 1 | 0 | 2.03 | 0.042 |  |
| **Heart Rate** | 0.007 | 1.007 | 0.002 | 3.495 | <0.001 |  |
| **Temperature** | -0.18 | 0.835 | 0.049 | -3.685 | <0.001 |  |
| **Urine Output** | -5.06E-05 | 1 | 0 | -4.925 | <0.001 |  |
| **BUN** | 0.011 | 1.011 | 0.004 | 2.651 | 0.008 |  |
| **Creatinine** | 0.005 | 1.005 | 0.003 | 1.587 | 0.113 |  |
| **WBC** | 0.031 | 1.032 | 0.006 | 5.584 | <0.001 |  |
| **Traumatic** | -0.211 | 0.81 | 0.133 | -1.59 | 0.112 |  |
| **Hypertension** | 0.334 | 1.396 | 0.116 | 2.873 | 0.004 |  |
| **Initial GCS** | -0.035 | 0.966 | 0.014 | -2.472 | 0.013 |  |
| **Intubation** | 0.235 | 1.264 | 0.115 | 2.036 | 0.042 |  |
| **Vasopressor** | 0.408 | 1.505 | 0.112 | 3.657 | <0.001 |  |
| **Dialysis** | 0.683 | 1.979 | 0.26 | 2.627 | 0.009 |  |
| **Mannitol** | 0.63 | 1.877 | 0.123 | 5.121 | <0.001 |  |
| **Embolization** | -0.549 | 0.578 | 0.131 | -4.178 | <0.001 |  |

**Notes:** The variables shown were retained as predictors of in-hospital mortality from a stepwise Cox regression model. The final model was selected based on minimizing the AIC. Estimates with an absolute value less than 0.001 are presented in scientific notation. Variables retained in the final model include Age, BMI, Heart Rate, Temperature, Urine Output, BUN, Creatinine, WBC, Traumatic, Hypertension, Initial GCS, Intubation, Vasopressor, Dialysis, Mannitol, and Embolization.

# Supplementary Figure 9. LASSO Feature Selection for In-Hospital Mortality Prediction


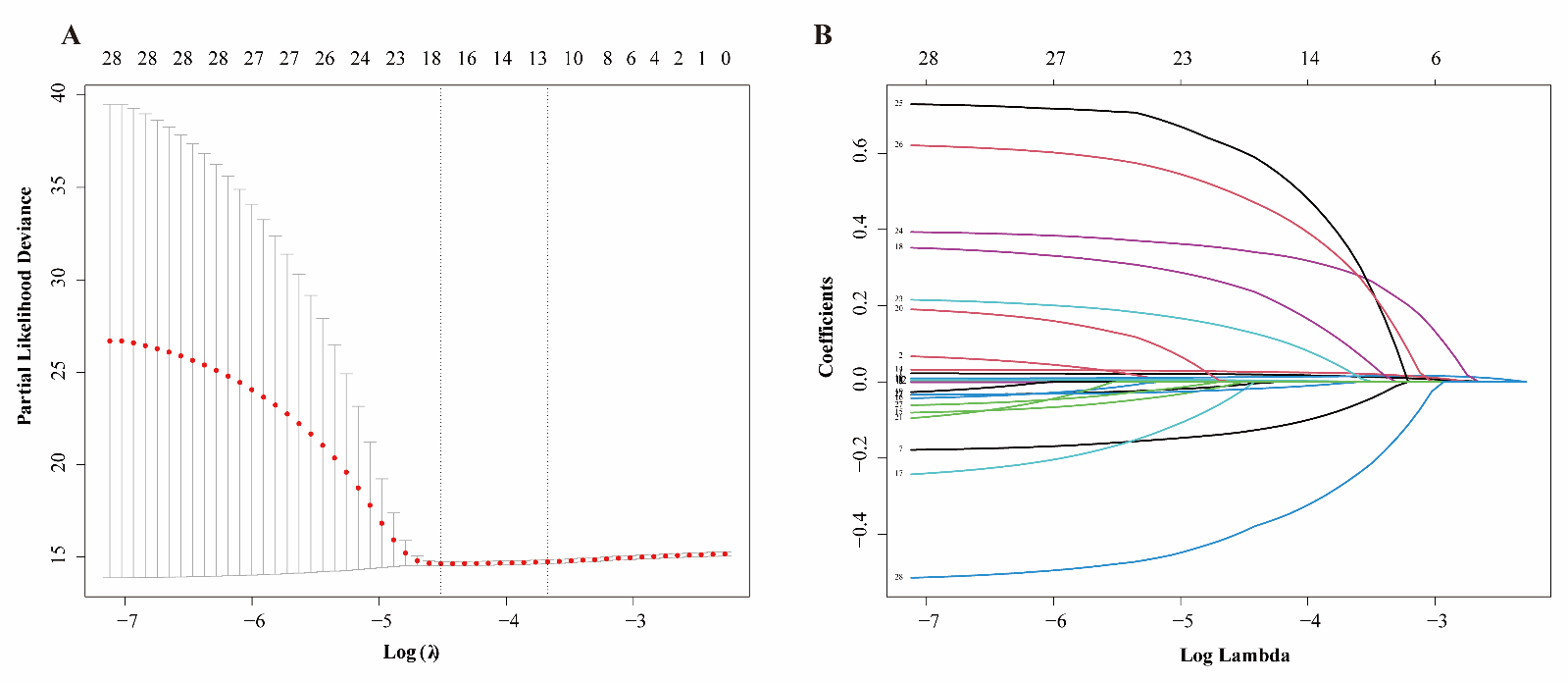


**Legend:** Feature selection via LASSO Cox regression. (A) Tenfold cross-validation for tuning the λ parameter, with lambda.1se (right vertical line) chosen for model parsimony. (B) Coefficient shrinkage paths. The 13 variables retained at lambda.1se are: Age, Heart Rate, Temperature, BUN, WBC, Urine Output, Vasopressor, Dialysis, Mannitol, Embolization, Initial GCS, Hypertension, and Intubation.

# Supplementary Figure 10. Model Selection Using Mallows’ Cp and Adjusted R² for In-Hospital Mortality Prediction


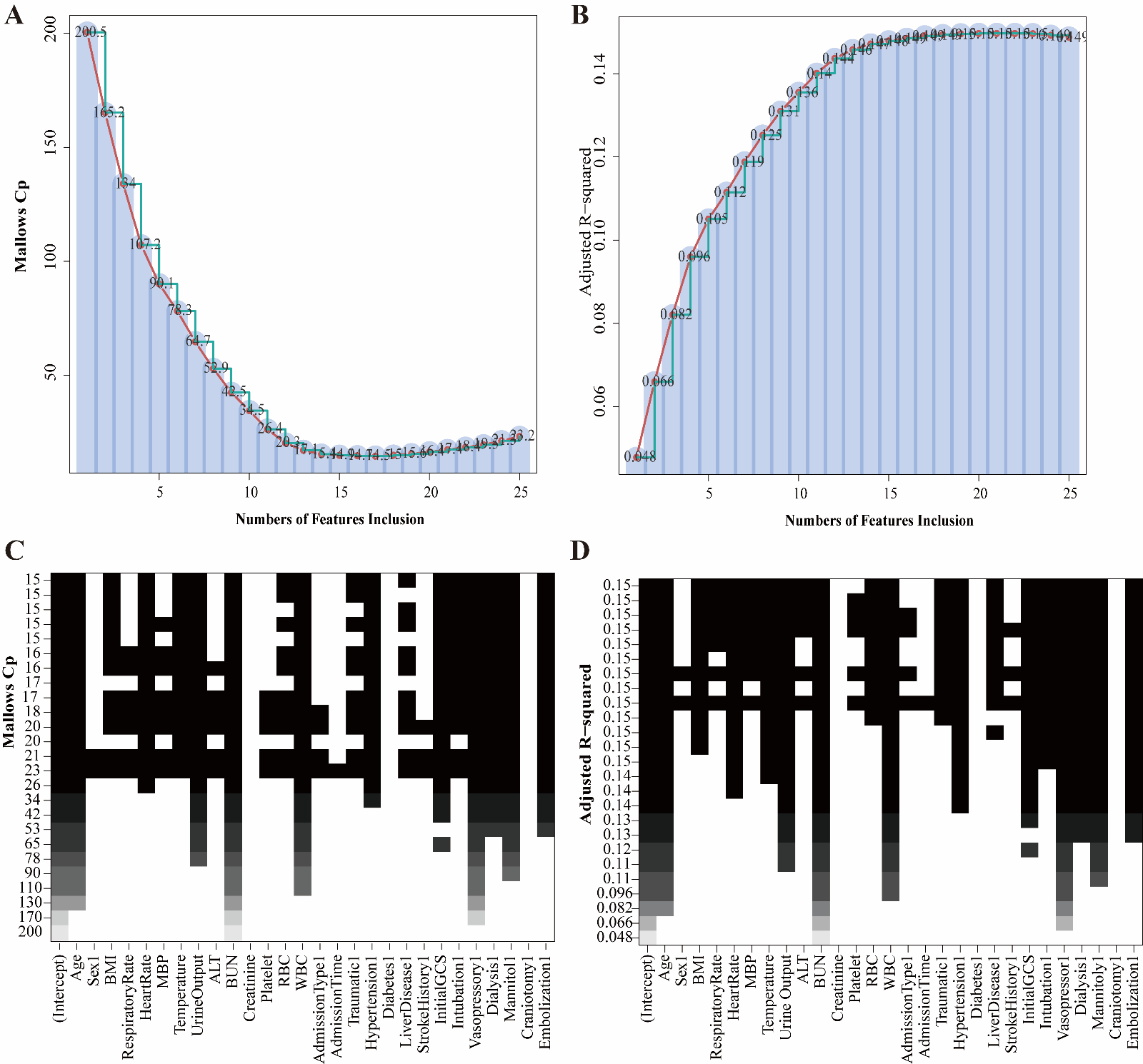
 **Legend:** Mallows’ Cp (A, C): The optimal model is identified by the minimum Cp value in panel (A). The 17 variables selected by this method, visualized in panel (C), are: Age, BMI, Heart Rate, Temperature, Urine Output, BUN, RBC, WBC, Traumatic, Hypertension, Liver Disease, Initial GCS, Intubation, Vasopressor, Dialysis, Mannitol, and Embolization. Adjusted R² (B, D): The optimal model is identified by the maximum R² value in panel (B). The 20 variables selected by this method, visualized in panel (D), are: Age, BMI, Respiratory Rate, Heart Rate, MBP, Temperature, Urine Output, ALT, BUN, RBC, WBC, Traumatic, Hypertension, Liver Disease, Initial GCS, Intubation, Vasopressor, Dialysis, Mannitol, and Embolization.

# Supplementary Figure 11. Boruta Feature Selection and Variable Overlap for In-Hospital Mortality Prediction


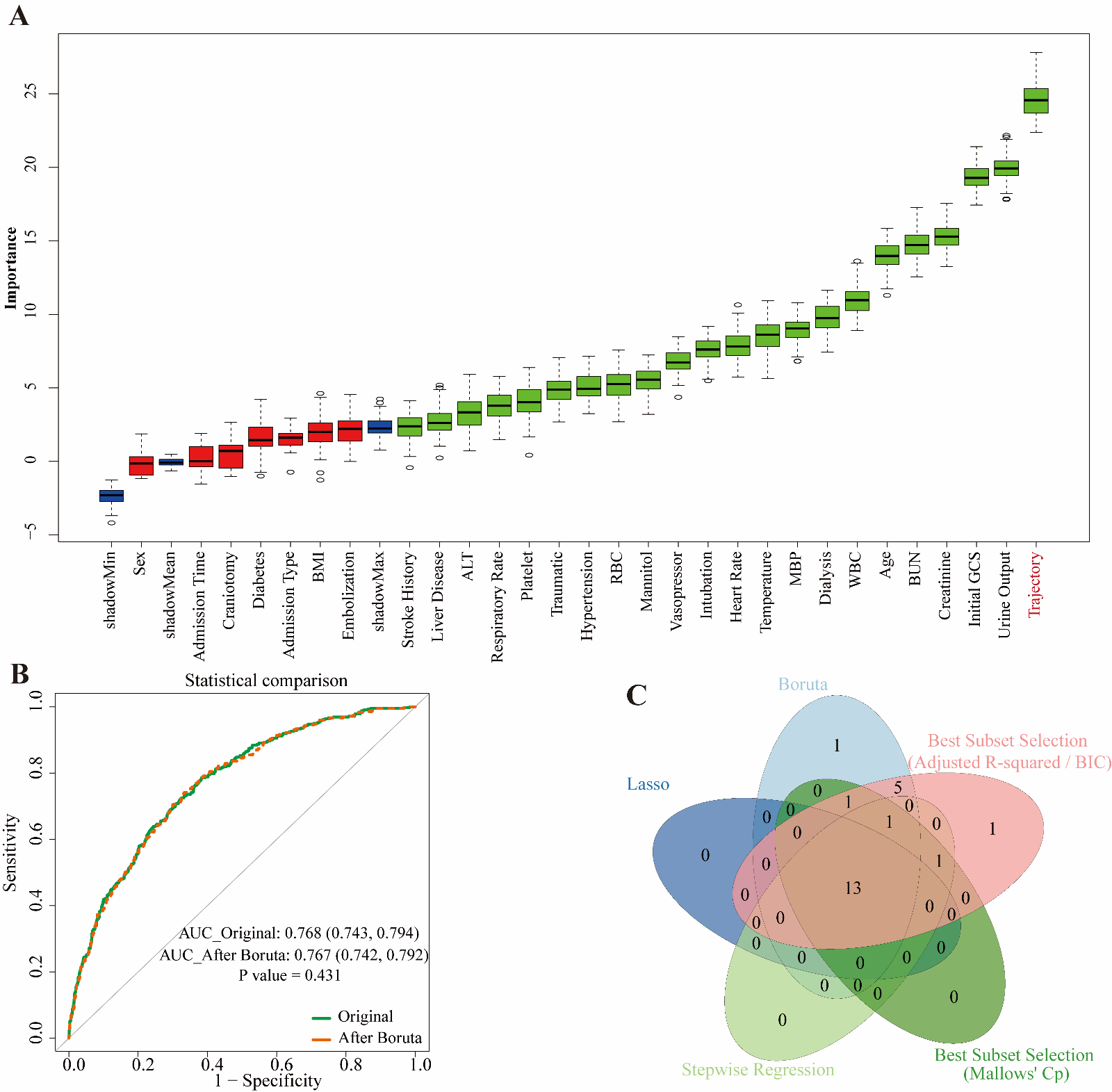


**Legend:** (A) Boruta Feature Importance: Features are classified as important (green), rejected (red), or tentative (blue) based on their importance scores relative to shadow attributes. (B) Model Performance Comparison: ROC curves for the mortality prediction model using the full feature set (Original) versus the subset selected by Boruta (After Boruta). The AUCs with 95% CIs and the p-value from the comparison test are displayed. (C) Feature Selection Overlap: A Venn diagram illustrating the number of shared variables selected by five different methods: Boruta, LASSO, Stepwise Regression, and Best Subset Selection optimized with two different criteria (Adjusted R² and Mallows’ Cp).

# Supplementary Figure 12. ROC Curves for Mortality Prediction Models
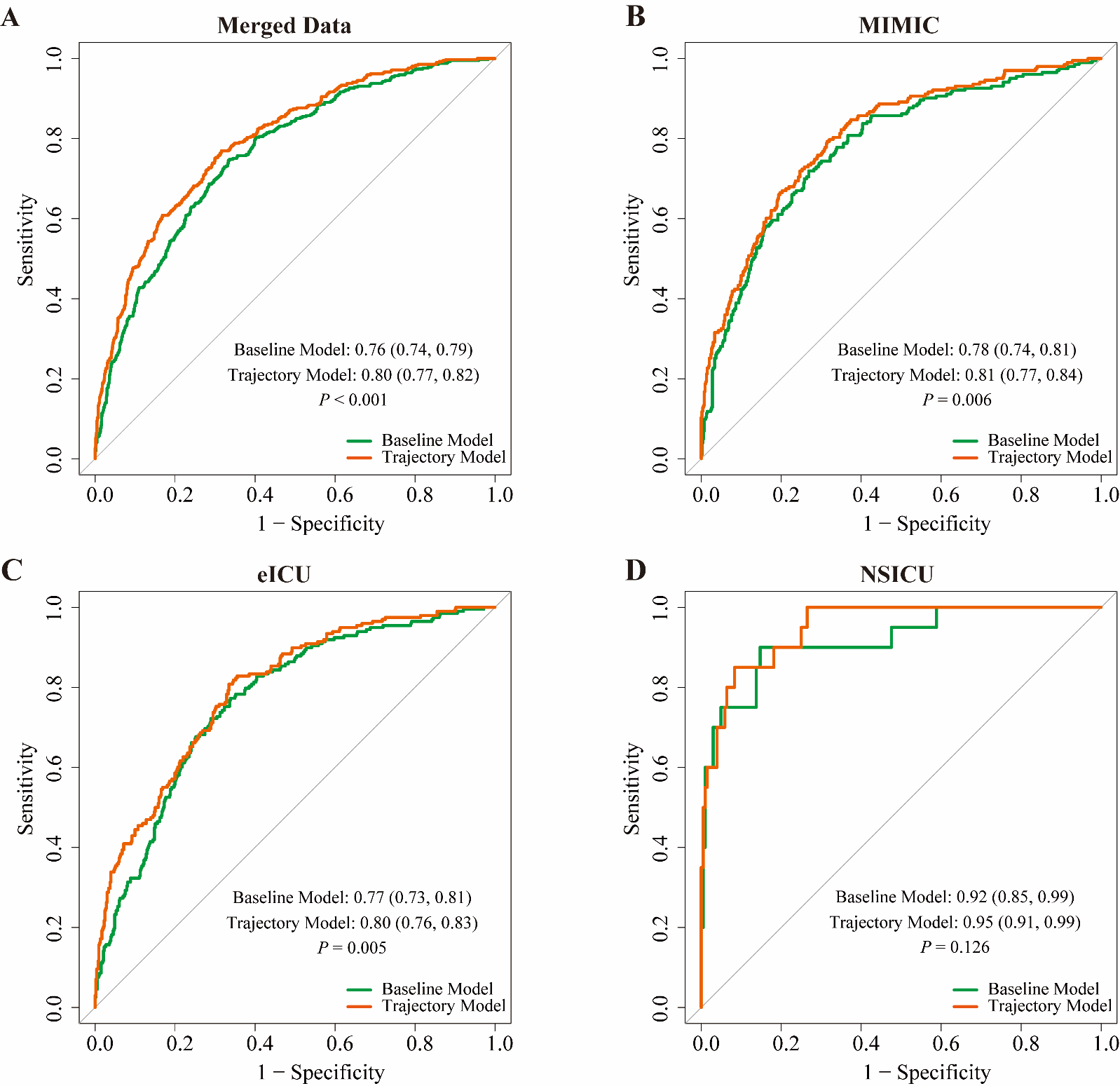


**Legend:** ROC curves comparing a Baseline Model and a Trajectory Model (Baseline + ICP trajectories) for predicting in-hospital mortality. The analysis is shown for the merged dataset (A) and the individual MIMIC (B), eICU (C), and NSICU (D) cohorts. The Baseline Model included Age, Heart Rate, Temperature, Urine Output, BUN, WBC, Hypertension, Initial GCS, Intubation, Vasopressor, Dialysis, Mannitol, and Embolization. The AUC with 95% CI is displayed for each model, along with the p-value from the comparison test.

# Supplementary Figure 13. Risk Reclassification After Incorporating ICP Trajectories


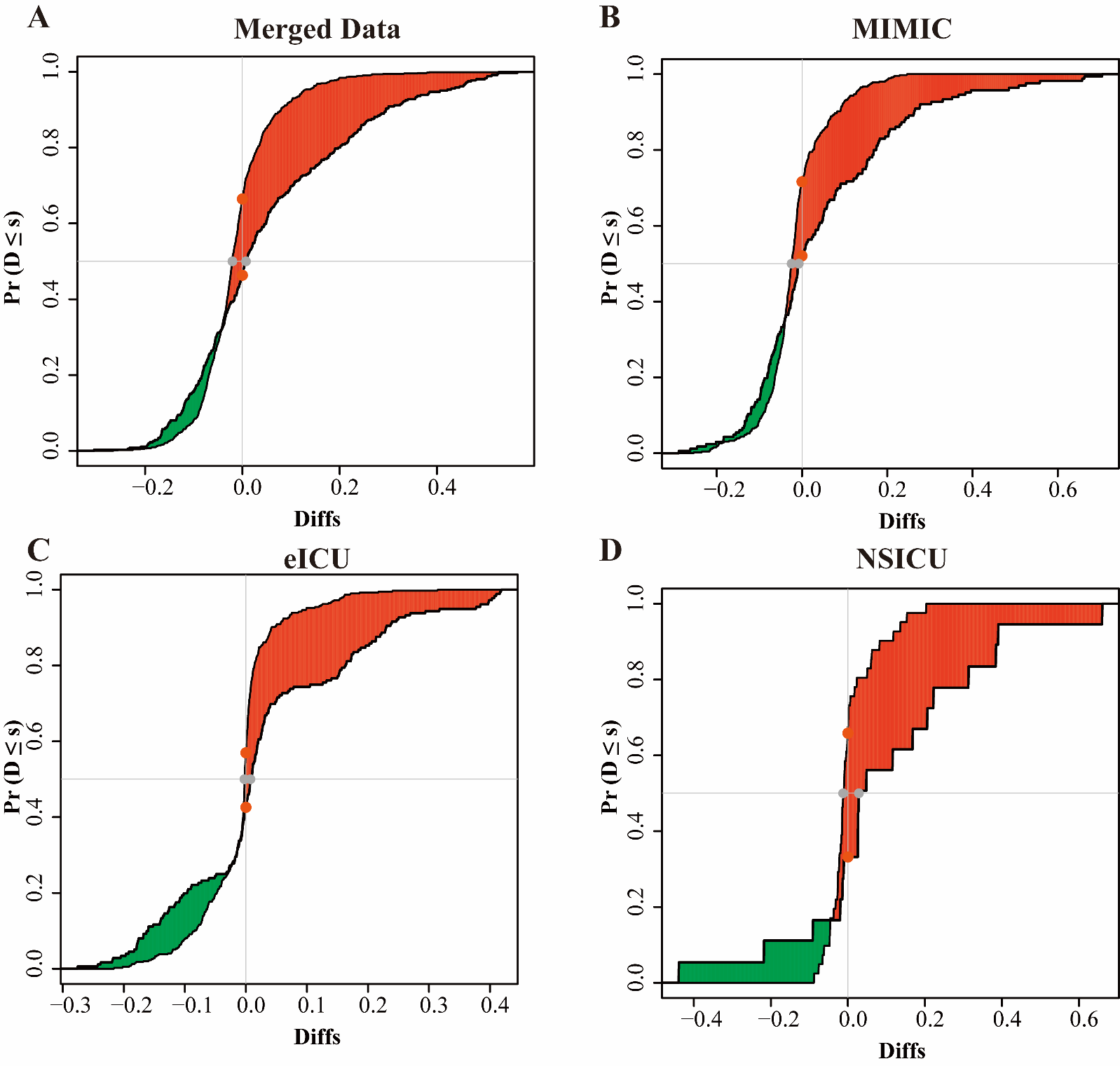


**Legend:** Risk reclassification plots for a Baseline Model vs. a model with added ICP trajectories, shown for the merged (A), MIMIC (B), eICU (C), and NSICU (D) cohorts. The Baseline Model included Age, Heart Rate, Temperature, Urine Output, BUN, WBC, Hypertension, Initial GCS, Intubation, Vasopressor, Dialysis, Mannitol, and Embolization. Shaded areas represent IDI components (positive in red, negative in green); orange points indicate cNRI; and grey markers show the median risk change.

# Supplementary Figure 14. SHAP-based Clinical Interpretability of the Logistic Mortality Models
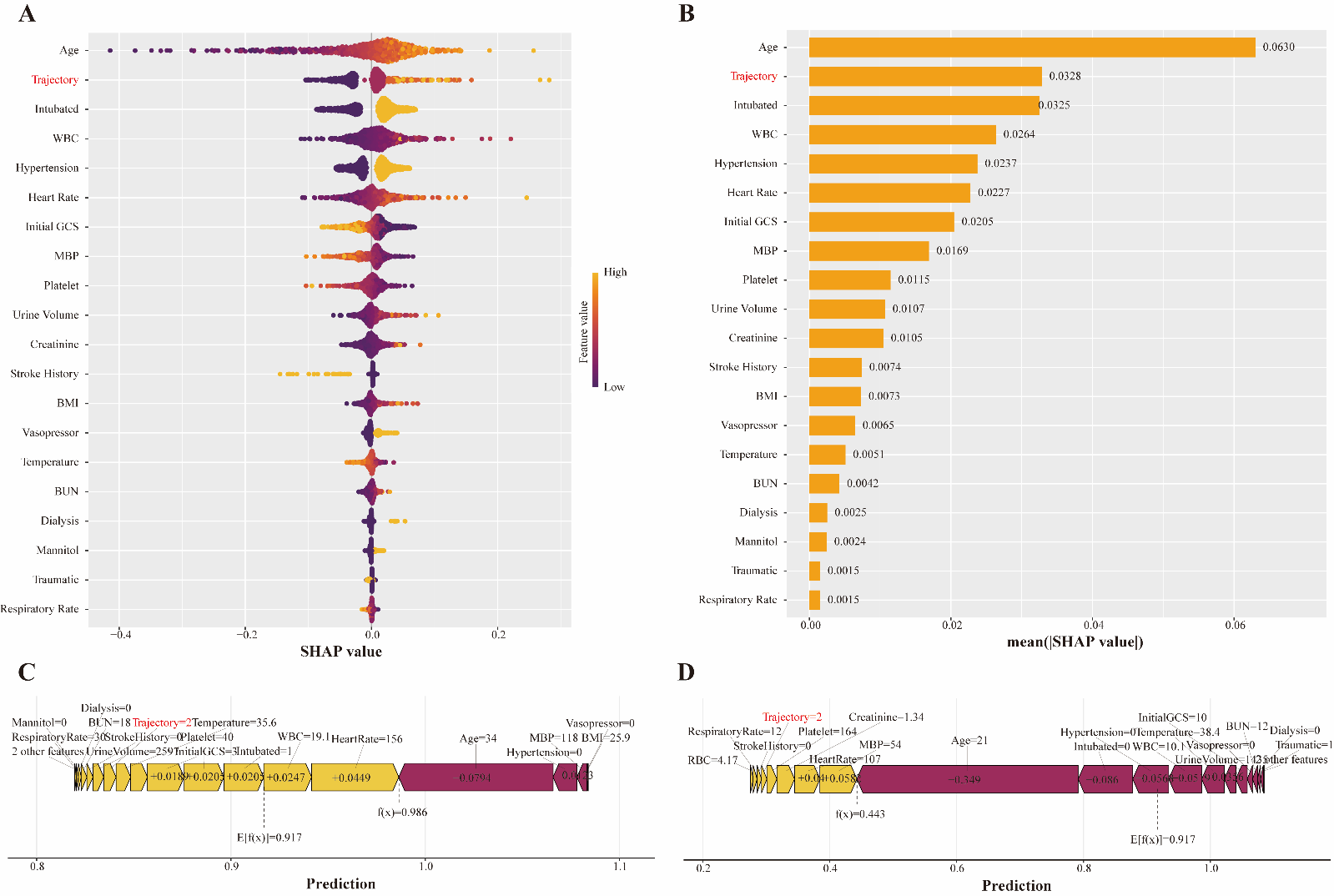


**Legend:** SHAP explanations for the logistic regression model that includes both clinical covariates and the ICP trajectory class. (A) SHAP summary plot: Each point represents the impact of a feature for a single patient. Color corresponds to the feature's value (high or low). (B) Global feature importance: Features are ranked by their mean absolute SHAP value. (C, D) Representative force plots: Illustrate how features contribute to pushing an individual patient's prediction away from the baseline expectation.
